# Shared molecular genetic factors influence subcortical brain morphometry and Parkinson’s disease risk

**DOI:** 10.1038/s41531-023-00515-y

**Published:** 2023-05-10

**Authors:** Luis M. García-Marín, Paula Reyes-Pérez, Santiago Diaz-Torres, Alejandra Medina-Rivera, Nicholas G. Martin, Brittany L. Mitchell, Miguel E. Rentería

**Affiliations:** 1 Mental Health and Neuroscience Program, QIMR Berghofer Medical Research Institute, Brisbane, QLD Australia; 2 School of Biomedical Sciences, Faculty of Medicine, The University of Queensland, Brisbane, QLD Australia; 3 Laboratorio Internacional de Investigación del Genoma Humano, Universidad Nacional Autónoma de México, Juriquilla, Querétaro México

**Keywords:** Genome-wide association studies, Computational neuroscience

## Abstract

Parkinson’s disease (PD) is a late-onset and genetically complex neurodegenerative disorder. Here we sought to identify genes and molecular pathways underlying the associations between PD and the volume of ten brain structures measured through magnetic resonance imaging (MRI) scans. We leveraged genome-wide genetic data from several cohorts, including the International Parkinson’s Disease Genomics Consortium (IPDG), the UK Biobank, the Adolescent Brain Cognitive Development (ABCD) study, the Cohorts for Heart and Aging Research in Genomic Epidemiology (CHARGE), the Enhancing Neuroimaging Genetics through Meta-Analyses (ENIGMA), and 23andMe. We observed significant positive genetic correlations between PD and intracranial and subcortical brain volumes. Genome-wide association studies (GWAS) - pairwise analyses identified 210 genomic segments with shared aetiology between PD and at least one of these brain structures. Pathway enrichment results highlight potential links with chronic inflammation, the hypothalamic-pituitary-adrenal pathway, mitophagy, disrupted vesicle-trafficking, calcium-dependent, and autophagic pathways. Investigations for putative causal genetic effects suggest that a larger putamen volume could influence PD risk, independently of the potential causal genetic effects of intracranial volume (ICV) on PD. Our findings suggest that genetic variants influencing larger intracranial and subcortical brain volumes, possibly during earlier stages of life, influence the risk of developing PD later in life.

## Introduction

Parkinson’s disease (PD) is a complex neurodegenerative disorder characterised by the accumulation of abnormal aggregates of the *α-synuclein* protein within the nerve cells, resulting in a progressive loss of dopaminergic neurons in the *substantia nigra*^1–3^. PD affects up to 2–3% of the population over 60 years of age worldwide^2,4,5^. In the United States alone, the economic burden of PD was estimated at around $51.9 billion in 2017^6^. The diagnosis of PD is primarily based on the development of motor symptoms such as rigidity, bradykinesia, resting tremor, and postural reflex disturbance^1^. Nonetheless, non-motor-related symptoms such as affective disorders, cognitive impairment, loss of sense of smell, and physical pain are also considered in clinical criteria used to diagnose PD^1^.

Neuroimaging techniques, such as magnetic resonance imaging (MRI), have been used to investigate PD’s pathology. Brain imaging uncovered neuroanatomical associations with PD. For instance, individuals with PD have been reported to have larger intracranial and thalamus volumes compared to healthy controls^7^. Previous studies have reported positive phenotypic correlations between PD and the volumes of the amygdala, brainstem, caudate nucleus, pallidum, putamen, and thalamus^8^. However, in the advanced stages of the disorder, individuals can experience volumetric reductions up to 30–40% in the entire thalamus^9–11^ and severe putamen atrophy in both volume and shape^12,13^.

Genetic epidemiological studies have aimed to uncover the genetic basis of PD, showing that up to 36% of the risk for idiopathic PD is heritable^14,15^. In addition, the largest meta-analysis of genome-wide association study (GWAS) of PD to date revealed 90 independent genetic variants associated with a higher risk for PD^14^. On the other hand, GWASs of subcortical brain structures have identified several genetic variants associated with brain morphometry^8,16^. These studies also reported positive genetic correlations between genetic susceptibility to PD and volumes of some subcortical structures, including the accumbens, brainstem, thalamus, and caudate nucleus^8^. These genetic correlations suggest that overlapping genetic variants and molecular pathways could influence PD risk and subcortical brain morphometry.

Although phenotypic and genome-wide genetic correlations between PD risk and brain structures have been described previously^8,17–19^, there is still a need to delineate the specific molecular and genetic pathways underlying these relationships to shed light on the underlying biological mechanisms influencing both PD and brain morphology at different stages in life. In the present study, we examine the genetic associations between PD and the volume of ten brain structures measured through MRI scans to identify segments of the genome with shared aetiology between PD and brain volumes.

## Results

### Genetic correlations

**Fig. 1:**
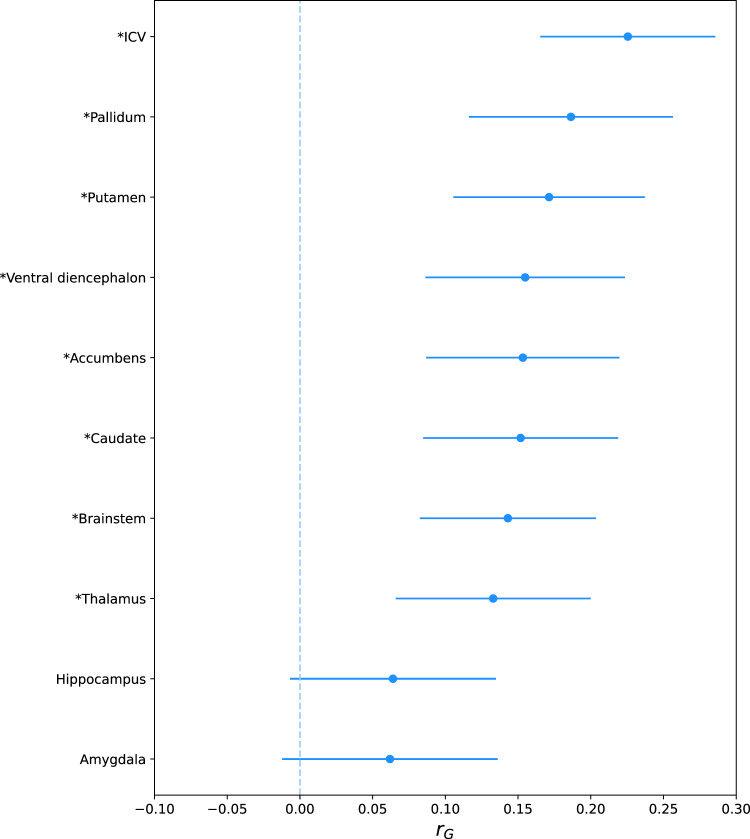
Linkage disequilibrium score regression (LDSC) estimates of the genetic correlation (rG) with 95% confidence intervals between Parkinson’s disease and the volume of ten brain structures adjusted for intracranial volume. Statistically significant genetic correlations (i.e., *P* < 0.05 / 10 [number of brain structures]) are marked with an asterisk (*). ICV Intracranial volume.

We examined the genetic overlap between PD and ten brain structures using linkage disequilibrium score regression (LDSC; Fig. 1). We observed the strongest genetic correlation between PD and intracranial volume (rG = 0.23, *P* = 2.15 × 10^−13^). In addition, we identified significant positive genetic correlations between PD and volume of the pallidum (rG = 0.19, *P* = 1.92 × 10^−07^), putamen (rG = 0.17, *P* = 3.48 × 10^−07^), brainstem (rG = 0.14, *P* = 3.73 × 10^−06^), nucleus accumbens (rG = 0.15, *P* = 5.95 × 10^−06^), caudate nucleus (rG = 0.15, *P* = 9.27 × 10^−06^), ventral diencephalon (rG = 0.15, *P* = 9.81 × 10^−06^), and the thalamus (rG = 0.13, *P* = 1.00 × 10^−04^). Genetic correlations between PD and the volume of the amygdala and hippocampus did not reach significance after multiple testing corrections.

### GWAS-pairwise

We further investigated the relationship between PD and the morphometry of the eight brain structures with a significant genetic correlation using GWAS-PW (i.e., excluded amygdala and hippocampus). We identified 210 genomic segments involved in the aetiology of PD and at least one of the eight brain structures via the same genetic variants (Supplementary Table 1). The exact number of shared genomic segments varied across brain structures (Table 1). Intracranial volume (ICV) shared the largest number of shared segments (*N* = 45) with PD, followed by the brainstem (39 segments), ventral diencephalon (28 segments), and putamen (23 segments). Furthermore, the pallidum, thalamus, and caudate nucleus each shared 21 genomic segments with PD. The accumbens, which shared 12 segments, was the subcortical brain structure that shared the fewest genomic segments with PD.

**Table 1:** Shared genomic segments between Parkinson’s disease and brain structures.

Brain structure	Shared genomic segments with Parkinson’s disease
ICV	45
Brainstem	39
Ventral diencephalon	28
Putamen	23
Pallidum	21
Thalamus	21
Caudate nucleus	21
Accumbens	12
Total	210

*ICV* Intracranial volume.

### Functional annotation

**Fig. 2:**
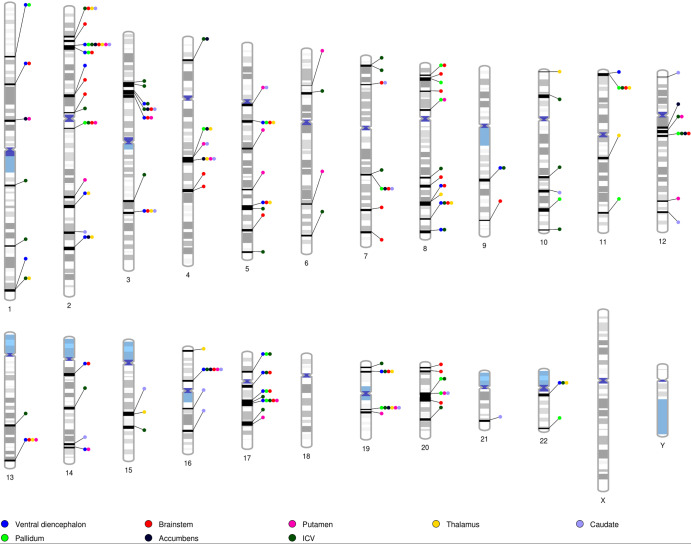
Ideogram highlighting shared genomic segments between brain structures and Parkinson’s disease. ICV Intracranial volume.

For each brain structure with shared genomic segments with PD, we mapped genetic variants in the identified genomic segments to protein-coding genes using MAGMA (Table 2). All brain structures and PD showed significantly enriched genes in the shared genomic segments after Bonferroni multiple testing correction (Supplementary Tables 2–9). Overall, 129 genes were significantly enriched in PD and at least one brain region (Table 1 and Fig. 2).

**Table 2:** Some genes in shared genomic segments are associated with both Parkinson’s disease and the volume of subcortical brain structures.

Gene associated with Parkinson’s disease	Brain structures associated with the gene	Biological relevance of the gene
*CRHR1*	• Putamen • Brainstem • Ventral diencephalon • ICV	Neuroprotective effect via regulation of the HPA axis and inflammatory response^14^.
*PLEKHM1*	• Putamen • Brainstem • Ventral diencephalon • ICV	Encodes a Rab7 effector protein that regulates the degradation of protein aggregates through lysosomal removal of endocytic and autophagic cargo^52,53^.
*MAPT*	• Putamen • Brainstem • Ventral diencephalon • ICV	Predominantly associated with brain volume loss in frontotemporal dementia due to tau protein aggregation^54^. This gene has also been associated with neurodegeneration^55^ and Parkinson’s disease^97^
*SPPL2C* and *NSF*	• Putamen • Brainstem • Ventral diencephalon • ICV	Process N-ethylmaleimide-sensitive factor attachment protein receptor (SNARE) proteins^56^ and disassemble the SNARE complex^57^. *NSF* has been implicated several times in neurodegeneration^98,99^.
*GPNMB*	• Caudate	Dysfunction of the autophagy-lysosomal pathway^36^ and a role in inflammatory response^14,35,37^.
*ATXN2L*	• Putamen • Caudate	Cognitive resilience^27^ and verbal-numerical reasoning^26^.
*ATP2A1*	• Putamen • Caudate	Dysregulation of calcium-dependent processes resulting in neural demise^29^.
*TUFM*	• Putamen • Caudate	Mitophagy and kinase activity reduction of LRRK2^27,28^.
*CD19* and *LAT*	• Putamen • Caudate	Peripheral inflammation in PD^31,32^.
*RABEP2* and *SPNS1*	• Putamen • Caudate	Disrupted vesicle trafficking^30^ and autophagic dysfunction^33^ leading to protein aggregation.

In the present study, the putamen and the caudate nucleus shared the enrichment of 28 and 27 genes, respectively, with PD. These brain structures were followed by the brainstem, ICV, ventral diencephalon, and pallidum, each sharing 23, 21, 13, and nine enriched genes with PD. We observed the lowest number of common enriched genes with PD in the thalamus and the accumbens, each with four.

Notably, the enrichment of the corticotropin-releasing hormone receptor 1 (*CRHR1*) gene on chromosome 17, which has been robustly associated with PD development, was shared between the putamen, brainstem, ICV, ventral diencephalon, and PD. Other genes enriched in these four brain structures and PD included rho GTPase activating protein 27 (*ARHGAP27)*, pleckstrin homology and RUN domain containing M1 *(PLEKHM1)*, signal peptide peptidase like 2C *(SPPL2C)*, microtubule associated protein tau (*MAPT)*, KAT8 regulatory NSL complex subunit 1 *(KANSL1)*, N-ethylmaleimide sensitive factor, vesicle fusing ATPase (*NSF)*, and wnt family member 3 (*WNT3)*. In addition, the expression of ataxin 2 like (*ATXN2L)*, tu translation elongation factor, mitochondrial (*TUFM)*, SH2B adaptor protein 1 *(SH2B1)*, ATPase sarcoplasmic/endoplasmic reticulum Ca2+ transporting 1 (*ATP2A1)*, rabaptin, RAB GTPase binding effector protein 2 (*RABEP2)*, CD19 molecule (*CD19)*, nuclear factor of activated T cells 2 interacting protein (*NFATC2IP)*, SPNS lysolipid transporter 1, lysophospholipid *(SPNS1)*, and linker for activation of T cells (*LAT)*, was enriched in the putamen, caudate nucleus, and PD.

A similar association was observed for the expression of glycoprotein nmb (*GPNMB)* on chromosome 7, which is also strongly associated with PD, and was shared between the caudate nucleus and PD. Furthermore, the caudate nucleus, pallidum, putamen, and PD showed enriched gene expression for histocompatibility minor 13 (*HM13)*, inhibitor of DNA binding 1 (*ID1)*, cytochrome c oxidase subunit 4I2 (*COX4I2)*, BCL2 like 1 (*BCL2L1)*, TPX2 microtubule nucleation factor (*TPX2)*, myosin light chain kinase 2 (*MYLK2)*, forkhead box S1 *(FOXS1)*, dual specificity phosphatase 15 (*DUSP15)*, and COMM domain containing 7 (*COMMD7)*.

### Genetic causal proportion

We performed latent causal variable analyses to investigate putative causal genetic effects between the volume of subcortical brain structures with a significant genetic correlation (*P* < 0.05) and PD (Supplementary Table 10). We observed a potential causal association in which a larger volume of the putamen influenced a higher risk for PD (GPC = −0.49, *P* = 2.00 × 10^−6^) after multiple testing corrections. Furthermore, we identified a nominal association (*P* < 0.05) in which a larger intracranial volume increased the risk for PD (GPC = −0.31, *P* = 4.06 × 10^−2^).

### Sensitivity analyses

#### Mendelian randomisation

Although we were unable to fulfill the assumption for independent samples required in Mendelian randomisation methods (see Discussion), we aimed to use a Mendelian randomisation approach as a sensitivity analysis to explore the putative causal genetic effects between the volume of subcortical brain structures with a significant genetic correlation (*P* < 0.05) and PD. We identified potential causal genetics effects from intracranial and five subcortical brain structures (putamen, ventral diencephalon, pallidum, caudate, and brainstem) on PD using Generalised Summary-data-based Mendelian Randomisation (GSMR; Supplementary Table 11). We observed that ICV (OR = 1.69, *P* = 2.3 × 10^−32^) and the putamen (OR = 1.38, *P* = 2.5 × 10^−11^) held the largest and most significant potential causal genetic effects on PD. In addition, we observed small putative causal genetic effects of PD on the brainstem and the caudate (Supplementary Table 11).

We further tested those regions with a putative causal effect after Bonferroni multiple testing correction on PD while controlling for their respective potential confounding effects using multivariable Mendelian randomisation (MVMR). With this approach, all five subcortical brain structures still were causally associated with PD even after controlling for the confounding effect of ICV **(**Supplementary Table 12). Larger ICV remained a potential risk factor for PD after controlling for potential confounding effects of each subcortical brain region, except for the ventral diencephalon **(**Supplementary Table 12).

#### Genetic variants associated with ICV possibly increase the risk for Parkinson’s disease

We selected 74 independent SNPs to assess whether genetic variants that influence ICV could be causally associated with PD risk. The proportion of phenotypic variance (PVE) explained by the selected SNPs on ICV was 14.76%. Analysis with MR-PRESSO identified four potential pleiotropic outliers (rs11710076, rs62240962, rs73802707, and rs9267573). Once the outliers were excluded, MR results based on GCTA-GSMR showed that genetic predisposition to a larger ICV is likely to increase the risk for PD (GCTA-GSMR OR = 1.71; 95% CI = 1.56–1.87, *P* = 2.33 × 10^−32^, Supplementary Fig. 1 and Supplementary Fig. 2). In addition, these findings were replicated using MR-BASE providing similar results. For instance, the inverse variance weighted approach estimated an OR = 1.52 (95% CI = 1.37–1.52, *P* = 2.97 × 10^−15^). Supplementary Fig. 2 shows all the derived Mendelian randomisation estimates for the putative causal genetic effect of ICV on PD risk, and Supplementary Fig. 3 depicts the effect trend.

We observed no evidence of directional horizontal pleiotropy with the MR-Egger approach (MR-Egger intercept = 0.001, *P* = 0.83). However, we observed some evidence of heterogeneity between genetic variants (Q = 185.98, *i*^2^ = 63.4%, *P* = 6.06 × 10^−13^). Overall, we did not find evidence of unbalanced pleiotropy following an observational inspection of funnel plot symmetry (Supplementary Fig. 4). We further investigated whether the heterogeneity between instrumental variables influenced our results. Thus, we employed the Mendelian randomisation Rucker approach in which inverse variance weighted was selected as a robust method to account for the heterogeneity. We observed no evidence of reverse causality between PD and ICV in bidirectional GSMR analysis (Supplementary Table 11). All MR findings in the present study are reported per unit of change (cm^3^) in brain volume.

#### Genetic correlation and GWAS-pairwise analyses for Alzheimer’s disease and intracranial and subcortical brain volumes

As part of our sensitivity analyses, we investigated the genetic overlap between Alzheimer’s disease (AD) and intracranial and subcortical brain volumes in an effort to compare the changes in brain volume in PD with those in AD. We did not identify any statistically significant genetic correlations between AD and intracranial and subcortical brain volumes after multiple testing corrections **(**Supplementary Fig. 5 and Supplementary Table 13). Nonetheless, we identified a nominally significant (*P* < 0.05) genetic correlation between AD and the volume of the thalamus at the whole genome level. Furthermore, we performed GWAS-PW analyses for AD and ICV and nine subcortical brain volumes. We did not identify any genomic segments with genetic variants influencing the aetiology of both AD and the volume of at least one brain structure (Supplementary Table 14).

## Discussion

We examined the genetic overlap between PD and intracranial and subcortical brain structure volumes. Our findings show significant positive genetic overlap at the genome-wide level between PD and the morphometry of eight brain structures, including ICV, pallidum, putamen, ventral diencephalon, accumbens, caudate nucleus, brainstem, and thalamus. In contrast, we did not find evidence supporting significant genetic overlap of the hippocampus or the amygdala with PD. These findings suggest that common genetic effects across the genome partially explain the observed association between PD and the morphology of these eight brain structures. Further interrogation of the underlying genetic components between PD and brain structure uncovered 210 genomic segments influencing the aetiology of PD and at least one brain structure.

Consistent with findings previously reported in genome-wide association studies^8^, we identified positive genetic correlations between PD and intracranial and subcortical brain volumes, which are also consistent with some phenotypic observations in the early stages of the disorder^7,8^. We must consider that in the study of young- or late-onset phenotypes, there may exist a temporality difference between phenotype measurements. Nonetheless, the statistical genetics approach enables the assessment of how the genetic variants influencing a particular phenotype could influence the genetic susceptibility of another at a later stage in life^8,20^. Related to this is the fact that the GWAS summary statistics for intracranial and subcortical brain volumes used to estimate the genetic overlap with PD are not necessarily enriched for people diagnosed with PD or individuals at high risk. For instance, a portion of the sample included participants from the ABCD study, which is a longitudinal resource that includes 11,875 children aged nine and ten years old at recruitment^21^. In addition, most participants from other cohorts were under 60 years of age, which is considered a typical age of onset for PD^22^. Therefore, our results suggest that genetic variants contributing to a larger volume of certain brain structures, possibly in the early stages of life, might also confer higher genetic susceptibility to PD at an advanced age. Below, we discuss the biological relevance of the genes that were mapped to the genomic segments influencing the aetiology of PD and at least one brain structure.

Two of the most commonly associated brain structures with PD are the putamen and the caudate nucleus, which constitute a portion of deep brain nuclei known as the basal ganglia, whose circuitry is severely affected during PD. The primary function of the basal ganglia is to control conscious and proprioceptive movements; nonetheless, other functions associated with this region include motor learning, emotions, motivation, and executive functions and behaviours^23^. PD symptoms are well-known to manifest when the dopaminergic neurons in the basal ganglia become impaired or die. Specifically, PD is highly recognised as a putamen-related disorder since dopamine depletion in its posterior regions prompts PD’s development and manifests as rigidity, tremor, ataxia, and balance impairment, among other symptoms^24^. Although less severely affected in PD than the putamen, the caudate nucleus is implicated in a number of neurocognitive impairments. For instance, hampered activity in the caudate nucleus has been shown to manifest as deficits in working memory among individuals with PD^25^. In addition, detriments in neuropsychological performance and the degree to which dementia is developed during PD are associated with the severity of the loss of dopaminergic neurons in the caudate nucleus^25^.

Gene-based tests in shared genomic segments for PD and brain structures uncovered nine enriched genes in PD that were also enriched in the putamen and caudate nucleus. These included *ATXN2L* in chromosome 16, which has been associated with verbal-numerical reasoning^26^ and cognitive resilience^27^; *TUFM* with links to mitophagy and known to reduce kinase activity of *LRRK2*^27,28^*;* and *ATP2A1*, whose overexpression due to α-synuclein aggregates has been linked to the dysregulation of calcium-dependent processes in the aging brain, resulting in neural demise^29^. Further, other shared enriched genes included *RABEP2* involved in disrupted vesicle trafficking leading to abnormal protein aggregation^30^; *CD19* and *LAT* with links to peripheral inflammation in PD^31,32^; and *SPNS1*, which has been associated with autophagic dysfunction in animal models of PD and in brains of individuals who were diagnosed with PD^33^. Our findings suggest that, at a genomic level, the association between PD susceptibility and the morphology of the putamen and caudate could be explained through biological processes taking place as an individual ages, such as mitophagy or peripheral inflammation, and the disruption of calcium-dependent, protein aggregation, and autophagic pathways.

Previous studies have sought to determine to what extent PD could be considered a lipidopathy^34^. Among these studies, the potential role of the *GPNMB* gene in PD’s aetiology has been widely discussed^14,35,36^. Although *GPNMB* expression is commonly associated with low metastatic human melanoma cell lines, it has been suggested that genetic variation in *GPNMB* is linked with PD^37^. For instance, GWAS for PD have identified an association between PD and the SNP rs199347 in the *GPNMB* locus, suggesting that the increased expression of the gene *GPNMB* is related to PD via its role in neuroinflammatory response^14,35,37^. Interestingly, the polymorphisms in GPNMB that associate with PD are also linked to the function of the autophagic-lysosomal pathway, which in turn explains their association with lysosomal storage disorders^36^. Further studies have reported that GPNMB increments in the *substantia nigra* relate directly to lipidopathy changes due to glucocerebrosidase inhibition^37,38^. We identified the enrichment of *GPNMB* in PD and the caudate’s morphology, which is consistent with previously observed colocalization effects^39^. Our results suggest that the observed association between PD and the caudate nucleus could be explained by dysfunction of the autophagy-lysosomal pathway, potentially leading to alpha-synuclein aggregation^40^.

A predominant symptom of PD is chronic inflammation due to high cortisol levels^41^. In healthy individuals, when the hypothalamic-pituitary-adrenal (HPA) axis is stimulated, the adrenal glands release glucocorticoids. Shortly after, the glucocorticoid receptor (GR) is activated to regulate inflammatory response through direct transcriptional action on target genes and the indirect inhibition of transcriptional or interferon regulatory factors^41^. However, among individuals with PD, the HPA axis is unbalanced^41,42^, and deregulation of GR in immune cells induces a chronic inflammatory state. Elevated glucocorticoid levels are recognised as one of the leading causes of stress-induced neural death of dopaminergic neurons^43^. Notably, it has been reported that the *CRHR1* gene is associated with a decreased risk for PD^14^. This gene encodes a G-protein coupled receptor that binds to neuropeptides of the corticotropin-releasing hormone family, which are prominent regulators of the HPA axis. In particular, the CRHR1 protein plays a major role in the activation of signal transduction pathways regulating several physiological processes such as stress and immune response. For instance, one of the most promising characteristics of the CRHR1 receptor is its capacity to combine with the corticotropin-releasing factor to regulate concentrations of cortisol released, showing a neuroprotective effect that is most likely explained by the expression of Parkin through the cAMP Response Element-Binding Protein (CREB) pathway^44–46^. In the present study, the gene *CRHR1* was significantly enriched in PD, the putamen, brainstem, ventral diencephalon, and ICV. This finding suggests that the genetic variants associated with the neuroprotective effect of *CRHR1* play a major role in PD’s aetiology and also influence the morphology of the putamen, brainstem, ventral diencephalon, and ICV at an early age.

The *substantia nigra* is one of the main subcortical structures affected in PD and is located in the midbrain, which in turn is part of the brainstem^47,48^. Previous studies have observed associations between the brainstem and ICV with PD’s etiology^8^. For instance, it has been suggested that damage to dopaminergic neurons within the brainstem vagal circuits could contribute to sialorrhea, dysphagia, and gastrointestinal dysfunction in PD^49,50^. In addition, increments in ICV have been observed among individuals with PD, and it has been hypothesised that this association might be explained by genetic factors^18^. Furthermore, the ventral diencephalon, which includes the thalamus, epithalamus, subthalamus, and hypothalamus^51^, is one of the related nuclei to the basal ganglia, and its functional neuroanatomy suggests that damage to the diencephalon could influence PD’s symptoms^36^.

Further gene-based tests conducted on the genomic segments shared by PD and brain structures revealed seven additional enriched genes in PD and the putamen, brainstem, ventral diencephalon, and ICV. For instance, *PLEKHM1* encodes a Rab7 effector protein that regulates the degradation of protein aggregates through lysosomal removal of endocytic and autophagic cargo^52,53^. In addition, although *MAPT* is predominantly associated with brain volume loss in frontotemporal dementia due to tau protein aggregation^54^, it has been reported that regional increased *MAPT* expression in the brain is associated with selective vulnerability of functional brain networks to neurodegeneration^55^. Furthermore, in the present study, other enriched genes included *SPPL2C* and *NSF*, which process N-ethylmaleimide-sensitive factor attachment protein receptor (SNARE) proteins^56^ and disassemble the SNARE complex^57^, respectively. The SNARE complex participates in vesicle fusion and neurotransmitter release, and its dysfunction has been associated with neurodegeneration^58^.

We further explored genetic correlations with PD and tested the effect of the genetic liability for larger intracranial and subcortical brain volumes on PD using LCV, a method known to be robust for sample overlap^59^. Similarly, as a sensitivity analysis, we conducted Mendelian randomisation analyses. Our results support previous observations of individuals with PD having a larger ICV^18,60^. Nonetheless, until now, the directionality and investigation of potential causal effects of intracranial and subcortical brain volumes and PD were unclear. In this study, we provide evidence that genetic variants influencing larger intracranial and putamen volumes, potentially influence a higher risk of PD in late adulthood. Our sensitivity analyses suggest that the subcortical volumes of the ventral diencephalon, pallidum, caudate, and brainstem could also influence a higher risk of PD late in life; however, these effects were not observed with LCV analyses. Lastly, in the MR methods conducted after accounting for potential confounding effects between intracranial and subcortical brain volumes, larger volumes of the putamen, ventral diencephalon, pallidum, caudate, and brainstem remained potential risk factors of PD. Our findings suggest that a larger volume of intracranial and subcortical brain structures could influence the development of PD, independently of the potential causal genetic effects of ICV on a higher PD risk.

Regarding the sensitivity analyses performed for AD and intracranial and subcortical brain volumes, we note that neurodegeneration in PD is known to be prominent in subcortical brain structures^17^, particularly in the *substantia nigra*^3^, whereas neurodegeneration in AD occurs predominantly in the cortex and the hippocampus^61,62^. Despite the fact that in AD, the volume of the hippocampus is reduced, resulting in memory loss^63^, in the present study, we did not identify any genetic overlap between AD and intracranial or subcortical brain volumes. The GWAS summary statistics used for AD contain a larger number of proxy cases (i.e., first-degree relatives with AD) than diagnosed AD cases. For instance, one of the samples included in this meta-analysis is the UK Biobank, with 2447 diagnosed cases and 46,828 proxy cases of dementia^64^. Thus, it is possible that the specific role of the hippocampus in memory loss in AD is not entirely captured by these GWAS summary statistics, which contain a large number of proxy cases. We suggest that future studies seek to elucidate the genetic components that could influence the relationship between AD and the volume of the hippocampus. We also estimated the genetic overlap between body mass index (BMI) and ICV, as previous studies have suggested that BMI could be a genetic driver for ICV^65^. We did not identify genetic overlap between these phenotypes (rG = 0.008, *p*-value = 0.67).

We note that the GWAS results for the nine subcortical brain structures used in the present study were adjusted for the effects of ICV. Therefore, variants associated with subcortical structures in these summary statistics are independent of overall brain size. Nonetheless, the limitations of this study must be acknowledged. For instance, since the GWA studies used in this study correspond to individuals of European ancestry, the generalisability of our findings must be addressed with caution until confirmed in samples from other populations and ethnicities. In addition, analyses performed here are dependent on the statistical power of the original GWAS. Thus, null findings in our study do not necessarily indicate a lack of association. Also, we acknowledge that Mendelian randomisation methods assume no sample overlap between phenotypes. In this study, we were unable to fulfill this assumption as the UK Biobank cohort is included in summary data for PD and the volumes of all brain structures, accounting for 51% of the sample for the latter. Thus, excluding the UK Biobank cohort from our GWAS summary statistics would have resulted in a severe loss of statistical power, ultimately hindering our capacity to infer putative causal genetic effects. Mendelian randomisation findings in the present study must be addressed with caution until replicated using independent samples. Furthermore, the cohorts included in the present study do not have data available for the volume of the *substantia nigra* or the midbrain, which are brain structures severely damaged in PD. Nonetheless, we provide meaningful findings for the brainstem, which contains the midbrain and the *substantia nigra*.

In summary, we leveraged genome-wide association studies to delineate the genetic overlap between PD and the volume of intracranial and subcortical brain structures. Our findings suggest that genetic variants influencing larger intracranial and subcortical brain volumes are within genes whose dysfunction may impact the aetiology of PD. In particular, we provide evidence for shared genetic pathways involving chronic inflammation, the hypothalamic-pituitary-adrenal pathway, mitophagy, regulation of the SNARE complex, and the disruption of calcium-dependent, vesicle-trafficking, and autophagic pathways. Mendelian randomisation investigations suggest that larger intracranial and five subcortical brain volumes could influence the development of PD, independently of the potential causal genetic effects of ICV. Although the neurobiology of PD is complex and has not been fully elucidated, our findings contribute to improve our understanding of the aetiology of PD. Further exploration of the biological and genetic pathways observed in the present study has the potential to provide new research avenues, which in turn may prompt the development of new interventions to prevent or slow down disease onset and progression.

## Methods

### Parkinson’s disease GWAS data

We leveraged publicly available GWAS summary statistics for a PD meta-analysis that included ~37.7 K cases, ~18.6 K UK Biobank proxy-cases (having a first-degree relative with PD), and 1,417,791 controls, yielding a total sample size of 1,474,097. This dataset included samples of European ancestry from multiple cohorts, including the International Parkinson’s Disease Genomics Consortium (IPDG), 23andMe, and the UK Biobank. Briefly, the meta-analysis was performed using a fixed-effects model as implemented in METAL^66^. A detailed description of these GWAS summary statistics is available in the corresponding original publication^14^.

### Subcortical brain structures and intracranial volume GWAS data

GWAS summary statistics used in the present study included ICV and nine subcortical brain structures: the brainstem, caudate nucleus, hippocampus, putamen, pallidum, thalamus, ventral diencephalon, nucleus accumbens, and amygdala. Briefly, these GWAS summary statistics were derived from a meta-analysis of MRI scan data in ~70,000 European ancestry participants from international datasets, including the UK Biobank^67^ and The Adolescent Brain Cognitive Development (ABCD) cohorts^68^, and the ENIGMA^69^ and CHARGE consortia^70^. Specifically, intracranial and subcortical brain volumes were defined as the mean volume of both hemispheres (cm^3^), except for the brainstem, for which the total volume was used. Full details on phenotype definition and ENIGMA’s protocols for data adquisition and genotyping are available at the corresponding reference^8,71,72^. We obtained GWAS summary statistics from the ENGIMA-CHARGE consortium through the corresponding data application. GWAS for the UK Biobank and ABCD cohorts were performed using a linear mixed model accounting for genotyping array, sex, age, sex*age, age^2^, sex*age^2^, and the first 20 genetic principal components to adjust further for the potential effects arising from sex differences^73^, fluctuations in brain volumes due to age differences among individuals^74^, and population stratification. Subcortical brain volumes GWAS were adjusted for the effects of total ICV. Variants with a low minor allele frequency (<0.01) or a low-quality imputation score (<0.60) were excluded from the analysis.

### Genetic correlations

We estimated genetic correlations between PD and brain structures using pairwise LDSC^75^. We applied Bonferroni multiple testing correction to define statistical significance (i.e., 0.05 / 10 [number of brain structures] = 0.005). In addition, we estimated the genetic correlation between ICV and BMI^76^ to investigate potential genetic overlap.

### GWAS-pairwise

The GWAS-pairwise (GWAS-PW) method^77^ was used to identify specific segments of the genome influencing PD and at least one of the eight brain structures with a statistically significant genetic correlation, as described previously^78^. We conducted GWAS-PW analyses for PD and each of the eight brain structures, separately. Briefly, GWAS-PW splits the genome into 1703 independent segments. Then, for each region, GWAS-PW estimates the posterior probability of association (PPA) for four different models (i) the region is uniquely associated to PD, (ii) the region is uniquely associated to the volume of the brain structure, (iii) the region is influencing the aetiology of both phenotypes through the same genetic variants, and (iv) the region is involved in the aetiology of both phenotypes via different genetic variants^78,79^. For this analysis, we selected segments of the genome where model three (the region is influencing the aetiology of both phenotypes through the same genetic variants) had a PPA > 0.5, as this threshold has been used in previous studies using the GWAS-PW method^78,79^.

### Functional annotation

We used the FUMA online platform (v1.3.7)^80^ to perform functional annotation across the entire genome. Then, we extracted functional information for the precise genomic segments identified through GWAS-PW. The FUMA platform leverages MAGMA^81^ (v1.08) to perform gene-based association tests. We then selected significant genes after applying a Bonferroni multiple testing correction defined as *p* = 0.05 / [total number of genes for each subcortical brain structure].

### Genetic causal proportion

The LCV method leverages GWAS summary data to investigate whether a genetic correlation between two phenotypes (A and B) could be explained by horizontal pleiotropic effects or by a putative causal association (i.e., vertical pleiotropic effects)^59^. Specifically, the LCV method fits a latent variable *L* to mediate the genetic overlap between two phenotypes, assuming that *L* is causal for both^59^. This method compares the correlation between *L* and phenotype A, with the correlation between *L* and phenotype B to estimate the genetic causal proportion (GCP) parameter^59^. A detailed and illustrated description of this approach is available in previous studies^82,83^.

The GCP parameter indicates the proportion of a genetic correlation that could be explained by vertical pleiotropic effects on a scale ranging from −1 to 1. A GCP = 0 indicates the presence of horizontal pleiotropic effects, thus an absence of putative causal genetic effects^84,85^. Conversely, |GCP| = 1 suggests the full identification of vertical pleiotropic effects supporting a potential causal association^84,85^. A positive GCP estimate suggests vertical pleiotropic effects from phenotype A on phenotype B, whereas a negative GCP estimate suggests vertical pleiotropic effects from phenotype B on phenotype A^84,85^. Two of the main strengths of the LCV method include that it is less susceptible to bias due to horizontal pleiotropy, and it is robust to sample overlap^59^. In the present study, we performed LCV analyses for PD and the volume of brain structures with a statistically significant genetic correlation after Bonferroni multiple testing correction.

### Sensitivity analyses

#### Mendelian randomisation

The Mendelian randomisation method is frequently used in genetic epidemiological studies to investigate the potential causal effect of the genetic liability for a given phenotype on an outcome^86,87^. Mendelian randomisation is characterised by the use of genetic variants as instrumental variables. Genetic variants are robust instrumental variables to investigate putative causal genetic associations as alleles are randomly and independently segregated based on Mendel’s laws of segregation and independent assortment. In a Mendelian randomisation analysis, the effect sizes of the instrumental variables on the putative causal determinant or risk factor are compared against their effect on the outcome, which in turn may provide evidence for instrumental variables acting on the outcome via the putative risk factor (vertical pleiotropic effects)^85,88^. Furthermore, Mendelian randomisation methods rely on three core assumptions to draw conclusions regarding potential causal genetic effects. Among these, one of the essential assumptions is that vertical pleiotropy drives the association between exposure and outcome phenotypes, meaning that genetic variants are only associated with the outcome through the exposure and not through a third mediating variable. Also, genetic variants must not be confounded, and genetic variants must be strongly associated with the exposure of interest but not the outcome^89,90^.

In the present study, Mendelian randomisation analyses were performed using R-4.0.2^91,92^ and three main libraries. The MR-Base library includes two more libraries: MR Instruments and TwoSampleMR^93^. In particular, the MR-Base library enables the implementation of multiple Mendelian randomisation methods, which ultimately lead to the calculation of five causality estimates. In this study, we present findings for five Mendelian randomisation methods used to assess putative causal associations between subcortical brain structures with a significant genetic correlation after Bonferroni multiple testing correction with PD. These methods included inverse variance weighted (IVW), which estimates the magnitude of the putative causal genetic effect as the average of the instrumental variable - outcome effect divided by the instrumental variable exposure effect for all instrumental variables associated with the and weighted by the inverse variance of the instrumental variable - outcome effect^94^; Mendelian randomisation Egger (MR-Egger) performs a weighted linear regression where the intercept is free to vary and the slope represents the causal genetic effect of the exposure on the outcome free from horizontal pleiotropy^94,95^; the weighted median method that adjusts for as many as half of the instrumental variables with a horizontal pleiotropic effect; simple mode Mendelian randomisation, which clusters instrumental variables based on the similarity of the potential causal effect estimates^94^; and the weighted mode that weighs the number of instrumental variables within each cluster via the inverse variance of the effect on the outcome^94^.

As a sensitivity analysis, we performed Mendelian randomisation analyses using Generalised Summary-data-based Mendelian randomisation (GSMR), a Genome-wide Complex Trait Analysis (GCTA) package tool. Similarly to IVW in MR-BASE, GSMR leverages full GWAS summary statistics, clumps instrumental variables through colinearity thresholding based on a linkage disequilibrium reference panel, which in the present study was LD derived from ~5000 UK biobank individuals, and additionally adjusts for heterogeneous SNP-outliers through HEIDI-filtering. Thus, providing a single MR estimate, adjusted for pleiotropic effects^89^.

We further tested significant causally associated subcortical brain structures in a multivariate model adding the effects of ICV using Multivariable Mendelian randomisation (MVMR). This method is an extension to Mendelian randomisation methods that leverage instrumental variables associated with multiple and potentially related exposures to estimate the effect of each exposure on a single outcome (Sanderson, 2021). In addition, we used the MR-PRESSO (Mendelian Randomization Pleiotropy RESidual Sum and Outlier) method to evaluate the potential horizontal pleiotropic effects of ICV^96^. We removed outlier SNPs identified through MR-PRESSO and performed GCTA-GSMR to assess the ICV’s causal influence on PD.

#### Alzheimer’s disease GWAS data

We leveraged publicly available GWAS summary statistics for AD from the GWAS catalogue (https://www.ebi.ac.uk/gwas/) under accession number GCST90027158. Briefly, these genome-wide summary statistics include 111,326 clinically diagnosed or proxy patients and 677,663 controls from a number of international cohorts, such as the UK Biobank, FinnGen, and the Rotterdam study^64^. A detailed description of these GWAS summary statistics is available in the corresponding publication^64^.

#### Genetic correlations and GWAS-PW analyses for Alzheimer’s disease and intracranial and subcortical brain volumes

We estimated genetic correlations between AD and brain structures using pairwise LDSC^75^. We applied Bonferroni multiple testing correction to define statistical significance (i.e., 0.05 / 10 [number of brain structures] = 0.005). Furthermore, we conducted GWAS-PW analyses for AD and all of the brain structures, separately. For this GWAS-PW analysis, we followed the same approach as mentioned previously for PD. Thus, we selected segments of the genome where model three (the region is influencing the aetiology of both phenotypes through the same genetic variants) had a PPA > 0.5, as this threshold has been used in previous studies using the GWAS-PW method^78,79^.

### Reporting summary

Further information on research design is available in the Nature Research Reporting Summary linked to this article.

## Supplementary information

Supplementary Material

Reporting Summary

## Data Availability

Full summary-level data for PD, including the 23andMe cohort, are available upon request through a Data Transfer Agreement and the appropriate application procedure (https://research.23andme.com/dataset-access/).

## Code availability

Code to perform analyses in the present study is available in the corresponding repositories as mentioned in the corresponding publications. GWAS-PW: https://github.com/bulik/ldsc; LDSC: https://github.com/bulik/ldsc.
