# Supplementary material for: Shared molecular genetic factors influence subcortical brain morphometry and Parkinson’s disease risk
Source: NPJ Parkinsons Dis. 2023 May 10;9:73. doi: 10.1038/s41531-023-00515-y (PMC10172359; doi:10.1038/s41531-023-00515-y)
Supplement: Supplementary file 1 — Supplementary Material [file 41531_2023_515_MOESM1_ESM.pdf]

Shared molecular genetic factors influence subcortical brain morphometry and Parkinson's disease risk

Luis M. García-Marín[1,2,3], Paula Reyes-Pérez[3], Santiago Diaz-Torres[1,2], Alejandra Medina-Rivera[3], Nicholas G. Martin[1], Brittany L. Mitchell[1,2,*], Miguel E. Rentería[1,2,*]

1. Mental Health and Neuroscience Program, QIMR Berghofer Medical Research Institute, Brisbane, QLD, Australia
2. School of Biomedical Sciences, Faculty of Medicine, The University of Queensland, Brisbane, QLD, Australia
3. Laboratorio Internacional de Investigación del Genoma Humano, Universidad Nacional Autónoma de México, Juriquilla, Querétaro, México

*These authors jointly supervised this study.
Correspondence: Luis M. García-Marín (luis.garciamarin@qimrberghofer.edu.au)

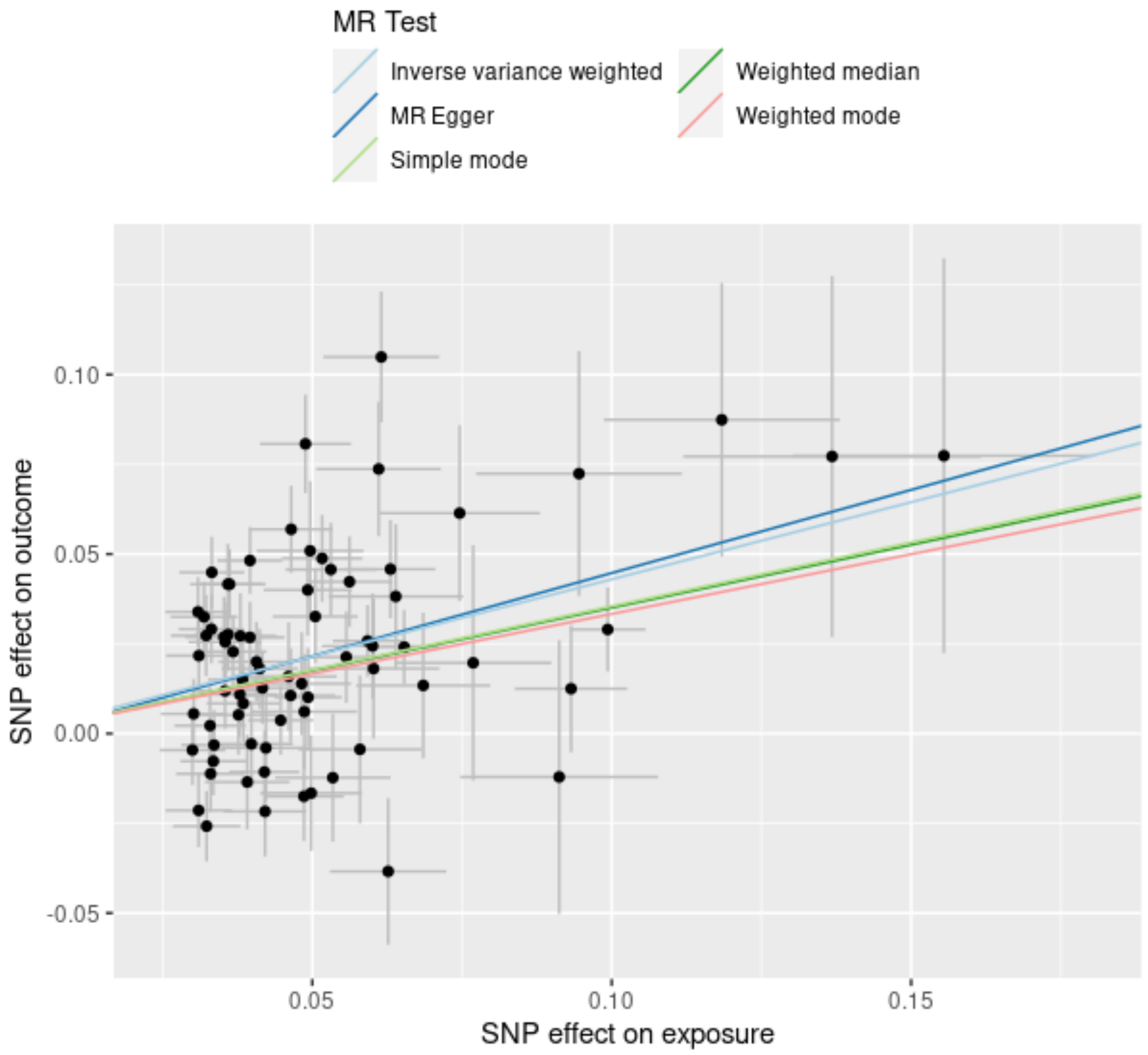

Supplementary Figure 1. Scatter plot showing Mendelian randomization effect estimates of intracranial volume (ICV) on Parkinson's disease (PD). Each SNP of the outcome (PD) is plotted against the SNP of the exposure (ICV) for different methods, including inverse variance weighted, MR Egger, simple mode, weighted median, and weighted mode.

MR	OR	-CI	+CI	P
MR Egger	1.47	1.04	2.06	2.97e-02
Weighted median	1.42	1.27	1.59	1.11e-09
IVW	1.52	1.37	1.68	2.97e-15
Simple mode	1.38	1.03	1.84	2.93e-02
Weighted mode	1.37	1.15	1.63	4.79e-04
GSMR	1.71	1.56	1.87	2.33e-32

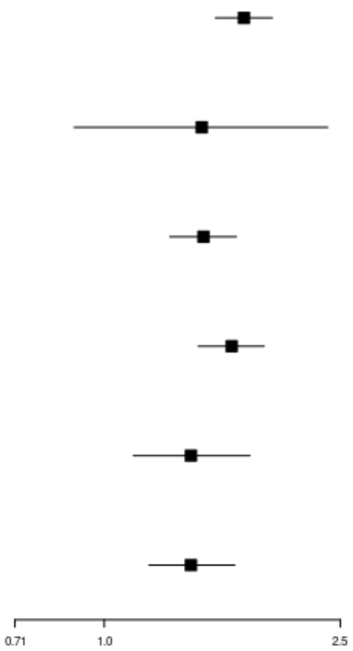

Supplementary Figure 2. Forest plot describing the odds ratio for each Mendelian randomization method with a 95% confidence interval for the causal effect of intracranial volume on Parkinson's disease risk.

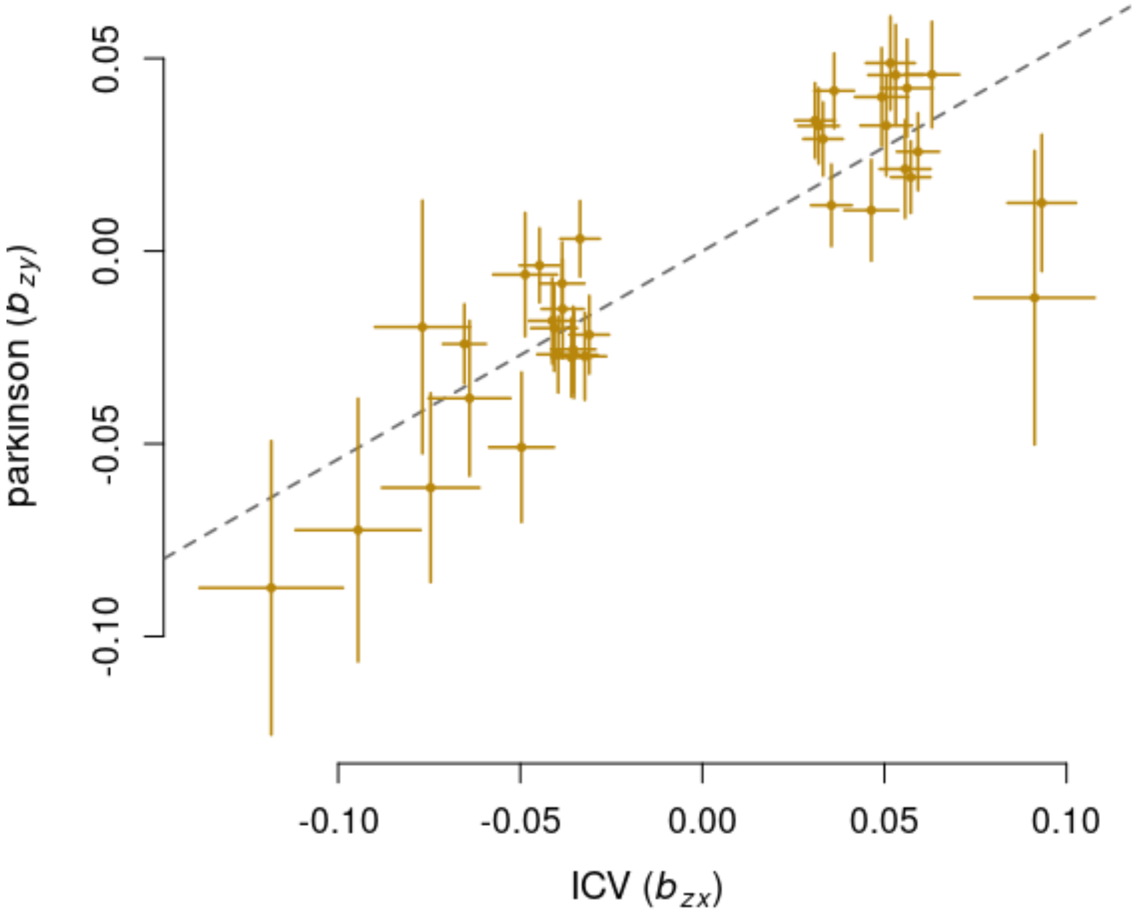

Supplementary Figure 3. Effect of intracranial volume (ICV) on Parkinson's disease risk based on Mendelian randomization results for GSMR.

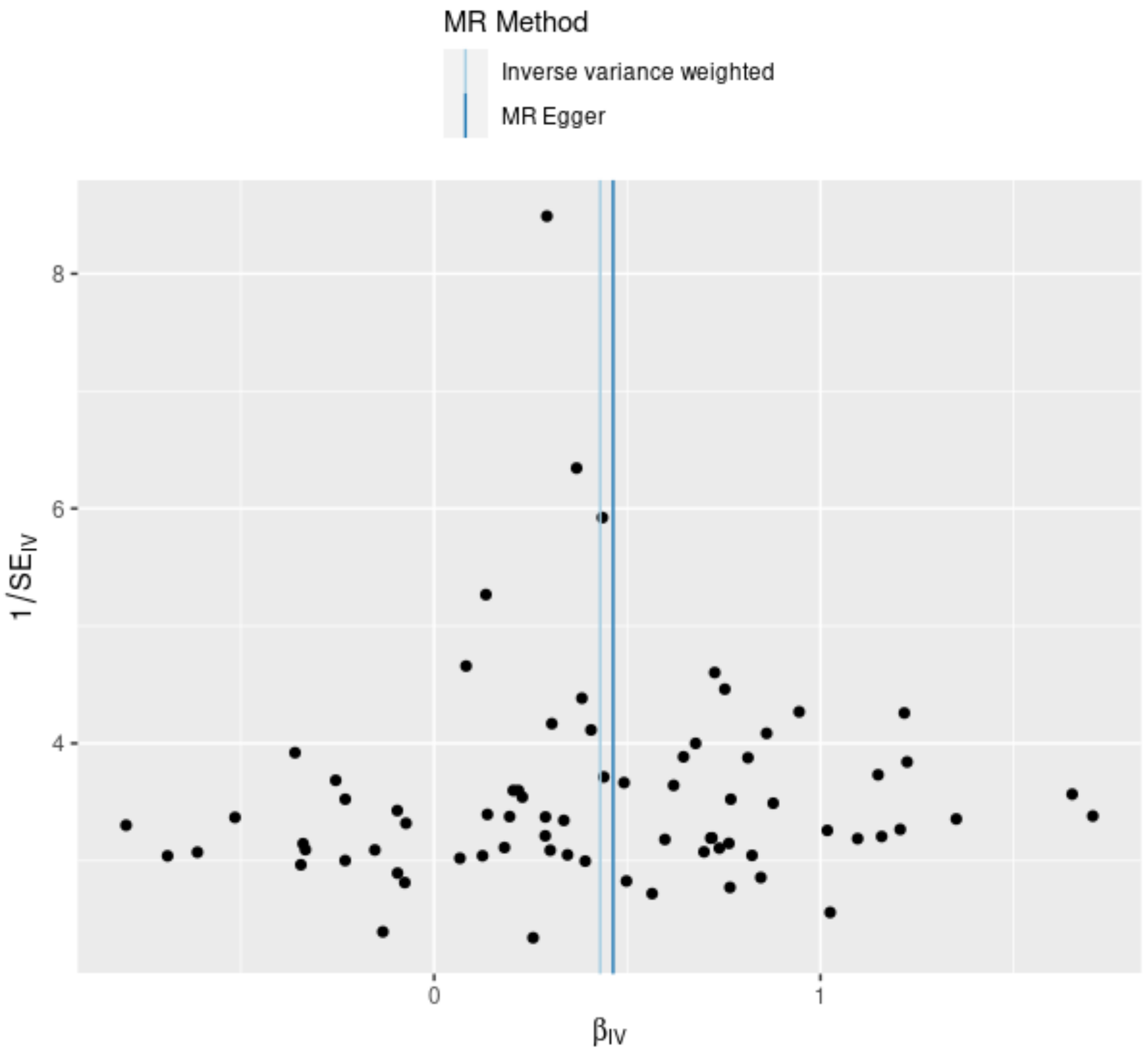

Supplementary Figure 4. Funnel plot showing intracranial volume (ICV) Mendelian randomisation effect estimates against the inverse standard error. Overall, the symmetry of the plot indicates that there is no unbalanced horizontal pleiotropy affecting the estimates. Genetic variants shown in this figure were used to investigate the causal association between ICV and Parkinson's disease.

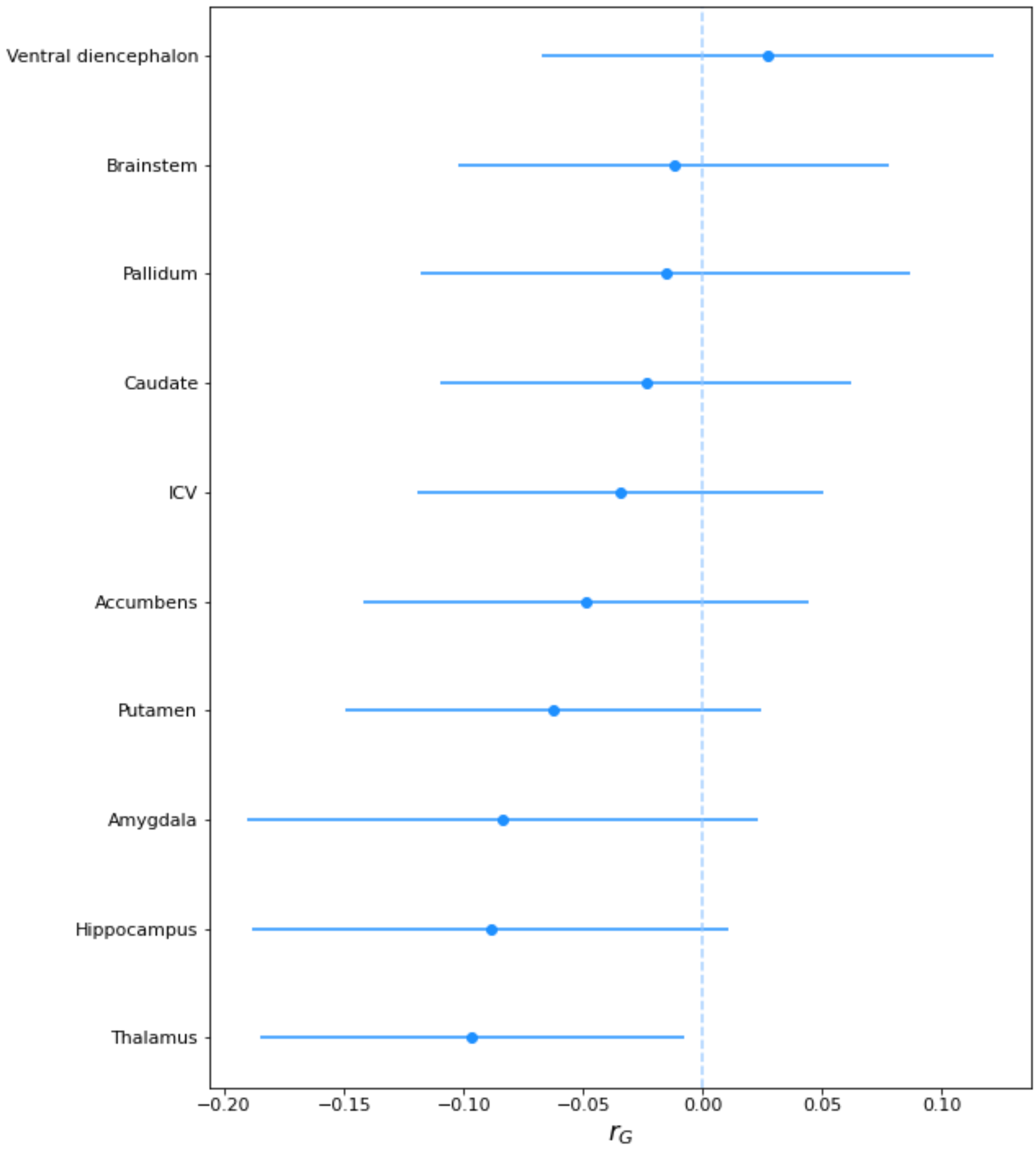

Supplementary Figure 5. Linkage disequilibrium score regression (LDSC) estimates of the genetic correlation (rG) with 95% confidence intervals between Alzheimer's disease and the volume of ten brain structures adjusted for intracranial volume. No statistically significant genetic correlations were observed after multiple testing correction. ICV = Intracranial volume.

This file shows supplementary tables for the manuscript: Shared molecular genetic factors influence subcortical brain morphometry and Pa

1. Genomic segments with genetic variants influencing PD and the volume of one brain structure
2. PD_Accubens : Statistically significant MAGMA results for the accumbens
3. PD_Thalamus: Statistically significant MAGMA results for the thalamus
4. PD_Putamen: Statistically significant MAGMA results for the putamen
5. PD_Caudate: Statistically significant MAGMA results for the caudate
6. PD_Brainstem: Statistically significant MAGMA results for the brainstem
7. PD:Pallidum: Statistically significant MAGMA results for the pallidum
8. PD_ICV: Statistically significant MAGMA results for the intracranial volume
9. PD_ventralDC: Statistically significant MAGMA results for the ventral diencephalon
10. Latent causal variable results
11. GSMR: Generalised Summary-data-based Mendelian Randomisation between Parkinson's disease and subcortical brain structures
12. MVMR: Multivariable Mendelian randomisation between Parkinson's disease and subcortical brain structures
13. Genetic correlation results between Alzheimer's disease and intracranial and subcortical brain volumes
14. Genomic segments with genetic variants influencing AD and the volume of one brain structure

rkinson’s disease risk

Supplementary Table 1. GWAS-pairwise results showing 210 genomic segments influencing				
phenotype	chr	start_basepair	stop_basepair	PPA model 3
ventralDC	1	43759733	44968965	0.540728
ventralDC	1	66939404	68476806	0.859611
ventralDC	1	2.42E+08	2.44E+08	0.549045
ventralDC	2	31550628	33363359	0.986098
ventralDC	2	33363777	35344684	0.799378
ventralDC	2	73176941	75630048	0.942635
ventralDC	2	1.67E+08	1.70E+08	0.503207
ventralDC	2	2.03E+08	2.06E+08	0.866099
ventralDC	3	47727379	49314960	0.714502
ventralDC	3	51836452	54081158	0.829679
ventralDC	3	1.51E+08	1.53E+08	0.983908
ventralDC	5	63968304	65910972	0.969835
ventralDC	5	1.36E+08	1.39E+08	0.881187
ventralDC	8	1.16E+08	1.20E+08	0.669945
ventralDC	8	1.30E+08	1.32E+08	0.986731
ventralDC	8	1.41E+08	1.43E+08	0.625721
ventralDC	9	94167810	96670716	0.895748
ventralDC	11	1213806	3662892	0.993141
ventralDC	13	1.10E+08	1.11E+08	0.986438
ventralDC	14	29972554	32383073	0.919505
ventralDC	14	94325812	95750857	0.607423
ventralDC	16	27446054	29029254	0.952109
ventralDC	17	15020965	16411387	0.735447
ventralDC	17	27334838	29785128	0.93321
ventralDC	17	39901356	41772005	0.995884
ventralDC	17	43056905	45875093	0.5841
ventralDC	19	6685945	8336797	0.98179
ventralDC	22	19912358	22357271	0.520543
Pallidum	1	43759733	44968965	0.740308
Pallidum	2	31550628	33363359	0.995751
Pallidum	2	33363777	35343877	0.672954
Pallidum	2	1.02E+08	1.03E+08	0.717326
Pallidum	4	1.01E+08	1.03E+08	0.989181
Pallidum	5	63968304	65910972	0.814187
Pallidum	7	98717552	1.00E+08	0.98485
Pallidum	8	8092025	9154609	0.612047
Pallidum	8	11279221	13491594	0.831339
Pallidum	8	37384185	38938628	0.914384
Pallidum	10	1.16E+08	1.20E+08	0.62314
Pallidum	11	1244197	3659768	0.994598
Pallidum	11	1.19E+08	1.21E+08	0.639818
Pallidum	12	53040411	54777633	0.526156
Pallidum	17	15020965	16411387	0.729846
Pallidum	17	39901356	41772005	0.969228
Pallidum	17	43056905	45874715	0.690484
Pallidum	19	40171266	40982866	0.981376
Pallidum	20	24717724	25344089	0.509994
Pallidum	20	25344231	31613480	0.992524
Pallidum	22	49825733	51178090	0.607567
ICV	1	1.53E+08	1.55E+08	0.610047

ICV	1	2.05E+08	2.06E+08	0.809014
ICV	1	2.42E+08	2.44E+08	0.987375
ICV	2	23342140	24686441	0.804993
ICV	2	31550628	33363359	0.67981
ICV	2	88304146	89134113	0.900222
ICV	2	1.02E+08	1.03E+08	0.734011
ICV	3	41065413	42538180	0.678754
ICV	3	42541598	45162483	0.756961
ICV	3	47727379	49314960	0.891806
ICV	3	49317338	51828635	0.768971
ICV	3	1.41E+08	1.43E+08	0.932782
ICV	4	17383953	18841225	0.976374
ICV	5	1.39E+08	1.41E+08	0.565996
ICV	5	1.76E+08	1.77E+08	0.837261
ICV	6	35456827	37572144	0.78893
ICV	6	1.57E+08	1.58E+08	0.543348
ICV	7	5854526	6573087	0.758912
ICV	7	6573917	7808270	0.667826
ICV	7	98717233	1.00E+08	0.746823
ICV	8	95006099	96534540	0.517797
ICV	8	1.30E+08	1.32E+08	0.984266
ICV	8	1.41E+08	1.43E+08	0.581253
ICV	9	94167810	96670716	0.515488
ICV	10	19718264	22771609	0.693461
ICV	10	89154747	91011692	0.601873
ICV	10	1.34E+08	1.35E+08	0.56091
ICV	11	1244197	3659768	0.983633
ICV	12	49001866	51775225	0.859624
ICV	12	53040411	54777633	0.633272
ICV	13	78808914	80583456	0.874458
ICV	14	59448808	61677103	0.832383
ICV	15	73628714	76398392	0.604902
ICV	16	27446054	29029254	0.945665
ICV	17	15020965	16411387	0.917648
ICV	17	27334838	29784431	0.582005
ICV	17	41772813	43055718	0.853805
ICV	17	43056905	45874715	0.926905
ICV	17	59314313	61545486	0.531079
ICV	19	3019660	4346348	0.895168
ICV	19	6685945	8336410	0.971645
ICV	19	40171266	40982866	0.603845
ICV	20	24717724	25344089	0.867869
ICV	20	47203165	49238645	0.987981
ICV	22	19912358	22357271	0.919271
Accumbens	1	97886171	99800131	0.66827
Accumbens	2	31550628	33363359	0.977423
Accumbens	2	2.03E+08	2.06E+08	0.914447
Accumbens	3	49317338	51828635	0.797307
Accumbens	4	17383953	18841427	0.738279
Accumbens	4	1.01E+08	1.03E+08	0.999871
Accumbens	4	1.03E+08	1.05E+08	0.985203
Accumbens	7	98717552	1.00E+08	0.971454

Accumbens	12	46025200	47714786	0.541597
Accumbens	12	53040411	54777633	0.551294
Accumbens	16	27446054	29029254	0.960357
Accumbens	19	40171266	40982866	0.904481
Brainstem	1	66939404	68476806	0.820117
Brainstem	2	23342140	24686441	0.752755
Brainstem	2	26894985	28597624	0.511688
Brainstem	2	31550628	33363359	0.988695
Brainstem	2	33363777	35343877	0.806692
Brainstem	2	73197693	75630048	0.638125
Brainstem	2	88296268	89135764	0.892383
Brainstem	2	1.02E+08	1.03E+08	0.506175
Brainstem	3	49317338	51828635	0.730549
Brainstem	3	51836452	54081158	0.992212
Brainstem	3	1.51E+08	1.53E+08	0.924368
Brainstem	4	1.28E+08	1.29E+08	0.908084
Brainstem	4	1.29E+08	1.31E+08	0.929409
Brainstem	5	63968304	65910972	0.942811
Brainstem	5	1.36E+08	1.39E+08	0.672406
Brainstem	5	1.57E+08	1.59E+08	0.534984
Brainstem	7	22508611	23467942	0.556656
Brainstem	7	98717552	1.00E+08	0.922376
Brainstem	7	1.29E+08	1.30E+08	0.98393
Brainstem	7	1.48E+08	1.50E+08	0.510088
Brainstem	8	8092025	9154609	0.784353
Brainstem	8	10463513	11278541	0.628984
Brainstem	8	21662306	22896108	0.85691
Brainstem	8	1.02E+08	1.04E+08	0.66637
Brainstem	8	1.16E+08	1.20E+08	0.838001
Brainstem	8	1.30E+08	1.32E+08	0.991133
Brainstem	9	1.29E+08	1.30E+08	0.532667
Brainstem	11	1244197	3659768	0.995929
Brainstem	12	53040411	54777633	0.56699
Brainstem	13	1.10E+08	1.11E+08	0.990856
Brainstem	14	29972554	32382628	0.803872
Brainstem	16	27446054	29029254	0.836258
Brainstem	17	27334838	29785128	0.875845
Brainstem	17	39901356	41772005	0.977596
Brainstem	17	43056905	45874715	0.980769
Brainstem	19	6685945	8336778	0.966194
Brainstem	20	2470305	3828150	0.80548
Brainstem	20	5478037	7081704	0.866759
Brainstem	20	31617328	32809249	0.978945
Thalamus	1	2.42E+08	2.44E+08	0.984313
Thalamus	2	23342140	24686441	0.793495
Thalamus	2	31550628	33363359	0.882056
Thalamus	2	1.67E+08	1.70E+08	0.973389
Thalamus	2	2.03E+08	2.06E+08	0.552231
Thalamus	3	1.51E+08	1.53E+08	0.952878
Thalamus	4	1.01E+08	1.03E+08	0.54817
Thalamus	4	1.03E+08	1.05E+08	0.513359
Thalamus	5	63968304	65910972	0.952443

Thalamus	5	1.36E+08	1.39E+08	0.75786
Thalamus	8	1.16E+08	1.20E+08	0.691023
Thalamus	8	1.30E+08	1.32E+08	0.99223
Thalamus	10	124381	742875	0.86913
Thalamus	11	1244197	3659768	0.998993
Thalamus	11	72286156	74412268	0.624292
Thalamus	13	1.10E+08	1.11E+08	0.980188
Thalamus	15	63216468	65101626	0.837697
Thalamus	16	1225628	2754260	0.778168
Thalamus	19	6685945	8336778	0.953162
Thalamus	19	40171266	40982866	0.988024
Thalamus	22	19912358	22357271	0.545972
Putamen	1	97886171	99800131	0.914585
Putamen	2	31550628	33363359	0.925208
Putamen	2	1.02E+08	1.03E+08	0.603686
Putamen	2	1.60E+08	1.62E+08	0.583631
Putamen	3	51836452	54081158	0.554135
Putamen	4	1.01E+08	1.03E+08	0.999833
Putamen	4	1.03E+08	1.05E+08	0.988304
Putamen	5	50163883	52134410	0.599093
Putamen	5	87391401	88890063	0.801583
Putamen	5	1.23E+08	1.24E+08	0.740452
Putamen	6	29609600	29716146	0.631914
Putamen	6	1.30E+08	1.31E+08	0.930052
Putamen	8	37384185	38938628	0.529324
Putamen	12	49001866	51775225	0.730748
Putamen	12	1.09E+08	1.10E+08	0.840583
Putamen	13	1.10E+08	1.11E+08	0.870282
Putamen	14	94325812	95750857	0.834908
Putamen	16	27446054	29029254	0.963168
Putamen	17	43056905	45874715	0.957608
Putamen	17	61545779	63145396	0.792615
Putamen	19	40171266	40982866	0.95664
Putamen	19	42132193	43848726	0.672052
Putamen	20	25344231	31613480	0.976469
Caudate	2	23342140	24686441	0.913613
Caudate	2	31550628	33363359	0.777791
Caudate	2	1.90E+08	1.92E+08	0.648763
Caudate	3	49317338	51828635	0.685339
Caudate	3	1.51E+08	1.53E+08	0.657321
Caudate	4	1.01E+08	1.03E+08	0.999205
Caudate	4	1.03E+08	1.05E+08	0.988228
Caudate	5	50163883	52134410	0.528061
Caudate	7	22508611	23467942	0.780983
Caudate	7	98717552	1.00E+08	0.660314
Caudate	10	1.01E+08	1.03E+08	0.983069
Caudate	12	3679554	4416304	0.755514
Caudate	12	1.18E+08	1.20E+08	0.537569
Caudate	14	91296860	93131795	0.611699
Caudate	15	61266132	63214206	0.556106
Caudate	16	27446054	29029254	0.969658
Caudate	16	52036960	53376141	0.908534

Caudate	16	71093625	72934341	0.781165
Caudate	19	40171266	40982866	0.959317
Caudate	20	25344231	31613480	0.977793
Caudate	21	41390179	43321426	0.873912

Supplementary Table 2. Statistically significant MAGMA results for the PD and the volume of the accumbens									
GENE	CHR	START	STOP	NSNPS	NPARAM	N	ZSTAT	P_PD	SYMBOL
ENSG0000(	2	3.2E+07	3.2E+07	300	17	1403266	1.5606	Non_Significant	MEMO1
ENSG0000(	2	3.2E+07	3.2E+07	382	20	1390615	1.6523	Non_Significant	DPY30
ENSG0000(	2	3.2E+07	3.2E+07	223	21	1374335	3.0298	Non_Significant	SPAST
ENSG0000(	2	3.2E+07	3.2E+07	142	17	1348274	2.7724	Non_Significant	SLC30A6
ENSG0000(	2	3.2E+07	3.2E+07	84	11	1389044	2.3152	Non_Significant	NLRC4
ENSG0000(	2	3.3E+07	3.3E+07	78	16	1394543	2.9663	Non_Significant	YIPF4
ENSG0000(	2	3.3E+07	3.3E+07	594	23	1344931	2.0583	Non_Significant	BIRC6
ENSG0000(	2	3.3E+07	3.3E+07	720	47	1410484	2.564	Non_Significant	TTC27
ENSG0000(	2	3.3E+07	3.4E+07	1895	78	1427064	1.1835	Non_Significant	LTBP1
ENSG0000(	2	2E+08	2E+08	149	25	1376459	1.6162	Non_Significant	FAM117B
ENSG0000(	2	2E+08	2E+08	152	11	1437648	0.82168	Non_Significant	ICA1L
ENSG0000(	2	2E+08	2E+08	258	12	1406689	0.88732	Non_Significant	WDR12
ENSG0000(	2	2E+08	2E+08	132	9	1401939	0.59327	Non_Significant	CARF
ENSG0000(	2	2E+08	2E+08	251	26	1402458	1.1703	Non_Significant	NBEAL1
ENSG0000(	2	2E+08	2E+08	117	14	1412815	1.5439	Non_Significant	CYP20A1
ENSG0000(	2	2E+08	2E+08	183	29	1358905	1.8923	Non_Significant	ABI2
ENSG0000(	2	2E+08	2E+08	207	26	1372996	1.8092	Non_Significant	RAPH1
ENSG0000(	3	4.9E+07	4.9E+07	76	11	1404855	3.7726	0.0000808	USP4
ENSG0000(	3	4.9E+07	4.9E+07	2	1	1474097	-0.21623	Non_Significant	GPX1
ENSG0000(	3	4.9E+07	4.9E+07	94	11	1412713	1.3185	Non_Significant	RHOA
ENSG0000(	3	4.9E+07	4.9E+07	8	3	1376079	1.327	Non_Significant	TCTA
ENSG0000(	3	4.9E+07	4.9E+07	10	2	1417267	-0.3079	Non_Significant	AMT
ENSG0000(	3	4.9E+07	4.9E+07	7	4	1474097	4.651	0.00000165	NICN1
ENSG0000(	3	5E+07	5E+07	111	13	1398377	1.2415	Non_Significant	DAG1
ENSG0000(	3	5E+07	5E+07	168	17	1393309	1.7253	Non_Significant	BSN
ENSG0000(	3	5E+07	5E+07	7	3	1393216	1.5502	Non_Significant	MST1
ENSG0000(	3	5E+07	5E+07	53	10	1349639	1.7609	Non_Significant	RNF123
ENSG0000(	3	5E+07	5E+07	4	2	1474097	4.2776	0.00000945	AMIGO3
ENSG0000(	3	5E+07	5E+07	4	2	1474097	4.2776	0.00000945	GMPPB
ENSG0000(	3	5E+07	5E+07	98	12	1359198	1.265	Non_Significant	IP6K1
ENSG0000(	3	5E+07	5E+07	16	6	1467285	2.7727	Non_Significant	CDHR4
ENSG0000(	3	5E+07	5E+07	1	1	1365107	1.0035	Non_Significant	FAM212A
ENSG0000(	3	5E+07	5E+07	8	2	1460206	1.3358	Non_Significant	UBA7

ENSG0000(	3	5E+07	5E+07	39	6	1451141	1.5746	Non_Significant	TRAIP
ENSG0000(	3	5E+07	5E+07	14	4	1458527	1.3218	Non_Significant	CAMKV
ENSG0000(	3	5E+07	5E+07	19	5	1432826	0.42102	Non_Significant	MST1R
ENSG0000(	3	5E+07	5E+07	11	3	1454281	0.82422	Non_Significant	CTD-2330K
ENSG0000(	3	5E+07	5E+07	21	9	1410807	2.5442	Non_Significant	MON1A
ENSG0000(	3	5E+07	5E+07	269	14	1421476	1.6464	Non_Significant	RBM6
ENSG0000(	3	5E+07	5E+07	29	6	1435051	2.327	Non_Significant	RBM5
ENSG0000(	3	5E+07	5E+07	54	11	1397046	1.7828	Non_Significant	SEMA3F
ENSG0000(	3	5E+07	5E+07	38	11	1374820	1.8999	Non_Significant	GNAI2
ENSG0000(	3	5E+07	5E+07	5	2	1184389	1.3704	Non_Significant	LSMEM2
ENSG0000(	3	5E+07	5E+07	4	2	1333090	0.48878	Non_Significant	IFRD2
ENSG0000(	3	5E+07	5E+07	6	4	1454361	-0.25618	Non_Significant	HYAL3
ENSG0000(	3	5E+07	5E+07	3	2	1436049	-0.3306	Non_Significant	NAT6
ENSG0000(	3	5E+07	5E+07	13	5	1427997	1.4699	Non_Significant	HYAL1
ENSG0000(	3	5E+07	5E+07	2	1	1471960	0.04138	Non_Significant	HYAL2
ENSG0000(	3	5E+07	5E+07	5	3	1472387	1.3784	Non_Significant	TUSC2
ENSG0000(	3	5E+07	5E+07	12	4	1286319	0.84532	Non_Significant	RASSF1
ENSG0000(	3	5E+07	5E+07	3	1	1474097	0.50513	Non_Significant	ZMYND10
ENSG0000(	3	5E+07	5E+07	1	1	1474097	1.4469	Non_Significant	NPRL2
ENSG0000(	3	5E+07	5E+07	6	3	1361571	1.0015	Non_Significant	CYB561D2
ENSG0000(	3	5E+07	5E+07	13	5	1250111	0.95781	Non_Significant	XXcos-LUC/
ENSG0000(	3	5E+07	5E+07	3	2	1285376	-0.47112	Non_Significant	TMEM115
ENSG0000(	3	5E+07	5.1E+07	275	19	1285472	0.89014	Non_Significant	CACNA2D2
ENSG0000(	3	5.1E+07	5.1E+07	16	6	1410083	0.70354	Non_Significant	C3orf18
ENSG0000(	3	5.1E+07	5.1E+07	17	4	1413901	-0.22446	Non_Significant	HEMK1
ENSG0000(	3	5.1E+07	5.1E+07	11	4	1215793	1.9176	Non_Significant	CISH
ENSG0000(	3	5.1E+07	5.1E+07	58	9	1298007	1.0788	Non_Significant	MAPKAPK3
ENSG0000(	3	5.1E+07	5.1E+07	6	1	1471960	-0.46177	Non_Significant	MANF
ENSG0000(	3	5.1E+07	5.1E+07	9	4	1385448	-0.98149	Non_Significant	RBM15B
ENSG0000(	4	1E+08	1E+08	30	9	1359603	0.85564	Non_Significant	DDIT4L
ENSG0000(	4	1E+08	1E+08	1765	91	1360987	2.2776	Non_Significant	BANK1
ENSG0000(	4	1E+08	1E+08	522	34	1357547	1.8529	Non_Significant	SLC39A8
ENSG0000(	4	1E+08	1E+08	243	18	1416064	-0.53996	Non_Significant	NFKB1
ENSG0000(	4	1E+08	1E+08	156	9	1375227	1.7663	Non_Significant	UBE2D3
ENSG0000(	4	1E+08	1E+08	35	4	1323152	1.6519	Non_Significant	CISD2

ENSG0000(	4	1E+08	1E+08	251	10	1361410	2.1118	Non_Significant	SLC9B1
ENSG0000(	4	1E+08	1E+08	85	22	1264979	1.3826	Non_Significant	SLC9B2
ENSG0000(	4	1E+08	1E+08	49	7	1380425	-0.03464	Non_Significant	BDH2

GENOMIC_SEGMENT_NUMBER	P_Accumbens	PD_GWAS_TOP_GENE
152	1.46283E-08	FALSE
152	1.46283E-08	FALSE
152	1.46283E-08	FALSE
152	1.46283E-08	FALSE
152	4.59571E-08	FALSE
152	7.41947E-10	FALSE
152	2.23776E-09	FALSE
152	1.46283E-08	FALSE
152	3.30812E-06	FALSE
252	1.19284E-07	FALSE
252	2.27344E-07	FALSE
252	3.02283E-08	FALSE
252	3.84792E-06	FALSE
252	1.38932E-07	FALSE
252	5.21649E-08	FALSE
252	3.02283E-08	FALSE
252	3.02283E-08	FALSE
311	7.01075E-06	FALSE
311	9.71787E-09	FALSE
311	1.3602E-07	FALSE
311	1.3602E-07	FALSE
311	9.1322E-08	FALSE
311	9.1322E-08	FALSE
311	1.98299E-06	FALSE
311	4.88366E-07	FALSE
311	9.1322E-08	FALSE
311	9.1322E-08	FALSE
311	1.3602E-07	FALSE
311	1.3602E-07	FALSE
311	1.3602E-07	FALSE
311	1.38465E-07	FALSE
311	1.38465E-07	FALSE
311	1.38465E-07	FALSE

311	1.3602E-07	FALSE
311	1.3602E-07	FALSE
311	2.8047E-07	FALSE
311	2.8047E-07	FALSE
311	9.71787E-09	FALSE
311	2.30275E-08	FALSE
311	1.3602E-07	FALSE
311	9.71787E-09	FALSE
311	1.81517E-08	FALSE
311	1.3602E-07	FALSE
311	9.71787E-09	FALSE
311	9.71787E-09	FALSE
311	9.1322E-08	FALSE
311	9.71787E-09	FALSE
311	9.71787E-09	FALSE
311	1.3602E-07	FALSE
311	1.3602E-07	FALSE
311	1.3602E-07	FALSE
311	1.3602E-07	FALSE
311	1.3602E-07	FALSE
311	1.3602E-07	FALSE
311	1.3602E-07	FALSE
311	1.3602E-07	FALSE
311	1.38465E-07	FALSE
311	1.38465E-07	FALSE
311	3.43353E-07	FALSE
311	3.43353E-07	FALSE
311	3.43353E-07	FALSE
311	3.43353E-07	FALSE
465	5.1768E-36	FALSE
465	6.20993E-23	FALSE
465	5.1768E-36	FALSE
466	1.57689E-19	FALSE
466	3.95552E-20	FALSE
466	3.95552E-20	FALSE

466	3.95552E-20	FALSE
466	3.95552E-20	FALSE
466	3.95552E-20	FALSE

**SIGNIFICANT_IN_BOTH_AND_IN_SEGMENT_OF_INTEREST**
FALSE
FALSE
FALSE
FALSE
FALSE
FALSE
FALSE
FALSE
FALSE
FALSE
FALSE
FALSE
FALSE
FALSE
FALSE
FALSE
FALSE
TRUE
FALSE
FALSE
FALSE
FALSE
TRUE
FALSE
FALSE
FALSE
FALSE
TRUE
TRUE
FALSE
FALSE
FALSE
FALSE

FALSE
FALSE
FALSE
FALSE
FALSE
FALSE
FALSE
FALSE
FALSE
FALSE
FALSE
FALSE
FALSE
FALSE
FALSE
FALSE
FALSE
FALSE
FALSE
FALSE
FALSE
FALSE
FALSE
FALSE
FALSE
FALSE
FALSE
FALSE
FALSE
FALSE
FALSE
FALSE
FALSE
FALSE
FALSE

FALSE
FALSE
FALSE

Supplementary Table 3. Statistically significant MAGMA results for the PD and the volume of the thalamus										
GENE	CHR	START	STOP	NSNPS	NPARAM	N	ZSTAT	P_PD	SYMBOL	_SEGMENT
ENSG0000	1	2.4E+08	2.4E+08	204	22	1180065	0.95562	Non_Significant	CEP170	129
ENSG0000	1	2.4E+08	2.4E+08	429	36	1396738	2.2611	Non_Significant	SDCCAG8	129
ENSG0000	1	2.4E+08	2.4E+08	524	23	1313904	4.6686	0.00000152	AKT3	129
ENSG0000	5	6.4E+07	6.4E+07	74	8	1356729	1.1372	Non_Significant	SREK1IP1	560
ENSG0000	5	6.4E+07	6.4E+07	452	23	1410242	1.1395	Non_Significant	CWC27	560
ENSG0000	5	6.4E+07	6.5E+07	750	42	1421304	1.2298	Non_Significant	ADAMTS6	560
ENSG0000	5	6.5E+07	6.5E+07	146	12	1427851	2.1066	Non_Significant	CENPK	560
ENSG0000	5	6.5E+07	6.5E+07	67	8	1450309	1.9521	Non_Significant	PPWD1	560
ENSG0000	5	6.5E+07	6.5E+07	113	11	1420773	2.205	Non_Significant	TRIM23	560
ENSG0000	5	6.5E+07	6.5E+07	117	10	1447649	1.8195	Non_Significant	TRAPPC13	560
ENSG0000	5	6.5E+07	6.5E+07	16	4	1469855	2.1023	Non_Significant	CTC-534A2	560
ENSG0000	5	6.5E+07	6.5E+07	98	16	1375807	1.7663	Non_Significant	SGTB	560
ENSG0000	5	6.5E+07	6.5E+07	421	42	1408936	0.52104	Non_Significant	NLN	560
ENSG0000	5	6.5E+07	6.5E+07	183	22	1349411	1.6989	Non_Significant	ERBB2IP	560
ENSG0000	8	1.3E+08	1.3E+08	279	31	1404337	4.5825	0.0000023	FAM49B	926
ENSG0000	11	1295601	1330884	117	24	1374890	1.3043	Non_Significant	TOLLIP	1096
ENSG0000	11	1411129	1483919	317	31	1304150	2.4386	Non_Significant	BRSK2	1096
ENSG0000	11	1490687	1522477	101	10	1399639	3.1667	0.00077086	MOB2	1096
ENSG0000	11	1575274	1593150	60	7	1432984	3.0413	0.0011779	DUSP8	1096
ENSG0000	11	7.3E+07	7.3E+07	156	14	1366143	2.2509	Non_Significant	ARHGEF17	1135
ENSG0000	11	7.3E+07	7.3E+07	61	14	1351343	0.80238	Non_Significant	PLEKHB1	1135
ENSG0000	11	7.3E+07	7.3E+07	239	27	1360380	0.52486	Non_Significant	RAB6A	1135
ENSG0000	11	7.4E+07	7.4E+07	9	4	1396665	-0.05158	Non_Significant	COA4	1135
ENSG0000	11	7.4E+07	7.4E+07	143	17	1379214	0.09329	Non_Significant	PAAF1	1135
ENSG0000	11	7.4E+07	7.4E+07	75	14	1414906	1.3026	Non_Significant	DNAJB13	1135
ENSG0000	11	7.4E+07	7.4E+07	14	2	1466159	0.83344	Non_Significant	UCP2	1135
ENSG0000	11	7.4E+07	7.4E+07	21	6	1352416	-0.74127	Non_Significant	UCP3	1135
ENSG0000	11	7.4E+07	7.4E+07	225	24	1390667	1.371	Non_Significant	C2CD3	1135
ENSG0000	11	7.4E+07	7.4E+07	163	11	1406628	2.0476	Non_Significant	PPME1	1135
ENSG0000	11	7.4E+07	7.4E+07	179	17	1426887	0.89488	Non_Significant	P4HA3	1135
ENSG0000	11	7.4E+07	7.4E+07	5	2	1474097	0.85195	Non_Significant	LIPT2	1135
ENSG0000	11	7.4E+07	7.4E+07	537	29	1432338	-0.21143	Non_Significant	POLD3	1135
ENSG0000	11	7.4E+07	7.4E+07	86	22	1383974	0.18737	Non_Significant	CHRDL2	1135

ENSG0000(	15	6.3E+07	6.3E+07	100	14	1406005	2.8071	Non_Significant	TPM1	1407
ENSG0000(	15	6.3E+07	6.3E+07	51	6	1414363	1.9893	Non_Significant	LACTB	1407
ENSG0000(	15	6.3E+07	6.3E+07	110	14	1407186	2.1486	Non_Significant	RPS27L	1407
ENSG0000(	15	6.3E+07	6.4E+07	183	26	1392960	2.1824	Non_Significant	RAB8B	1407
ENSG0000(	15	6.4E+07	6.4E+07	81	17	1380368	2.0441	Non_Significant	APH1B	1407
ENSG0000(	15	6.4E+07	6.4E+07	147	25	1412778	1.1903	Non_Significant	CA12	1407
ENSG0000(	15	6.4E+07	6.4E+07	9	4	1397541	2.1723	Non_Significant	FBXL22	1407

P_Accumbens	PD_GWAS_TOP_GENE	SIGNIFICANT_IN_BOTH_AND_IN_SEGMENT_OF_INTEREST
1.42704E-10	FALSE	FALSE
5.4039E-11	FALSE	FALSE
5.4039E-11	FALSE	TRUE
1.39589E-14	FALSE	FALSE
1.39589E-14	FALSE	FALSE
6.38689E-13	FALSE	FALSE
1.10308E-14	FALSE	FALSE
1.10308E-14	FALSE	FALSE
1.39073E-14	FALSE	FALSE
1.10308E-14	FALSE	FALSE
1.10308E-14	FALSE	FALSE
1.10308E-14	FALSE	FALSE
1.39589E-14	FALSE	FALSE
6.25267E-09	FALSE	FALSE
6.41402E-12	FALSE	TRUE
8.70512E-11	FALSE	FALSE
8.70512E-11	FALSE	FALSE
8.70512E-11	FALSE	TRUE
1.91534E-09	FALSE	TRUE
2.67267E-08	FALSE	FALSE
7.56088E-08	FALSE	FALSE
2.67267E-08	FALSE	FALSE
5.93924E-06	FALSE	FALSE
2.67267E-08	FALSE	FALSE
2.67267E-08	FALSE	FALSE
2.67267E-08	FALSE	FALSE
5.93924E-06	FALSE	FALSE
5.93924E-06	FALSE	FALSE
5.93924E-06	FALSE	FALSE
5.93924E-06	FALSE	FALSE
5.93924E-06	FALSE	FALSE
5.93924E-06	FALSE	FALSE
5.93924E-06	FALSE	FALSE

5.25712E-10	FALSE	FALSE
1.22164E-10	FALSE	FALSE
1.22164E-10	FALSE	FALSE
1.22164E-10	FALSE	FALSE
1.67587E-10	FALSE	FALSE
4.12643E-09	FALSE	FALSE
1.67587E-10	FALSE	FALSE

Supplementary Table 4. Statistically significant MAGMA results for the PD and the volume of the putamen											
GENE	CHR	START	STOP	NSNPS	NPARAM	N	ZSTAT	P_PD	SYMBOL	_SEGMENT	_Accumber
ENSG0000(	4	1E+08	1E+08	30	9	1359603	0.85564	Non_Signif	DDIT4L	465	4.9E-15
ENSG0000(	4	1E+08	1E+08	1765	91	1360987	2.2776	Non_Signif	BANK1	465	2.8E-12
ENSG0000(	4	1E+08	1E+08	522	34	1357547	1.8529	Non_Signif	SLC39A8	465	4.9E-15
ENSG0000(	4	1E+08	1E+08	243	18	1416064	-0.53996	Non_Signif	NFKB1	466	5.1E-08
ENSG0000(	4	1E+08	1E+08	156	9	1375227	1.7663	Non_Signif	UBE2D3	466	3.9E-08
ENSG0000(	4	1E+08	1E+08	35	4	1323152	1.6519	Non_Signif	CISD2	466	3.9E-08
ENSG0000(	4	1E+08	1E+08	251	10	1361410	2.1118	Non_Signif	SLC9B1	466	3.9E-08
ENSG0000(	4	1E+08	1E+08	85	22	1264979	1.3826	Non_Signif	SLC9B2	466	3.9E-08
ENSG0000(	4	1E+08	1E+08	49	7	1380425	-0.03464	Non_Signif	BDH2	466	3.9E-08
ENSG0000(	5	5.1E+07	5.1E+07	21	7	1423711	0.64442	Non_Signif	ISL1	550	2.4E-10
ENSG0000(	5	5.1E+07	5.1E+07	229	13	1427619	-0.46964	Non_Signif	CTD-2203A	550	2.3E-10
ENSG0000(	5	8.7E+07	8.8E+07	129	19	1346690	3.6586	0.00013	TMEM161E	572	3.1E-18
ENSG0000(	6	1.3E+08	1.3E+08	414	44	1438384	0.48479	Non_Signif	ARHGAP18	716	1.3E-08
ENSG0000(	6	1.3E+08	1.3E+08	332	25	1420100	0.48954	Non_Signif	L3MBTL3	716	1.3E-08
ENSG0000(	13	1.1E+08	1.1E+08	86	13	1457049	3.1481	Non_Signif	IRS2	1319	7.6E-10
ENSG0000(	16	2.8E+07	2.8E+07	127	10	1298179	0.99045	Non_Signif	XPO6	1451	1.5E-08
ENSG0000(	16	2.8E+07	2.8E+07	46	14	1203651	1.4279	Non_Signif	SBK1	1451	8E-09
ENSG0000(	16	2.8E+07	2.9E+07	14	5	564629	1.335	Non_Signif	CLN3	1451	4.1E-11
ENSG0000(	16	2.8E+07	2.9E+07	14	5	564629	1.335	Non_Signif	CLN3	1451	1.6E-09
ENSG0000(	16	2.8E+07	2.9E+07	17	7	639090	1.4032	Non_Signif	CLN3	1451	4.1E-11
ENSG0000(	16	2.8E+07	2.9E+07	17	7	639090	1.4032	Non_Signif	CLN3	1451	1.6E-09
ENSG0000(	16	2.9E+07	2.9E+07	5	3	581146	0.19996	Non_Signif	APOBR	1451	1.6E-09
ENSG0000(	16	2.9E+07	2.9E+07	15	4	1128751	-0.82297	Non_Signif	IL27	1451	4.1E-11
ENSG0000(	16	2.9E+07	2.9E+07	7	2	1207864	1.3701	Non_Signif	NUPR1	1451	4.1E-11
ENSG0000(	16	2.9E+07	2.9E+07	78	17	1007931	1.6601	Non_Signif	CCDC101	1451	3.1E-09
ENSG0000(	16	2.9E+07	2.9E+07	18	8	956879	1.0048	Non_Signif	SULT1A2	1451	4.1E-11
ENSG0000(	16	2.9E+07	2.9E+07	40	11	512811	3.2711	0.00054	SULT1A1	1451	4.1E-11
ENSG0000(	16	2.9E+07	2.9E+07	1	1	15976	1.4837	Non_Signif	EIF3C	1451	4.1E-11
ENSG0000(	16	2.9E+07	2.9E+07	27	3	1298772	5.2122	9.3E-08	ATXN2L	1451	4.6E-11
ENSG0000(	16	2.9E+07	2.9E+07	6	1	1339784	4.8751	5.4E-07	TUFM	1451	4.1E-11
ENSG0000(	16	2.9E+07	2.9E+07	47	4	1260457	5.1361	1.4E-07	SH2B1	1451	4.1E-11
ENSG0000(	16	2.9E+07	2.9E+07	27	6	1237705	5.5829	1.2E-08	ATP2A1	1451	4.6E-11
ENSG0000(	16	2.9E+07	2.9E+07	40	11	1090570	5.4499	2.5E-08	RABEP2	1451	1.7E-10

ENSG0000(	16	2.9E+07	2.9E+07	10	5	998004	4.5274	3E-06	CD19	1451	1.7E-10
ENSG0000(	16	2.9E+07	2.9E+07	28	8	1128945	3.7394	9.2E-05	NFATC2IP	1451	4.1E-11
ENSG0000(	16	2.9E+07	2.9E+07	23	7	1087907	5.1938	1E-07	SPNS1	1451	4.6E-11
ENSG0000(	16	2.9E+07	2.9E+07	11	5	991708	3.7524	8.8E-05	LAT	1451	4.1E-11
ENSG0000(	17	4.3E+07	4.3E+07	136	16	1430326	0.92897	Non_Signif	NMT1	1509	4.1E-08
ENSG0000(	17	4.3E+07	4.3E+07	12	5	1302564	3.8316	6.4E-05	HEXIM2	1509	1.8E-07
ENSG0000(	17	4.3E+07	4.3E+07	50	8	1430881	1.8861	Non_Signif	FMNL1	1509	4.1E-08
ENSG0000(	17	4.3E+07	4.3E+07	24	4	1474097	2.0315	Non_Signif	SPATA32	1509	7.3E-05
ENSG0000(	17	4.3E+07	4.4E+07	36	7	1287551	4.7201	1.2E-06	ARHGAP27	1509	4.1E-08
ENSG0000(	17	4.4E+07	4.4E+07	37	12	1186894	4.9481	3.8E-07	PLEKHM1	1509	4.1E-08
ENSG0000(	17	4.4E+07	4.4E+07	227	43	1318454	7.795	3.2E-15	CRHR1	1509	4.1E-08
ENSG0000(	17	4.4E+07	4.4E+07	4	2	1444713	3.6945	0.00011	SPPL2C	1509	4.1E-08
ENSG0000(	17	4.4E+07	4.4E+07	148	36	1266167	6.7583	7E-12	MAPT	1509	4.1E-08
ENSG0000(	17	4.4E+07	4.4E+07	173	25	1239074	7.6079	1.4E-14	KANSL1	1509	4.1E-08
ENSG0000(	17	4.4E+07	4.4E+07	8	3	139582	1.6303	Non_Signif	ARL17B	1509	4.1E-08
ENSG0000(	17	4.4E+07	4.4E+07	1	1	27693	0.56658	Non_Signif	LRRC37A	1509	4.1E-08
ENSG0000(	17	4.5E+07	4.5E+07	2	1	26625	2.9655	Non_Signif	LRRC37A2	1509	4.1E-08
ENSG0000(	17	4.5E+07	4.5E+07	2	1	26625	2.9655	Non_Signif	ARL17A	1509	4.1E-08
ENSG0000(	17	4.5E+07	4.5E+07	57	9	1154938	6.1094	5E-10	NSF	1509	4.1E-08
ENSG0000(	17	4.5E+07	4.5E+07	109	33	1256397	8.2924	5.6E-17	WNT3	1509	4.1E-08
ENSG0000(	17	4.5E+07	4.6E+07	314	15	1415727	0.7689	Non_Signif	EFCAB13	1509	1.8E-06
ENSG0000(	17	6.3E+07	6.3E+07	4	2	122369	-2.3069	Non_Signif	LRRC37A3	1519	4.1E-08
ENSG0000(	19	4.2E+07	4.2E+07	25	2	1419096	0.14708	Non_Signif	RPS19	1606	3.4E-08
ENSG0000(	19	4.2E+07	4.3E+07	29	14	1078517	0.77531	Non_Signif	ATP1A3	1606	3.4E-08
ENSG0000(	19	4.3E+07	4.3E+07	55	15	1222281	1.1197	Non_Signif	GRIK5	1606	3.4E-08
ENSG0000(	19	4.3E+07	4.3E+07	4	2	1438668	2.1313	Non_Signif	ZNF574	1606	9.9E-07
ENSG0000(	19	4.3E+07	4.3E+07	131	11	1293748	1.0175	Non_Signif	POU2F2	1606	7.6E-08
ENSG0000(	19	4.3E+07	4.3E+07	6	4	1141702	1.7243	Non_Signif	ZNF526	1606	1.3E-07
ENSG0000(	19	4.3E+07	4.3E+07	10	2	1449724	1.1401	Non_Signif	GSK3A	1606	1.3E-07
ENSG0000(	19	4.3E+07	4.3E+07	24	2	1464252	0.83377	Non_Signif	AC006486.	1606	1.3E-07
ENSG0000(	19	4.3E+07	4.3E+07	7	2	1474097	0.93797	Non_Signif	AC006486.	1606	1.3E-07
ENSG0000(	19	4.3E+07	4.3E+07	15	7	1014036	-0.04989	Non_Signif	LIPE	1606	6.3E-07
ENSG0000(	19	4.3E+07	4.3E+07	17	2	1329479	-0.76157	Non_Signif	CXCL17	1606	6.3E-07
ENSG0000(	20	3E+07	3E+07	7	2	208671	-2.1882	Non_Signif	FRG1B	1635	6.7E-25
ENSG0000(	20	3E+07	3E+07	5	2	1462867	1.59	Non_Signif	DEFB115	1635	3.9E-08

ENSG0000(	20	3E+07	3E+07	14	5	1192121	1.057	Non_Signif	DEFB116	1635	2.1E-09
ENSG0000(	20	3E+07	3E+07	7	3	1466105	0.56639	Non_Signif	DEFB118	1635	2.1E-09
ENSG0000(	20	3E+07	3E+07	40	9	1416500	2.7599	Non_Signif	DEFB119	1635	2.1E-09
ENSG0000(	20	3E+07	3E+07	18	4	1465365	2.7923	Non_Signif	DEFB121	1635	1.4E-10
ENSG0000(	20	3E+07	3E+07	24	5	1409979	2.3631	Non_Signif	DEFB123	1635	8.6E-11
ENSG0000(	20	3E+07	3E+07	18	5	1455803	2.3594	Non_Signif	DEFB124	1635	3.4E-16
ENSG0000(	20	3E+07	3E+07	16	6	1306635	2.2655	Non_Signif	REM1	1635	3.4E-16
ENSG0000(	20	3E+07	3E+07	114	7	1361286	3.8709	5.4E-05	HM13	1635	2.7E-09
ENSG0000(	20	3E+07	3E+07	8	4	1030031	3.3933	0.00035	ID1	1635	5E-09
ENSG0000(	20	3E+07	3E+07	14	2	1456132	4.6912	1.4E-06	COX4I2	1635	2.3E-35
ENSG0000(	20	3E+07	3E+07	94	9	1322813	4.8943	4.9E-07	BCL2L1	1635	2.3E-35
ENSG0000(	20	3E+07	3E+07	98	10	1411583	4.8387	6.5E-07	TPX2	1635	6.2E-35
ENSG0000(	20	3E+07	3E+07	43	7	1430985	4.5495	2.7E-06	MYLK2	1635	2.3E-35
ENSG0000(	20	3E+07	3E+07	2	1	1460059	4.3093	8.2E-06	FOXS1	1635	1.6E-34
ENSG0000(	20	3E+07	3E+07	59	18	1335906	3.5976	0.00016	DUSP15	1635	3.2E-34
ENSG0000(	20	3E+07	3.1E+07	149	24	1400319	1.9801	Non_Signif	TTLL9	1635	1.2E-22
ENSG0000(	20	3.1E+07	3.1E+07	6	2	1327730	0.44204	Non_Signif	PDRG1	1635	4.2E-35
ENSG0000(	20	3.1E+07	3.1E+07	76	21	1325600	-0.73843	Non_Signif	XKR7	1635	2.3E-35
ENSG0000(	20	3.1E+07	3.1E+07	43	10	1389396	0.25176	Non_Signif	CCM2L	1635	2.3E-35
ENSG0000(	20	3.1E+07	3.1E+07	90	13	1372169	1.0627	Non_Signif	HCK	1635	5.4E-09
ENSG0000(	20	3.1E+07	3.1E+07	111	10	1396583	-0.08615	Non_Signif	TM9SF4	1635	2.3E-35
ENSG0000(	20	3.1E+07	3.1E+07	12	3	1175933	-0.07234	Non_Signif	PLAGL2	1635	2.3E-35
ENSG0000(	20	3.1E+07	3.1E+07	34	6	1395129	0.05792	Non_Signif	POFUT1	1635	2.3E-35
ENSG0000(	20	3.1E+07	3.1E+07	184	26	1290667	-0.29214	Non_Signif	C20orf112	1635	4.9E-14
ENSG0000(	20	3.1E+07	3.1E+07	130	10	1423241	3.3712	0.00037	COMMD7	1635	6.5E-34

PD_GWAS_TOP_GENE	SIGNIFICANT_IN_BOTH_AND_IN_SEGMENT_OF_INTEREST
FALSE	FALSE
FALSE	FALSE
FALSE	FALSE
FALSE	FALSE
FALSE	FALSE
FALSE	FALSE
FALSE	FALSE
FALSE	FALSE
FALSE	FALSE
FALSE	FALSE
FALSE	FALSE
FALSE	FALSE
FALSE	FALSE
FALSE	FALSE
FALSE	FALSE
FALSE	FALSE
FALSE	FALSE
FALSE	FALSE
FALSE	FALSE
FALSE	FALSE
FALSE	FALSE
FALSE	FALSE
FALSE	FALSE
FALSE	FALSE
FALSE	FALSE
FALSE	FALSE
FALSE	TRUE
FALSE	FALSE
FALSE	TRUE
FALSE	TRUE
FALSE	TRUE
FALSE	TRUE
FALSE	TRUE

FALSE	TRUE
FALSE	TRUE
FALSE	TRUE
FALSE	TRUE
FALSE	FALSE
FALSE	TRUE
FALSE	FALSE
FALSE	FALSE
FALSE	TRUE
FALSE	TRUE
TRUE	TRUE
FALSE	TRUE
FALSE	TRUE
FALSE	TRUE
FALSE	FALSE
FALSE	FALSE
FALSE	FALSE
FALSE	FALSE
FALSE	TRUE
FALSE	TRUE
FALSE	FALSE
FALSE	FALSE
FALSE	FALSE
FALSE	FALSE
FALSE	FALSE
FALSE	FALSE
FALSE	FALSE
FALSE	FALSE
FALSE	FALSE
FALSE	FALSE
FALSE	FALSE
FALSE	FALSE
FALSE	FALSE
FALSE	FALSE
FALSE	FALSE

FALSE	FALSE
FALSE	FALSE
FALSE	FALSE
FALSE	FALSE
FALSE	FALSE
FALSE	FALSE
FALSE	FALSE
FALSE	TRUE
FALSE	TRUE
FALSE	TRUE
FALSE	TRUE
FALSE	TRUE
FALSE	TRUE
FALSE	TRUE
FALSE	TRUE
FALSE	FALSE
FALSE	FALSE
FALSE	FALSE
FALSE	FALSE
FALSE	FALSE
FALSE	FALSE
FALSE	FALSE
FALSE	FALSE
FALSE	FALSE
FALSE	TRUE

Supplementary Table 5. Statistically significant MAGMA results for the PD and the volume of the caudate											
GENE	CHR	START	STOP	NSNPS	NPARAM	N	ZSTAT	P_PD	SYMBOL	_SEGMENT	_Accumber
ENSG0000(	4	1E+08	1E+08	30	9	1359603	0.85564	Non_Signif	DDIT4L	465	1.9E-12
ENSG0000(	4	1E+08	1E+08	1765	91	1360987	2.2776	Non_Signif	BANK1	465	7.8E-11
ENSG0000(	4	1E+08	1E+08	522	34	1357547	1.8529	Non_Signif	SLC39A8	465	1.9E-12
ENSG0000(	4	1E+08	1E+08	243	18	1416064	-0.53996	Non_Signif	NFKB1	466	1.2E-07
ENSG0000(	4	1E+08	1E+08	156	9	1375227	1.7663	Non_Signif	UBE2D3	466	4.6E-08
ENSG0000(	4	1E+08	1E+08	35	4	1323152	1.6519	Non_Signif	CISD2	466	4.6E-08
ENSG0000(	4	1E+08	1E+08	251	10	1361410	2.1118	Non_Signif	SLC9B1	466	4.1E-08
ENSG0000(	4	1E+08	1E+08	85	22	1264979	1.3826	Non_Signif	SLC9B2	466	4.1E-08
ENSG0000(	4	1E+08	1E+08	49	7	1380425	-0.03464	Non_Signif	BDH2	466	4.1E-08
ENSG0000(	5	5.1E+07	5.1E+07	21	7	1423711	0.64442	Non_Signif	ISL1	550	3.6E-11
ENSG0000(	5	5.1E+07	5.1E+07	229	13	1427619	-0.46964	Non_Signif	CTD-2203A	550	2.6E-11
ENSG0000(	5	5.2E+07	5.2E+07	566	61	1391992	1.1833	Non_Signif	ITGA1	550	2.7E-10
ENSG0000(	5	5.2E+07	5.2E+07	36	8	1449652	0.83262	Non_Signif	PELO	550	2.7E-10
ENSG0000(	7	2.3E+07	2.3E+07	64	8	1450248	2.8521	Non_Signif	TOMM7	762	3E-08
ENSG0000(	7	2.3E+07	2.3E+07	60	6	1389229	5.7888	3.5E-09	NUPL2	762	3E-08
ENSG0000(	7	2.3E+07	2.3E+07	66	10	1348806	6.1094	5E-10	GPNMB	762	3E-08
ENSG0000(	7	2.3E+07	2.3E+07	22	6	1447103	5.6015	1.1E-08	MALSU1	762	3E-08
ENSG0000(	7	2.3E+07	2.4E+07	352	43	1406960	5.9333	1.5E-09	IGF2BP3	762	3E-08
ENSG0000(	7	9.9E+07	9.9E+07	41	7	1436971	0.59135	Non_Signif	CPSF4	803	6.8E-10
ENSG0000(	7	9.9E+07	9.9E+07	41	12	1338924	0.98241	Non_Signif	ZNF789	803	3.5E-13
ENSG0000(	7	9.9E+07	9.9E+07	45	10	1389923	0.94966	Non_Signif	ZKSCAN5	803	8.2E-11
ENSG0000(	7	9.9E+07	9.9E+07	87	9	1440876	0.46792	Non_Signif	CYP3A43	803	1.8E-09
ENSG0000(	7	9.9E+07	9.9E+07	3	1	1474097	2.2939	Non_Signif	OR2AE1	803	1.2E-11
ENSG0000(	7	9.9E+07	1E+08	88	8	1442287	1.7826	Non_Signif	TRIM4	803	1.7E-11
ENSG0000(	7	1E+08	1E+08	14	5	1466159	2.1191	Non_Signif	AZGP1	803	1.4E-10
ENSG0000(	7	1E+08	1E+08	17	3	1434508	1.1247	Non_Signif	ZSCAN21	803	8.2E-14
ENSG0000(	7	1E+08	1E+08	34	10	1375034	0.90315	Non_Signif	ZNF3	803	1.7E-11
ENSG0000(	7	1E+08	1E+08	4	2	1446315	0.42969	Non_Signif	COPS6	803	1.7E-11
ENSG0000(	7	1E+08	1E+08	24	5	1382562	0.84571	Non_Signif	MCM7	803	1.2E-11
ENSG0000(	7	1E+08	1E+08	21	5	1376229	-0.15333	Non_Signif	AP4M1	803	7.3E-11
ENSG0000(	7	1E+08	1E+08	21	6	1349778	0.34531	Non_Signif	TAF6	803	6E-11
ENSG0000(	7	1E+08	1E+08	7	3	1426776	1.3147	Non_Signif	CNPY4	803	6E-11
ENSG0000(	7	1E+08	1E+08	2	1	1246579	1.8492	Non_Signif	MBLAC1	803	2.6E-11

ENSG0000(	7	1E+08	1E+08	9	3	1423300	1.4056	Non_Signif	LAMTOR4	803	8.2E-14
ENSG0000(	7	1E+08	1E+08	12	3	1417834	0.49383	Non_Signif	GAL3ST4	803	8.2E-11
ENSG0000(	7	1E+08	1E+08	10	5	1250615	0.73501	Non_Signif	GPC2	803	8.2E-14
ENSG0000(	7	1E+08	1E+08	105	9	1433044	1.558	Non_Signif	STAG3	803	3.5E-13
ENSG0000(	7	1E+08	1E+08	133	17	1387180	1.5138	Non_Signif	GATS	803	6E-11
ENSG0000(	7	1E+08	1E+08	133	17	1387180	1.5138	Non_Signif	GATS	803	2.6E-11
ENSG0000(	7	1E+08	1E+08	11	4	1411302	1.2864	Non_Signif	PVRIG	803	2.6E-11
ENSG0000(	7	1E+08	1E+08	6	2	1379736	2.7614	Non_Signif	SPDYE3	803	8.8E-10
ENSG0000(	7	1E+08	1E+08	46	5	1373514	2.9308	Non_Signif	PILRB	803	8.2E-14
ENSG0000(	7	1E+08	1E+08	45	7	1393688	3.4176	0.00032	PILRA	803	8.2E-14
ENSG0000(	7	1E+08	1E+08	40	5	1420898	4.0988	2.1E-05	ZCWPW1	803	8.2E-14
ENSG0000(	7	1E+08	1E+08	14	4	953816	3.296	Non_Signif	MEPCE	803	8.2E-14
ENSG0000(	7	1E+08	1E+08	4	2	1474097	4.3889	5.7E-06	C7orf61	803	8.2E-14
ENSG0000(	7	1E+08	1E+08	20	4	1428375	4.2845	9.2E-06	TSC22D4	803	8.2E-14
ENSG0000(	7	1E+08	1E+08	21	6	1308285	4.445	4.4E-06	NYAP1	803	8.2E-14
ENSG0000(	7	1E+08	1E+08	33	6	1331495	2.3319	Non_Signif	AGFG2	803	7.3E-11
ENSG0000(	7	1E+08	1E+08	2	1	1192084	1.6307	Non_Signif	SAP25	803	8.2E-14
ENSG0000(	7	1E+08	1E+08	30	9	1319908	1.8522	Non_Signif	LRCH4	803	8.2E-14
ENSG0000(	7	1E+08	1E+08	17	7	1420483	1.5077	Non_Signif	FBXO24	803	1.7E-11
ENSG0000(	10	1E+08	1E+08	115	21	1344437	0.30836	Non_Signif	PKD2L1	1073	1.7E-08
ENSG0000(	10	1E+08	1E+08	67	5	1439856	0.54567	Non_Signif	SEC31B	1073	1.7E-08
ENSG0000(	10	1E+08	1E+08	40	3	1423402	0.57951	Non_Signif	NDUFB8	1073	1.7E-08
ENSG0000(	10	1E+08	1E+08	40	3	1423402	0.57951	Non_Signif	NDUFB8	1073	1.7E-08
ENSG0000(	10	1E+08	1E+08	36	3	1417769	0.55726	Non_Signif	NDUFB8	1073	1.7E-08
ENSG0000(	10	1E+08	1E+08	36	3	1417769	0.55726	Non_Signif	NDUFB8	1073	1.7E-08
ENSG0000(	10	1E+08	1E+08	85	4	1397770	0.55506	Non_Signif	HIF1AN	1073	1.7E-08
ENSG0000(	10	1E+08	1E+08	80	14	1398877	2.3262	Non_Signif	FAM178A	1073	1.7E-08
ENSG0000(	10	1E+08	1E+08	29	6	1451382	2.8886	Non_Signif	MRPL43	1073	1.7E-08
ENSG0000(	10	1E+08	1E+08	25	5	1452108	2.8013	Non_Signif	SEMA4G	1073	2.3E-08
ENSG0000(	10	1E+08	1E+08	13	2	1465384	3.219	Non_Signif	C10orf2	1073	1.7E-08
ENSG0000(	10	1E+08	1E+08	29	6	1423850	3.4302	0.0003	LZTS2	1073	1.7E-08
ENSG0000(	10	1E+08	1E+08	69	22	1277347	1.7219	Non_Signif	PDZD7	1073	1.7E-08
ENSG0000(	10	1E+08	1E+08	18	11	1192902	2.1201	Non_Signif	SFXN3	1073	1.7E-08
ENSG0000(	10	1E+08	1E+08	8	4	1415196	2.5752	Non_Signif	KAZALD1	1073	1.7E-08
ENSG0000(	10	1E+08	1E+08	3	2	1393626	-0.07089	Non_Signif	HUG1	1073	1.7E-08

ENSG0000	10	1E+08	1E+08	17	6	1427235	2.4663	Non_Signif	TLX1	1073	1.7E-08
ENSG0000	14	9.2E+07	9.2E+07	437	62	1367579	0.78351	Non_Signif	CCDC88C	1368	8.5E-12
ENSG0000	14	9.2E+07	9.2E+07	80	17	1289358	2.8835	Non_Signif	SMEK1	1368	1.5E-09
ENSG0000	14	9.2E+07	9.2E+07	7	3	1328556	1.6603	Non_Signif	AL133373.	1368	7.8E-09
ENSG0000	14	9.2E+07	9.2E+07	657	63	1373465	1.7076	Non_Signif	CATSPERB	1368	9.6E-09
ENSG0000	14	9.2E+07	9.3E+07	156	17	1426566	-1.3631	Non_Signif	TRIP11	1368	8.7E-12
ENSG0000	16	2.8E+07	2.8E+07	127	10	1298179	0.99045	Non_Signif	XPO6	1451	1.8E-12
ENSG0000	16	2.8E+07	2.8E+07	46	14	1203651	1.4279	Non_Signif	SBK1	1451	3.5E-12
ENSG0000	16	2.8E+07	2.9E+07	14	5	564629	1.335	Non_Signif	CLN3	1451	2.1E-15
ENSG0000	16	2.8E+07	2.9E+07	14	5	564629	1.335	Non_Signif	CLN3	1451	1.6E-12
ENSG0000	16	2.8E+07	2.9E+07	17	7	639090	1.4032	Non_Signif	CLN3	1451	2.1E-15
ENSG0000	16	2.8E+07	2.9E+07	17	7	639090	1.4032	Non_Signif	CLN3	1451	1.6E-12
ENSG0000	16	2.9E+07	2.9E+07	5	3	581146	0.19996	Non_Signif	APOBR	1451	7.2E-13
ENSG0000	16	2.9E+07	2.9E+07	15	4	1128751	-0.82297	Non_Signif	IL27	1451	2.1E-15
ENSG0000	16	2.9E+07	2.9E+07	7	2	1207864	1.3701	Non_Signif	NUPR1	1451	1.3E-15
ENSG0000	16	2.9E+07	2.9E+07	78	17	1007931	1.6601	Non_Signif	CCDC101	1451	1.6E-12
ENSG0000	16	2.9E+07	2.9E+07	18	8	956879	1.0048	Non_Signif	SULT1A2	1451	1.3E-15
ENSG0000	16	2.9E+07	2.9E+07	40	11	512811	3.2711	Non_Signif	SULT1A1	1451	1.3E-15
ENSG0000	16	2.9E+07	2.9E+07	1	1	15976	1.4837	Non_Signif	EIF3C	1451	2.1E-15
ENSG0000	16	2.9E+07	2.9E+07	27	3	1298772	5.2122	9.3E-08	ATXN2L	1451	1.3E-15
ENSG0000	16	2.9E+07	2.9E+07	6	1	1339784	4.8751	5.4E-07	TUFM	1451	1.3E-15
ENSG0000	16	2.9E+07	2.9E+07	47	4	1260457	5.1361	1.4E-07	SH2B1	1451	1.3E-15
ENSG0000	16	2.9E+07	2.9E+07	27	6	1237705	5.5829	1.2E-08	ATP2A1	1451	2.2E-15
ENSG0000	16	2.9E+07	2.9E+07	40	11	1090570	5.4499	2.5E-08	RABEP2	1451	1.3E-15
ENSG0000	16	2.9E+07	2.9E+07	10	5	998004	4.5274	3E-06	CD19	1451	1.3E-15
ENSG0000	16	2.9E+07	2.9E+07	28	8	1128945	3.7394	9.2E-05	NFATC2IP	1451	1.3E-15
ENSG0000	16	2.9E+07	2.9E+07	23	7	1087907	5.1938	1E-07	SPNS1	1451	2.2E-15
ENSG0000	16	2.9E+07	2.9E+07	11	5	991708	3.7524	8.8E-05	LAT	1451	1.3E-15
ENSG0000	16	7.1E+07	7.1E+07	310	38	1009834	-1.1366	Non_Signif	HYDIN	1466	3.6E-05
ENSG0000	16	7.1E+07	7.1E+07	32	9	1467218	1.4427	Non_Signif	ZNF23	1466	4.9E-08
ENSG0000	16	7.2E+07	7.2E+07	160	19	1415536	2.2109	Non_Signif	PHLPP2	1466	3.4E-10
ENSG0000	16	7.2E+07	7.2E+07	70	12	1406548	2.9511	Non_Signif	ATXN1L	1466	3.4E-10
ENSG0000	16	7.2E+07	7.2E+07	50	9	1433206	2.2528	Non_Signif	DHODH	1466	3.4E-10
ENSG0000	16	7.2E+07	7.2E+07	140	18	1391967	1.5027	Non_Signif	TXNL4B	1466	9.1E-09
ENSG0000	16	7.2E+07	7.2E+07	11	4	1380099	1.2875	Non_Signif	HP	1466	3.4E-10

ENSG0000(	16	7.2E+07	7.2E+07	50	11	1383299	1.3742	Non_Signif	HPR	1466	3.4E-10
ENSG0000(	16	7.2E+07	7.2E+07	56	8	1413667	1.7679	Non_Signif	DHX38	1466	3.4E-10
ENSG0000(	16	7.2E+07	7.2E+07	174	21	1423784	1.3078	Non_Signif	PMFBP1	1466	5.4E-09
ENSG0000(	16	7.3E+07	7.3E+07	796	97	1325461	0.25796	Non_Signif	ZFHX3	1466	1.9E-06
ENSG0000(	20	3E+07	3E+07	7	2	208671	-2.1882	Non_Signif	FRG1B	1635	3.7E-14
ENSG0000(	20	3E+07	3E+07	18	5	1455803	2.3594	Non_Signif	DEFB124	1635	4.1E-10
ENSG0000(	20	3E+07	3E+07	16	6	1306635	2.2655	Non_Signif	REM1	1635	4.1E-10
ENSG0000(	20	3E+07	3E+07	114	7	1361286	3.8709	5.4E-05	HM13	1635	0.00015
ENSG0000(	20	3E+07	3E+07	8	4	1030031	3.3933	0.00035	ID1	1635	0.00015
ENSG0000(	20	3E+07	3E+07	14	2	1456132	4.6912	1.4E-06	COX4I2	1635	3.3E-21
ENSG0000(	20	3E+07	3E+07	94	9	1322813	4.8943	4.9E-07	BCL2L1	1635	4.1E-21
ENSG0000(	20	3E+07	3E+07	98	10	1411583	4.8387	6.5E-07	TPX2	1635	3.6E-21
ENSG0000(	20	3E+07	3E+07	43	7	1430985	4.5495	2.7E-06	MYLK2	1635	3.3E-21
ENSG0000(	20	3E+07	3E+07	2	1	1460059	4.3093	8.2E-06	FOXS1	1635	6.7E-21
ENSG0000(	20	3E+07	3E+07	59	18	1335906	3.5976	0.00016	DUSP15	1635	3E-20
ENSG0000(	20	3E+07	3.1E+07	149	24	1400319	1.9801	Non_Signif	TTLL9	1635	1.3E-13
ENSG0000(	20	3.1E+07	3.1E+07	6	2	1327730	0.44204	Non_Signif	PDRG1	1635	3.3E-21
ENSG0000(	20	3.1E+07	3.1E+07	76	21	1325600	-0.73843	Non_Signif	XKR7	1635	3.3E-21
ENSG0000(	20	3.1E+07	3.1E+07	43	10	1389396	0.25176	Non_Signif	CCM2L	1635	3.3E-21
ENSG0000(	20	3.1E+07	3.1E+07	90	13	1372169	1.0627	Non_Signif	HCK	1635	0.00025
ENSG0000(	20	3.1E+07	3.1E+07	111	10	1396583	-0.08615	Non_Signif	TM9SF4	1635	3.3E-21
ENSG0000(	20	3.1E+07	3.1E+07	12	3	1175933	-0.07234	Non_Signif	PLAGL2	1635	1.4E-20
ENSG0000(	20	3.1E+07	3.1E+07	34	6	1395129	0.05792	Non_Signif	POFUT1	1635	1.4E-20
ENSG0000(	20	3.1E+07	3.1E+07	130	10	1423241	3.3712	0.00037	COMMD7	1635	1.7E-20
ENSG0000(	21	4.1E+07	4.2E+07	3209	210	1356932	6.3602	1E-10	DSCAM	1674	6.9E-14
ENSG0000(	21	4.3E+07	4.3E+07	358	61	1332662	-0.72655	Non_Signif	BACE2	1674	4.7E-11

PD_GWAS_TOP_GENE	SIGNIFICANT_IN_BOTH_AND_IN_SEGMENT_OF_INTEREST
FALSE	FALSE
FALSE	FALSE
FALSE	FALSE
FALSE	FALSE
FALSE	FALSE
FALSE	FALSE
FALSE	FALSE
FALSE	FALSE
FALSE	FALSE
FALSE	FALSE
FALSE	FALSE
FALSE	FALSE
FALSE	FALSE
FALSE	FALSE
FALSE	TRUE
TRUE	TRUE
FALSE	TRUE
FALSE	TRUE
FALSE	FALSE
FALSE	FALSE
FALSE	FALSE
FALSE	FALSE
FALSE	FALSE
FALSE	FALSE
FALSE	FALSE
FALSE	FALSE
FALSE	FALSE
FALSE	FALSE
FALSE	FALSE
FALSE	FALSE
FALSE	FALSE
FALSE	FALSE
FALSE	FALSE

FALSE	FALSE
FALSE	FALSE
FALSE	FALSE
FALSE	FALSE
FALSE	FALSE
FALSE	FALSE
FALSE	FALSE
FALSE	FALSE
FALSE	FALSE
FALSE	TRUE
FALSE	TRUE
FALSE	FALSE
FALSE	TRUE
FALSE	TRUE
FALSE	TRUE
FALSE	FALSE
FALSE	FALSE
FALSE	FALSE
FALSE	FALSE
FALSE	FALSE
FALSE	FALSE
FALSE	FALSE
FALSE	FALSE
FALSE	FALSE
FALSE	FALSE
FALSE	FALSE
FALSE	FALSE
FALSE	FALSE
FALSE	FALSE
FALSE	FALSE
FALSE	FALSE
FALSE	FALSE
FALSE	FALSE
FALSE	FALSE
FALSE	FALSE

FALSE	FALSE
FALSE	FALSE
FALSE	FALSE
FALSE	FALSE
FALSE	FALSE
FALSE	FALSE
FALSE	FALSE
FALSE	FALSE
FALSE	FALSE
FALSE	FALSE
FALSE	FALSE
FALSE	FALSE
FALSE	FALSE
FALSE	FALSE
FALSE	FALSE
FALSE	FALSE
FALSE	FALSE
FALSE	FALSE
FALSE	FALSE
FALSE	TRUE
FALSE	TRUE
FALSE	TRUE
FALSE	TRUE
FALSE	TRUE
FALSE	TRUE
FALSE	TRUE
FALSE	TRUE
FALSE	TRUE
FALSE	FALSE
FALSE	FALSE
FALSE	FALSE
FALSE	FALSE
FALSE	FALSE
FALSE	FALSE
FALSE	FALSE

FALSE	FALSE
FALSE	FALSE
FALSE	FALSE
FALSE	FALSE
FALSE	FALSE
FALSE	FALSE
FALSE	FALSE
FALSE	TRUE
FALSE	TRUE
FALSE	TRUE
FALSE	TRUE
FALSE	TRUE
FALSE	TRUE
FALSE	TRUE
FALSE	TRUE
FALSE	FALSE
FALSE	FALSE
FALSE	FALSE
FALSE	FALSE
FALSE	FALSE
FALSE	FALSE
FALSE	FALSE
FALSE	FALSE
FALSE	TRUE
FALSE	FALSE
FALSE	FALSE

Supplementary Table 6. Statistically significant MAGMA results for the PD and the volume of the brainstem										
GENE	CHR	START	STOP	NSNPS	NPARAM	N	ZSTAT	P_PD	SYMBOL	_SEGMENT
ENSG0000(	2	2.7E+07	2.7E+07	34	8	1386631	1.7894	Non_Significant	SLC5A6	148
ENSG0000(	2	2.7E+07	2.7E+07	15	5	1436353	1.7462	Non_Significant	ATRAID	148
ENSG0000(	2	2.8E+07	2.8E+07	22	9	1349197	2.2968	Non_Significant	MPV17	148
ENSG0000(	2	2.8E+07	2.8E+07	24	6	1376095	3.5748	0.00017522	GTF3C2	148
ENSG0000(	2	2.8E+07	2.8E+07	10	6	1416291	3.8005	0.0000722	EIF2B4	148
ENSG0000(	2	2.8E+07	2.8E+07	9	3	1411190	3.5225	0.00021378	SNX17	148
ENSG0000(	2	2.8E+07	2.8E+07	62	9	1405389	3.5222	0.00021398	IFT172	148
ENSG0000(	2	2.8E+07	2.8E+07	37	7	1437156	3.0678	Non_Significant	GCKR	148
ENSG0000(	2	2.8E+07	2.8E+07	10	4	1417267	1.0012	Non_Significant	SUPT7L	148
ENSG0000(	2	2.8E+07	2.8E+07	30	12	1359258	0.45321	Non_Significant	SLC4A1AP	148
ENSG0000(	2	3.2E+07	3.2E+07	142	17	1348274	2.7724	Non_Significant	SLC30A6	152
ENSG0000(	2	3.3E+07	3.3E+07	78	16	1394543	2.9663	Non_Significant	YIPF4	152
ENSG0000(	2	3.3E+07	3.3E+07	720	47	1410484	2.564	Non_Significant	TTC27	152
ENSG0000(	2	3.3E+07	3.4E+07	1895	78	1427064	1.1835	Non_Significant	LTBP1	152
ENSG0000(	2	3.4E+07	3.4E+07	423	66	1353348	2.3786	Non_Significant	RASGRP3	153
ENSG0000(	2	3.4E+07	3.4E+07	42	10	1395668	0.62902	Non_Significant	FAM98A	153
ENSG0000(	2	8.8E+07	8.8E+07	44	7	1340425	1.6042	Non_Significant	KRCC1	187
ENSG0000(	2	8.8E+07	8.8E+07	6	3	1375413	0.67983	Non_Significant	FABP1	187
ENSG0000(	2	8.8E+07	8.8E+07	41	14	1373971	2.5999	Non_Significant	THNSL2	187
ENSG0000(	2	8.9E+07	8.9E+07	86	17	1275253	2.5371	Non_Significant	EIF2AK3	187
ENSG0000(	2	8.9E+07	8.9E+07	136	9	1410025	1.779	Non_Significant	RPIA	187
ENSG0000(	3	5.2E+07	5.2E+07	39	14	1385209	0.36902	Non_Significant	TEX264	311
ENSG0000(	3	5.2E+07	5.2E+07	21	3	1414940	1.4275	Non_Significant	IQCF1	312
ENSG0000(	3	5.2E+07	5.2E+07	14	8	1290801	-0.5866	Non_Significant	ABHD14B	312
ENSG0000(	3	5.2E+07	5.2E+07	6	3	1419602	0.69824	Non_Significant	DUSP7	312
ENSG0000(	3	5.2E+07	5.2E+07	101	14	1298299	0.21629	Non_Significant	POC1A	312
ENSG0000(	3	5.2E+07	5.2E+07	3	1	1437767	1.2384	Non_Significant	PPM1M	312
ENSG0000(	3	5.2E+07	5.2E+07	14	4	1466312	3.5828	0.00016995	GLYCTK	312
ENSG0000(	3	5.2E+07	5.2E+07	6	2	1209642	3.8851	0.0000511	BAP1	312
ENSG0000(	3	5.2E+07	5.2E+07	12	6	1435821	4.6611	0.00000157	PHF7	312
ENSG0000(	3	5.2E+07	5.2E+07	15	7	1400455	3.5908	0.00016483	SEMA3G	312
ENSG0000(	3	5.2E+07	5.2E+07	3	2	986747	3.8034	0.0000714	TNNC1	312
ENSG0000(	3	5.2E+07	5.3E+07	73	6	1407017	3.7408	0.0000917	NISCH	312

ENSG0000(	3	5.3E+07	5.3E+07	52	15	1298834	4.0584		0.0000247	STAB1	312
ENSG0000(	3	5.3E+07	5.3E+07	26	5	1435389	3.1411	Non_Significant		NT5DC2	312
ENSG0000(	3	5.3E+07	5.3E+07	100	11	1416565	3.044	Non_Significant		SMIM4	312
ENSG0000(	3	5.3E+07	5.3E+07	310	11	1413960	3.103	Non_Significant		PBRM1	312
ENSG0000(	3	5.3E+07	5.3E+07	22	4	1469046	3.7499		0.0000884	GNL3	312
ENSG0000(	3	5.3E+07	5.3E+07	15	4	1316159	3.245	Non_Significant		GLT8D1	312
ENSG0000(	3	5.3E+07	5.3E+07	6	2	1417561	3.093	Non_Significant		SPCS1	312
ENSG0000(	3	5.3E+07	5.3E+07	120	10	1436630	3.1278	Non_Significant		NEK4	312
ENSG0000(	3	5.3E+07	5.3E+07	34	4	1424510	2.1089	Non_Significant		ITIH1	312
ENSG0000(	3	5.3E+07	5.3E+07	23	8	1380224	3.6098		0.00015324	ITIH3	312
ENSG0000(	3	5.3E+07	5.3E+07	45	7	1400832	3.5329		0.00020554	ITIH4	312
ENSG0000(	3	5.3E+07	5.3E+07	60	7	1402625	3.6199		0.00014733	RP5-966M:	312
ENSG0000(	3	5.3E+07	5.3E+07	7	2	1332473	3.4446		0.000286	MUSTN1	312
ENSG0000(	3	5.3E+07	5.3E+07	129	11	1422786	3.4714		0.00025884	TMEM110-	312
ENSG0000(	3	5.3E+07	5.3E+07	119	10	1427720	3.4596		0.00027051	TMEM110	312
ENSG0000(	3	5.3E+07	5.3E+07	288	17	1398425	4.2008		0.0000133	SFMBT1	312
ENSG0000(	3	5.3E+07	5.3E+07	102	9	1361794	2.1138	Non_Significant		RFT1	312
ENSG0000(	5	6.4E+07	6.4E+07	74	8	1356729	1.1372	Non_Significant		SREK1IP1	560
ENSG0000(	5	6.4E+07	6.4E+07	452	23	1410242	1.1395	Non_Significant		CWC27	560
ENSG0000(	5	6.4E+07	6.5E+07	750	42	1421304	1.2298	Non_Significant		ADAMTS6	560
ENSG0000(	5	6.5E+07	6.5E+07	146	12	1427851	2.1066	Non_Significant		CENPK	560
ENSG0000(	5	6.5E+07	6.5E+07	67	8	1450309	1.9521	Non_Significant		PPWD1	560
ENSG0000(	5	6.5E+07	6.5E+07	113	11	1420773	2.205	Non_Significant		TRIM23	560
ENSG0000(	5	6.5E+07	6.5E+07	117	10	1447649	1.8195	Non_Significant		TRAPPC13	560
ENSG0000(	5	6.5E+07	6.5E+07	16	4	1469855	2.1023	Non_Significant		CTC-534A2	560
ENSG0000(	5	6.5E+07	6.5E+07	98	16	1375807	1.7663	Non_Significant		SGTB	560
ENSG0000(	5	6.5E+07	6.5E+07	421	42	1408936	0.52104	Non_Significant		NLN	560
ENSG0000(	5	6.5E+07	6.5E+07	183	22	1349411	1.6989	Non_Significant		ERBB2IP	560
ENSG0000(	5	1.4E+08	1.4E+08	5	3	1312581	1.37	Non_Significant		EGR1	602
ENSG0000(	5	1.4E+08	1.4E+08	26	8	1396456	2.0201	Non_Significant		PAIP2	602
ENSG0000(	5	1.4E+08	1.4E+08	4	2	1226255	1.9923	Non_Significant		PROB1	602
ENSG0000(	5	1.4E+08	1.4E+08	8	2	1333090	1.1593	Non_Significant		SPATA24	602
ENSG0000(	5	1.4E+08	1.4E+08	65	11	1307721	0.9504	Non_Significant		CXXC5	602
ENSG0000(	5	1.4E+08	1.4E+08	359	36	1392318	-0.83794	Non_Significant		NRG2	602
ENSG0000(	5	1.6E+08	1.6E+08	399	22	1450223	1.0614	Non_Significant		CYFIP2	613

ENSG0000(	5	1.6E+08	1.6E+08	16	5	1431168	1.486	Non_Significant		FNDC9	613
ENSG0000(	5	1.6E+08	1.6E+08	617	32	1439497	1.7995	Non_Significant		ADAM19	613
ENSG0000(	5	1.6E+08	1.6E+08	46	8	1453529	0.97772	Non_Significant		NIPAL4	613
ENSG0000(	5	1.6E+08	1.6E+08	46	10	1424790	2.0274	Non_Significant		THG1L	613
ENSG0000(	5	1.6E+08	1.6E+08	7	2	1266509	-0.67321	Non_Significant		AC026407.	613
ENSG0000(	5	1.6E+08	1.6E+08	36	6	1430464	1.2716	Non_Significant		LSM11	613
ENSG0000(	8	1E+08	1E+08	374	35	1359961	-0.47255	Non_Significant		SNX31	910
ENSG0000(	8	1E+08	1E+08	67	9	1327812	2.3582	Non_Significant		PABPC1	910
ENSG0000(	8	1.2E+08	1.2E+08	502	27	1359246	3.0413	Non_Significant		TRPS1	919
ENSG0000(	9	1.3E+08	1.3E+08	453	33	1419495	2.6271	Non_Significant		MVB12B	1000
ENSG0000(	17	2.7E+07	2.7E+07	191	39	1366052	0.97556	Non_Significant		PIPOX	1500
ENSG0000(	17	2.7E+07	2.7E+07	47	7	1392324	1.2786	Non_Significant		TIAF1	1500
ENSG0000(	17	2.8E+07	2.8E+07	17	6	1324327	-1.3717	Non_Significant		CRYBA1	1500
ENSG0000(	17	2.8E+07	2.8E+07	82	12	1400063	-0.36047	Non_Significant		NUFIP2	1500
ENSG0000(	17	2.8E+07	2.8E+07	10	4	1271709	-0.25925	Non_Significant		CORO6	1500
ENSG0000(	17	2.9E+07	2.9E+07	36	8	1247640	0.493	Non_Significant		TMIGD1	1500
ENSG0000(	17	2.9E+07	2.9E+07	112	11	1363201	-0.02676	Non_Significant		CPD	1500
ENSG0000(	17	2.9E+07	2.9E+07	60	10	1346115	0.38031	Non_Significant		GOSR1	1500
ENSG0000(	17	2.9E+07	3E+07	354	10	1362579	1.3468	Non_Significant		NF1	1500
ENSG0000(	17	3E+07	3E+07	28	4	1406694	1.851	Non_Significant		OMG	1500
ENSG0000(	17	3E+07	3E+07	14	2	1385404	0.52265	Non_Significant		EVI2B	1500
ENSG0000(	17	3E+07	3E+07	18	2	1328537	0.39426	Non_Significant		CTD-2370N	1500
ENSG0000(	17	3E+07	3E+07	11	2	1216916	0.55994	Non_Significant		EVI2A	1500
ENSG0000(	17	3E+07	3E+07	419	52	1336799	1.5077	Non_Significant		RAB11FIP4	1500
ENSG0000(	17	4.3E+07	4.3E+07	7	3	1200483	2.9026	Non_Significant		HEXIM1	1509
ENSG0000(	17	4.3E+07	4.3E+07	50	8	1430881	1.8861	Non_Significant		FMNL1	1509
ENSG0000(	17	4.3E+07	4.4E+07	36	7	1287551	4.7201		0.00000118	ARHGAP27	1509
ENSG0000(	17	4.4E+07	4.4E+07	37	12	1186894	4.9481		0.000000375	PLEKHM1	1509
ENSG0000(	17	4.4E+07	4.4E+07	227	43	1318454	7.795		3.22E-15	CRHR1	1509
ENSG0000(	17	4.4E+07	4.4E+07	4	2	1444713	3.6945		0.00011016	SPPL2C	1509
ENSG0000(	17	4.4E+07	4.4E+07	148	36	1266167	6.7583		6.98E-12	MAPT	1509
ENSG0000(	17	4.4E+07	4.4E+07	173	25	1239074	7.6079		1.39E-14	KANSL1	1509
ENSG0000(	17	4.4E+07	4.4E+07	8	3	139582	1.6303	Non_Significant		ARL17B	1509
ENSG0000(	17	4.4E+07	4.4E+07	1	1	27693	0.56658	Non_Significant		LRRC37A	1509
ENSG0000(	17	4.5E+07	4.5E+07	2	1	26625	2.9655	Non_Significant		LRRC37A2	1509

ENSG0000(	17	4.5E+07	4.5E+07	2	1	26625	2.9655	Non_Significant		ARL17A	1509
ENSG0000(	17	4.5E+07	4.5E+07	57	9	1154938	6.1094		5E-10	NSF	1509
ENSG0000(	17	4.5E+07	4.5E+07	109	33	1256397	8.2924		5.55E-17	WNT3	1509
ENSG0000(	17	4.5E+07	4.6E+07	314	15	1415727	0.7689	Non_Significant		EFCAB13	1509
ENSG0000(	19	7112266	7294045	748	82	1303986	3.2521	Non_Significant		INSR	1584
ENSG0000(	19	7793843	7798792	21	6	846092	-0.84362	Non_Significant		CLEC4G	1584
ENSG0000(	20	3.2E+07	3.2E+07	47	11	1348007	3.4316		0.00029998	CDK5RAP1	1636
ENSG0000(	20	3.2E+07	3.2E+07	53	8	1410475	3.3488		0.0004058	SNTA1	1636
ENSG0000(	20	3.2E+07	3.2E+07	131	25	1319020	3.2449	Non_Significant		CBFA2T2	1636
ENSG0000(	20	3.2E+07	3.2E+07	14	5	1416931	2.6091	Non_Significant		NECAB3	1636
ENSG0000(	20	3.2E+07	3.2E+07	1	1	1474097	2.2304	Non_Significant		C20orf144	1636
ENSG0000(	20	3.2E+07	3.2E+07	3	1	1474097	2.0934	Non_Significant		ACTL10	1636
ENSG0000(	20	3.2E+07	3.2E+07	17	9	1364441	3.4501		0.00028015	E2F1	1636
ENSG0000(	20	3.2E+07	3.2E+07	28	5	1452456	2.5376	Non_Significant		PXMP4	1636
ENSG0000(	20	3.2E+07	3.2E+07	90	14	1383369	4.1132		0.0000195	ZNF341	1636
ENSG0000(	20	3.2E+07	3.2E+07	40	9	1361874	0.85852	Non_Significant		CHMP4B	1636
ENSG0000(	20	3.3E+07	3.3E+07	151	16	1348225	0.8922	Non_Significant		RALY	1636
ENSG0000(	20	3.3E+07	3.3E+07	25	6	1423236	1.454	Non_Significant		EIF2S2	1636

P_Accumbens	PD_GWAS_TOP_GENE	SIGNIFICANT_IN_BOTH_AND_IN_SEGMENT_OF_INTEREST
1.43316E-08	FALSE	FALSE
4.77507E-07	FALSE	FALSE
1.85403E-07	FALSE	FALSE
1.43316E-08	FALSE	FALSE
1.85403E-07	FALSE	FALSE
1.85403E-07	FALSE	FALSE
4.77507E-07	FALSE	FALSE
1.85403E-07	FALSE	FALSE
1.85403E-07	FALSE	FALSE
1.43316E-08	FALSE	FALSE
5.89086E-11	FALSE	FALSE
5.89086E-11	FALSE	FALSE
1.02891E-17	FALSE	FALSE
3.87905E-10	FALSE	FALSE
1.02891E-17	FALSE	FALSE
3.87905E-10	FALSE	FALSE
2.58041E-18	FALSE	FALSE
1.13343E-14	FALSE	FALSE
2.58041E-18	FALSE	FALSE
2.58041E-18	FALSE	FALSE
2.58041E-18	FALSE	FALSE
5.82767E-05	FALSE	FALSE
6.32859E-07	FALSE	FALSE
2.63263E-08	FALSE	FALSE
3.78825E-08	FALSE	FALSE
5.82778E-07	FALSE	FALSE
2.63263E-08	FALSE	FALSE
2.63263E-08	FALSE	TRUE
3.46376E-07	FALSE	TRUE
3.46376E-07	FALSE	TRUE
7.01586E-05	FALSE	TRUE
3.78825E-08	FALSE	TRUE
3.78825E-08	FALSE	TRUE

4.62947E-05	FALSE	TRUE
3.6212E-08	FALSE	FALSE
3.6212E-08	FALSE	FALSE
2.63263E-08	FALSE	FALSE
2.63263E-08	FALSE	TRUE
2.63263E-08	FALSE	FALSE
2.63263E-08	FALSE	FALSE
2.63263E-08	FALSE	FALSE
4.6972E-06	FALSE	FALSE
5.77916E-06	FALSE	TRUE
2.63263E-08	FALSE	TRUE
2.63263E-08	FALSE	TRUE
2.63263E-08	FALSE	TRUE
4.6972E-06	FALSE	TRUE
4.6972E-06	FALSE	TRUE
2.63263E-08	FALSE	TRUE
2.63263E-08	FALSE	FALSE
5.57992E-29	FALSE	FALSE
5.57992E-29	FALSE	FALSE
3.23306E-29	FALSE	FALSE
5.3642E-30	FALSE	FALSE
5.3642E-30	FALSE	FALSE
9.73051E-30	FALSE	FALSE
5.3642E-30	FALSE	FALSE
5.3642E-30	FALSE	FALSE
5.3642E-30	FALSE	FALSE
5.3642E-30	FALSE	FALSE
7.81392E-28	FALSE	FALSE
6.95419E-05	FALSE	FALSE
3.16294E-07	FALSE	FALSE
1.71062E-07	FALSE	FALSE
1.71062E-07	FALSE	FALSE
1.71062E-07	FALSE	FALSE
1.71062E-07	FALSE	FALSE
9.49968E-08	FALSE	FALSE

4.0994E-09	FALSE	FALSE
4.0994E-09	FALSE	FALSE
3.71492E-08	FALSE	FALSE
5.53448E-08	FALSE	FALSE
8.70604E-08	FALSE	FALSE
3.71492E-08	FALSE	FALSE
5.47458E-11	FALSE	FALSE
5.47458E-11	FALSE	FALSE
8.8863E-11	FALSE	FALSE
4.14016E-09	FALSE	FALSE
1.88544E-09	FALSE	FALSE
7.70281E-08	FALSE	FALSE
3.35636E-15	FALSE	FALSE
3.35636E-15	FALSE	FALSE
1.88544E-09	FALSE	FALSE
5.96846E-07	FALSE	FALSE
3.73528E-09	FALSE	FALSE
4.75257E-12	FALSE	FALSE
8.52426E-15	FALSE	FALSE
8.52426E-15	FALSE	FALSE
8.52426E-15	FALSE	FALSE
8.52426E-15	FALSE	FALSE
1.54731E-09	FALSE	FALSE
2.14803E-09	FALSE	FALSE
2.0996E-12	FALSE	FALSE
8.4113E-13	FALSE	FALSE
8.4113E-13	FALSE	TRUE
8.4113E-13	FALSE	TRUE
1.14088E-14	TRUE	TRUE
1.14088E-14	FALSE	TRUE
1.14088E-14	FALSE	TRUE
1.14088E-14	FALSE	TRUE
1.14088E-14	FALSE	FALSE
1.14088E-14	FALSE	FALSE
1.14088E-14	FALSE	FALSE

1.14088E-14	FALSE	FALSE
1.14088E-14	FALSE	TRUE
1.14088E-14	FALSE	TRUE
2.03522E-13	FALSE	FALSE
5.89537E-09	FALSE	FALSE
3.46798E-07	FALSE	FALSE
3.04635E-08	FALSE	FALSE
2.58075E-06	FALSE	FALSE
3.04635E-08	FALSE	FALSE
1.9431E-06	FALSE	FALSE
2.00238E-06	FALSE	FALSE
1.9431E-06	FALSE	FALSE
3.04635E-08	FALSE	FALSE
3.04635E-08	FALSE	FALSE
3.04635E-08	FALSE	FALSE
1.47548E-05	FALSE	FALSE
1.26872E-05	FALSE	FALSE
4.61054E-08	FALSE	FALSE

Supplementary Table 7. Statistically significant MAGMA results for the PD and the volume of the pallidum

GENE	CHR	START	STOP	NSNPS	NPARAM	N	ZSTAT	P_PD	SYMBOL	_SEGMENT	_Accumber
ENSG0000(	2	3.2E+07	3.2E+07	282	37	1417886	0.51678	Non_Signif	XDH	152	1.2E-08
ENSG0000(	2	3.2E+07	3.2E+07	300	17	1403266	1.5606	Non_Signif	MEMO1	152	2.1E-11
ENSG0000(	2	3.2E+07	3.2E+07	382	20	1390615	1.6523	Non_Signif	DPY30	152	2.1E-11
ENSG0000(	2	3.2E+07	3.2E+07	223	21	1374335	3.0298	Non_Signif	SPAST	152	2.1E-11
ENSG0000(	2	3.2E+07	3.2E+07	142	17	1348274	2.7724	Non_Signif	SLC30A6	152	2.1E-11
ENSG0000(	2	3.2E+07	3.2E+07	84	11	1389044	2.3152	Non_Signif	NLRC4	152	3.4E-11
ENSG0000(	2	3.3E+07	3.3E+07	78	16	1394543	2.9663	Non_Signif	YIPF4	152	2.1E-11
ENSG0000(	2	3.3E+07	3.3E+07	594	23	1344931	2.0583	Non_Signif	BIRC6	152	2.1E-11
ENSG0000(	2	3.3E+07	3.3E+07	720	47	1410484	2.564	Non_Signif	TTC27	152	2.1E-11
ENSG0000(	2	3.3E+07	3.4E+07	1895	78	1427064	1.1835	Non_Signif	LTBP1	152	4.7E-09
ENSG0000(	2	3.4E+07	3.4E+07	423	66	1353348	2.3786	Non_Signif	RASGRP3	153	2.8E-09
ENSG0000(	2	3.4E+07	3.4E+07	42	10	1395668	0.62902	Non_Signif	FAM98A	153	4.7E-09
ENSG0000(	10	1.2E+08	1.2E+08	257	19	1413917	0.26407	Non_Signif	HSPA12A	1081	2.1E-05
ENSG0000(	10	1.2E+08	1.2E+08	82	16	1356686	2.5246	Non_Signif	ENO4	1081	5.3E-08
ENSG0000(	10	1.2E+08	1.2E+08	302	23	1291231	1.8041	Non_Signif	KIAA1598	1081	4.9E-08
ENSG0000(	10	1.2E+08	1.2E+08	10	3	1153992	1.5693	Non_Signif	VAX1	1081	9.1E-08
ENSG0000(	10	1.2E+08	1.2E+08	70	6	1411837	0.29646	Non_Signif	KCNK18	1081	9.1E-08
ENSG0000(	11	1244296	1283406	177	14	1400444	1.4373	Non_Signif	MUC5B	1096	1.5E-08
ENSG0000(	11	1295601	1330884	117	24	1374890	1.3043	Non_Signif	TOLLIP	1096	2.9E-09
ENSG0000(	11	1411129	1483919	317	31	1304150	2.4386	Non_Signif	BRSK2	1096	2.2E-09
ENSG0000(	11	1490687	1522477	101	10	1399639	3.1667	Non_Signif	MOB2	1096	4.5E-10
ENSG0000(	11	1575274	1593150	60	7	1432984	3.0413	Non_Signif	DUSP8	1096	4.5E-10
ENSG0000(	11	1628795	1629693	6	3	1473741	2.2681	Non_Signif	KRTAP5-3	1096	1.9E-09
ENSG0000(	11	1642188	1643368	4	2	1473563	0.20719	Non_Signif	KRTAP5-4	1096	1.8E-09
ENSG0000(	11	1651033	1652160	5	2	1474097	0.06356	Non_Signif	KRTAP5-5	1096	1.8E-09
ENSG0000(	11	1874200	1913497	189	30	1353292	2.8005	Non_Signif	LSP1	1096	1.2E-08
ENSG0000(	11	2397407	2418649	72	4	913881	-0.48127	Non_Signif	CD81	1096	1.8E-09
ENSG0000(	11	2421718	2425106	12	7	689219	0.13539	Non_Signif	TSSC4	1096	1.2E-08
ENSG0000(	11	2465914	2870339	1295	134	1236330	0.81808	Non_Signif	KCNQ1	1096	1.2E-08
ENSG0000(	20	2.5E+07	2.5E+07	49	6	1434595	-2.0161	Non_Signif	CST7	1634	1.4E-11
ENSG0000(	20	2.5E+07	2.5E+07	81	9	1405374	-0.67948	Non_Signif	APMAP	1634	1.4E-09
ENSG0000(	20	2.5E+07	2.5E+07	129	25	1365562	1.0509	Non_Signif	ACSS1	1634	1.4E-09
ENSG0000(	20	2.5E+07	2.5E+07	34	4	1458735	2.2991	Non_Signif	VSX1	1634	6.8E-12

ENSG0000(	20	2.5E+07	2.5E+07	100	13	1449557	1.1938	Non_Signif	ENTPD6	1634	9.6E-13
ENSG0000(	20	2.5E+07	2.5E+07	175	12	1425046	2.5564	Non_Signif	PYGB	1634	9.6E-13
ENSG0000(	20	2.5E+07	2.5E+07	232	12	1371278	2.0798	Non_Signif	ABHD12	1634	9.6E-13
ENSG0000(	20	2.5E+07	2.5E+07	120	11	1354544	2.3491	Non_Signif	GINS1	1635	1.5E-11
ENSG0000(	20	2.5E+07	2.6E+07	313	19	1378656	2.895	Non_Signif	NINL	1635	9.6E-13
ENSG0000(	20	2.6E+07	2.6E+07	31	5	1397440	2.1904	Non_Signif	NANP	1635	9.6E-13
ENSG0000(	20	2.6E+07	2.6E+07	43	7	1359140	3.166	Non_Signif	ZNF337	1635	7.6E-12
ENSG0000(	20	2.6E+07	2.6E+07	63	9	925406	1.5554	Non_Signif	FAM182B	1635	9.6E-13
ENSG0000(	20	3E+07	3E+07	7	2	208671	-2.1882	Non_Signif	FRG1B	1635	4.3E-16
ENSG0000(	20	3E+07	3E+07	5	2	1462867	1.59	Non_Signif	DEFB115	1635	8.5E-12
ENSG0000(	20	3E+07	3E+07	14	5	1192121	1.057	Non_Signif	DEFB116	1635	9.7E-12
ENSG0000(	20	3E+07	3E+07	7	3	1466105	0.56639	Non_Signif	DEFB118	1635	6.2E-12
ENSG0000(	20	3E+07	3E+07	40	9	1416500	2.7599	Non_Signif	DEFB119	1635	6.2E-12
ENSG0000(	20	3E+07	3E+07	18	4	1465365	2.7923	Non_Signif	DEFB121	1635	2.8E-13
ENSG0000(	20	3E+07	3E+07	24	5	1409979	2.3631	Non_Signif	DEFB123	1635	9E-14
ENSG0000(	20	3E+07	3E+07	18	5	1455803	2.3594	Non_Signif	DEFB124	1635	3.8E-15
ENSG0000(	20	3E+07	3E+07	16	6	1306635	2.2655	Non_Signif	REM1	1635	3.8E-15
ENSG0000(	20	3E+07	3E+07	114	7	1361286	3.8709	5.4E-05	HM13	1635	1.4E-10
ENSG0000(	20	3E+07	3E+07	8	4	1030031	3.3933	0.00035	ID1	1635	1.4E-10
ENSG0000(	20	3E+07	3E+07	14	2	1456132	4.6912	1.4E-06	COX4I2	1635	7.9E-23
ENSG0000(	20	3E+07	3E+07	94	9	1322813	4.8943	4.9E-07	BCL2L1	1635	7.9E-23
ENSG0000(	20	3E+07	3E+07	98	10	1411583	4.8387	6.5E-07	TPX2	1635	3.4E-21
ENSG0000(	20	3E+07	3E+07	43	7	1430985	4.5495	2.7E-06	MYLK2	1635	7.9E-23
ENSG0000(	20	3E+07	3E+07	2	1	1460059	4.3093	8.2E-06	FOXS1	1635	4.3E-22
ENSG0000(	20	3E+07	3E+07	59	18	1335906	3.5976	0.00016	DUSP15	1635	1E-20
ENSG0000(	20	3E+07	3.1E+07	149	24	1400319	1.9801	Non_Signif	TTLL9	1635	1.4E-14
ENSG0000(	20	3.1E+07	3.1E+07	6	2	1327730	0.44204	Non_Signif	PDRG1	1635	3.4E-21
ENSG0000(	20	3.1E+07	3.1E+07	76	21	1325600	-0.73843	Non_Signif	XKR7	1635	4.3E-22
ENSG0000(	20	3.1E+07	3.1E+07	43	10	1389396	0.25176	Non_Signif	CCM2L	1635	4.3E-22
ENSG0000(	20	3.1E+07	3.1E+07	90	13	1372169	1.0627	Non_Signif	HCK	1635	3.7E-10
ENSG0000(	20	3.1E+07	3.1E+07	111	10	1396583	-0.08615	Non_Signif	TM9SF4	1635	4.3E-22
ENSG0000(	20	3.1E+07	3.1E+07	12	3	1175933	-0.07234	Non_Signif	PLAGL2	1635	4.3E-22
ENSG0000(	20	3.1E+07	3.1E+07	34	6	1395129	0.05792	Non_Signif	POFUT1	1635	4.3E-22
ENSG0000(	20	3.1E+07	3.1E+07	184	26	1290667	-0.29214	Non_Signif	C20orf112	1635	1.2E-13
ENSG0000(	20	3.1E+07	3.1E+07	130	10	1423241	3.3712	0.00037	COMMD7	1635	3.7E-21

ENSG0000(	22	5.1E+07	5.1E+07	373	13	1439744	2.4012	Non_Signif	MOV10L1	1702	9.5E-07
ENSG0000(	22	5.1E+07	5.1E+07	26	10	1387646	1.7008	Non_Signif	PANX2	1702	3.5E-08
ENSG0000(	22	5.1E+07	5.1E+07	55	12	1406339	1.5436	Non_Signif	TRABD	1702	3.5E-08
ENSG0000(	22	5.1E+07	5.1E+07	61	12	1314460	1.7784	Non_Signif	SELO	1702	3.5E-08
ENSG0000(	22	5.1E+07	5.1E+07	101	15	1208481	0.71283	Non_Signif	TUBGCP6	1702	3.5E-08
ENSG0000(	22	5.1E+07	5.1E+07	21	6	1301149	-0.28481	Non_Signif	HDAC10	1702	1.6E-06
ENSG0000(	22	5.1E+07	5.1E+07	59	12	1359026	0.49659	Non_Signif	MAPK12	1702	3.5E-08
ENSG0000(	22	5.1E+07	5.1E+07	18	7	1437767	1.4573	Non_Signif	MAPK11	1702	3.5E-08
ENSG0000(	22	5.1E+07	5.1E+07	339	26	1394952	3.5631	0.00018	PPP6R2	1702	1.6E-06
ENSG0000(	22	5.1E+07	5.1E+07	11	5	1463995	-0.14838	Non_Signif	KLHDC7B	1702	3.5E-08

PD_GWAS_TOP_GENE	SIGNIFICANT_IN_BOTH_AND_IN_SEGMENT_OF_INTEREST
FALSE	FALSE
FALSE	FALSE
FALSE	FALSE
FALSE	FALSE
FALSE	FALSE
FALSE	FALSE
FALSE	FALSE
FALSE	FALSE
FALSE	FALSE
FALSE	FALSE
FALSE	FALSE
FALSE	FALSE
FALSE	FALSE
FALSE	FALSE
FALSE	FALSE
FALSE	FALSE
FALSE	FALSE
FALSE	FALSE
FALSE	FALSE
FALSE	FALSE
FALSE	FALSE
FALSE	FALSE
FALSE	FALSE
FALSE	FALSE
FALSE	FALSE
FALSE	FALSE
FALSE	FALSE
FALSE	FALSE
FALSE	FALSE
FALSE	FALSE
FALSE	FALSE
FALSE	FALSE
FALSE	FALSE

FALSE	FALSE
FALSE	FALSE
FALSE	FALSE
FALSE	FALSE
FALSE	FALSE
FALSE	FALSE
FALSE	FALSE
FALSE	FALSE
FALSE	FALSE
FALSE	FALSE
FALSE	FALSE
FALSE	FALSE
FALSE	FALSE
FALSE	FALSE
FALSE	FALSE
FALSE	FALSE
FALSE	FALSE
FALSE	TRUE
FALSE	TRUE
FALSE	TRUE
FALSE	TRUE
FALSE	TRUE
FALSE	TRUE
FALSE	TRUE
FALSE	TRUE
FALSE	FALSE
FALSE	FALSE
FALSE	FALSE
FALSE	FALSE
FALSE	FALSE
FALSE	FALSE
FALSE	FALSE
FALSE	FALSE
FALSE	FALSE
FALSE	TRUE

FALSE	FALSE
FALSE	FALSE
FALSE	FALSE
FALSE	FALSE
FALSE	FALSE
FALSE	FALSE
FALSE	FALSE
FALSE	FALSE
FALSE	FALSE
FALSE	FALSE

Supplementary Table 8. Statistically significant MAGMA results for the PD and the volume of the ICV											
GENE	CHR	START	STOP	NSNPS	NPARAM	N	ZSTAT	P_PD	SYMBOL	_SEGMENT	_Accumber
ENSG0000(	1	2E+08	2E+08	574	55	1411621	2.0249	Non_Signif	NFASC	105	8.8E-09
ENSG0000(	1	2.1E+08	2.1E+08	101	13	1424625	0.94364	Non_Signif	RBBP5	105	1.2E-08
ENSG0000(	1	2.4E+08	2.4E+08	204	22	1180065	0.95562	Non_Signif	CEP170	129	2.7E-11
ENSG0000(	1	2.4E+08	2.4E+08	429	36	1396738	2.2611	Non_Signif	SDCCAG8	129	2.7E-11
ENSG0000(	1	2.4E+08	2.4E+08	524	23	1313904	4.6686	1.5E-06	AKT3	129	1.8E-11
ENSG0000(	3	4.1E+07	4.1E+07	97	11	1393648	2.694	Non_Signif	CTNNB1	306	4.9E-08
ENSG0000(	3	4.1E+07	4.2E+07	2129	39	1403469	0.44865	Non_Signif	ULK4	306	4.9E-08
ENSG0000(	3	5.2E+07	5.2E+07	39	14	1385209	0.36902	Non_Signif	TEX264	311	1.2E-06
ENSG0000(	3	1.4E+08	1.4E+08	177	19	1422567	1.115	Non_Signif	ATP1B3	363	4.1E-15
ENSG0000(	3	1.4E+08	1.4E+08	682	19	1425830	1.4377	Non_Signif	TFDP2	363	4.1E-17
ENSG0000(	3	1.4E+08	1.4E+08	188	20	1390357	0.22791	Non_Signif	XRN1	363	1E-09
ENSG0000(	3	1.4E+08	1.4E+08	276	14	1440470	0.63008	Non_Signif	ATR	363	1E-09
ENSG0000(	3	1.4E+08	1.4E+08	259	17	1425394	0.33813	Non_Signif	PLS1	363	1.7E-08
ENSG0000(	3	1.4E+08	1.4E+08	154	14	1444982	0.93156	Non_Signif	TRPC1	363	3.9E-08
ENSG0000(	3	1.4E+08	1.4E+08	40	12	1332822	-0.65386	Non_Signif	PAQR9	363	2E-08
ENSG0000(	3	1.4E+08	1.4E+08	199	19	1371160	1.4026	Non_Signif	U2SURP	363	2E-08
ENSG0000(	4	1.7E+07	1.8E+07	255	26	1375029	-0.10295	Non_Signif	QDPR	415	2.1E-07
ENSG0000(	4	1.8E+07	1.8E+07	31	7	1459714	0.8503	Non_Signif	CLRN2	415	2.1E-07
ENSG0000(	4	1.8E+07	1.8E+07	79	6	1335804	0.43731	Non_Signif	LAP3	415	2.1E-10
ENSG0000(	4	1.8E+07	1.8E+07	60	6	1390024	1.2879	Non_Signif	MED28	415	7.1E-08
ENSG0000(	4	1.8E+07	1.8E+07	396	16	1361241	1.5545	Non_Signif	FAM184B	415	1.3E-10
ENSG0000(	4	1.8E+07	1.8E+07	13	3	1438014	4.4747	3.8E-06	DCAF16	415	2.2E-10
ENSG0000(	4	1.8E+07	1.8E+07	63	8	1445031	4.7874	8.5E-07	NCAPG	415	2.2E-10
ENSG0000(	4	1.8E+07	1.8E+07	414	16	1435745	5.8985	1.8E-09	LCORL	415	1.2E-10
ENSG0000(	6	3.6E+07	3.6E+07	42	10	1363824	1.1674	Non_Signif	BRPF3	659	1.3E-09
ENSG0000(	6	3.6E+07	3.6E+07	166	14	1414443	-1.6379	Non_Signif	PXT1	659	1.3E-09
ENSG0000(	6	3.6E+07	3.6E+07	120	12	1349697	-1.5069	Non_Signif	KCTD20	659	1.3E-09
ENSG0000(	6	3.6E+07	3.7E+07	162	12	1374856	-1.2564	Non_Signif	STK38	659	1.3E-09
ENSG0000(	6	3.7E+07	3.7E+07	40	5	1071501	-0.93519	Non_Signif	SRSF3	659	1.3E-09
ENSG0000(	6	3.7E+07	3.7E+07	364	56	1418607	1.0206	Non_Signif	CPNE5	659	1.3E-09
ENSG0000(	6	3.7E+07	3.7E+07	82	10	1384855	0.71403	Non_Signif	MTCH1	659	1.3E-09
ENSG0000(	6	3.7E+07	3.7E+07	72	20	1400294	1.6543	Non_Signif	FGD2	659	1.3E-09
ENSG0000(	6	3.7E+07	3.7E+07	7	3	1268980	0.98657	Non_Signif	PIM1	659	1.3E-09

ENSG0000(	6	3.7E+07	3.7E+07	65	13	1374754	-0.75002	Non_Signif	TMEM217	659	1.3E-09
ENSG0000(	6	3.7E+07	3.7E+07	151	13	1397204	0.47867	Non_Signif	TBC1D22B	659	1.3E-09
ENSG0000(	6	3.7E+07	3.7E+07	60	14	1386843	1.2281	Non_Signif	CMTR1	659	1.3E-09
ENSG0000(	6	3.7E+07	3.7E+07	35	10	1446903	1.0918	Non_Signif	CCDC167	659	1.3E-09
ENSG0000(	8	1.3E+08	1.3E+08	279	31	1404337	4.5825	2.3E-06	FAM49B	926	2.6E-13
ENSG0000(	8	1.4E+08	1.4E+08	2227	169	1374205	-0.67426	Non_Signif	TRAPPC9	933	1.5E-08
ENSG0000(	8	1.4E+08	1.4E+08	14	6	994471	-0.67132	Non_Signif	CHRAC1	933	1.7E-07
ENSG0000(	8	1.4E+08	1.4E+08	282	41	1333690	3.1767	Non_Signif	AGO2	933	1.7E-07
ENSG0000(	8	1.4E+08	1.4E+08	296	34	1307774	2.7949	Non_Signif	PTK2	933	1.5E-08
ENSG0000(	8	1.4E+08	1.4E+08	177	35	1255581	-0.13699	Non_Signif	DENND3	933	1.7E-08
ENSG0000(	8	1.4E+08	1.4E+08	361	42	1417321	1.0059	Non_Signif	SLC45A4	933	1.1E-05
ENSG0000(	8	1.4E+08	1.4E+08	7	3	1458527	-0.15111	Non_Signif	RP11-10J2	933	5.9E-07
ENSG0000(	8	1.4E+08	1.4E+08	41	8	1438485	-0.12714	Non_Signif	GPR20	933	1.2E-05
ENSG0000(	8	1.4E+08	1.4E+08	18	6	1291709	1.0965	Non_Signif	AC138647.	933	1.4E-05
ENSG0000(	10	2.2E+07	2.2E+07	8	3	1339917	1.4955	Non_Signif	CASC10	1025	3.9E-12
ENSG0000(	10	2.2E+07	2.2E+07	9	4	1451513	1.8475	Non_Signif	SKIDA1	1025	3.9E-12
ENSG0000(	10	2.2E+07	2.2E+07	196	33	1340390	2.1485	Non_Signif	MLLT10	1025	3.9E-12
ENSG0000(	10	2.2E+07	2.2E+07	399	25	1388404	1.0401	Non_Signif	DNAJC1	1025	6.8E-11
ENSG0000(	10	2.3E+07	2.3E+07	2	1	1016923	-0.74733	Non_Signif	COMMD3	1025	3.9E-12
ENSG0000(	10	2.3E+07	2.3E+07	5	2	1150581	-0.21565	Non_Signif	COMMD3-I	1025	3.9E-12
ENSG0000(	10	2.3E+07	2.3E+07	4	2	1267533	-0.79874	Non_Signif	BMI1	1025	3.9E-12
ENSG0000(	10	2.3E+07	2.3E+07	158	12	1236721	-0.28279	Non_Signif	SPAG6	1025	3.9E-12
ENSG0000(	10	9E+07	9E+07	142	33	1368828	2.7409	Non_Signif	PTEN	1065	8E-11
ENSG0000(	10	9E+07	9E+07	685	40	1416683	0.24528	Non_Signif	RNLS	1065	8E-11
ENSG0000(	10	9E+07	9E+07	25	8	1413810	-0.27601	Non_Signif	LIPJ	1065	8E-11
ENSG0000(	10	9.1E+07	9.1E+07	104	13	1456794	-1.7856	Non_Signif	FAS	1065	3.5E-06
ENSG0000(	11	1244296	1283406	177	14	1400444	1.4373	Non_Signif	MUC5B	1096	3.8E-12
ENSG0000(	11	1295601	1330884	117	24	1374890	1.3043	Non_Signif	TOLLIP	1096	5.4E-14
ENSG0000(	11	1411129	1483919	317	31	1304150	2.4386	Non_Signif	BRSK2	1096	5.4E-14
ENSG0000(	11	1490687	1522477	101	10	1399639	3.1667	Non_Signif	MOB2	1096	1.5E-14
ENSG0000(	11	1575274	1593150	60	7	1432984	3.0413	Non_Signif	DUSP8	1096	1.5E-14
ENSG0000(	11	1628795	1629693	6	3	1473741	2.2681	Non_Signif	KRTAP5-3	1096	6.2E-14
ENSG0000(	11	1642188	1643368	4	2	1473563	0.20719	Non_Signif	KRTAP5-4	1096	6.2E-14
ENSG0000(	11	1651033	1652160	5	2	1474097	0.06356	Non_Signif	KRTAP5-5	1096	6.2E-14
ENSG0000(	11	1860219	1862910	6	3	914330	1.5772	Non_Signif	TNNI2	1096	2E-09

ENSG0000(	11	1874200	1913497	189	30	1353292	2.8005	Non_Signif	LSP1	1096	6E-13
ENSG0000(	11	1968508	2005752	154	22	1332484	-0.73529	Non_Signif	MRPL23	1096	2E-09
ENSG0000(	11	2150342	2170833	56	16	1325027	1.5979	Non_Signif	IGF2	1096	3.2E-10
ENSG0000(	11	2185159	2193107	21	5	1274161	1.1311	Non_Signif	TH	1096	2E-09
ENSG0000(	11	2397407	2418649	72	4	913881	-0.48127	Non_Signif	CD81	1096	5.4E-14
ENSG0000(	11	2421718	2425106	12	7	689219	0.13539	Non_Signif	TSSC4	1096	6E-13
ENSG0000(	11	2465914	2870339	1295	134	1236330	0.81808	Non_Signif	KCNQ1	1096	6E-13
ENSG0000(	12	4.9E+07	4.9E+07	25	12	1409474	2.2914	Non_Signif	CACNB3	1209	1.5E-08
ENSG0000(	12	4.9E+07	4.9E+07	38	8	1327312	0.88177	Non_Signif	DDX23	1209	3.8E-08
ENSG0000(	12	4.9E+07	4.9E+07	14	5	1425169	1.3575	Non_Signif	RND1	1209	1.7E-08
ENSG0000(	12	4.9E+07	4.9E+07	69	7	1403504	-0.13019	Non_Signif	CCDC65	1209	1.1E-08
ENSG0000(	12	4.9E+07	4.9E+07	9	3	1323445	0.36111	Non_Signif	FKBP11	1209	1.1E-08
ENSG0000(	12	4.9E+07	4.9E+07	6	3	1437621	3.6117	0.00015	DDN	1209	1.5E-08
ENSG0000(	12	4.9E+07	4.9E+07	14	3	1384575	3.012	Non_Signif	PRKAG1	1209	1.5E-08
ENSG0000(	12	4.9E+07	4.9E+07	35	7	1409574	2.9627	Non_Signif	KMT2D	1209	1.1E-08
ENSG0000(	12	4.9E+07	4.9E+07	5	1	1451444	1.6198	Non_Signif	RHEBL1	1209	1.1E-08
ENSG0000(	12	4.9E+07	4.9E+07	6	3	1437767	1.6453	Non_Signif	DHH	1209	1.5E-08
ENSG0000(	12	4.9E+07	5E+07	13	3	1412183	3.3624	Non_Signif	LMBR1L	1209	1.5E-08
ENSG0000(	12	5E+07	5E+07	5	1	1381702	2.6997	Non_Signif	TUBA1B	1209	1.7E-07
ENSG0000(	12	5E+07	5E+07	5	2	1449691	2.7007	Non_Signif	TUBA1A	1209	1.1E-08
ENSG0000(	12	5E+07	5E+07	159	29	1385674	1.6661	Non_Signif	TUBA1C	1209	1.1E-08
ENSG0000(	12	5E+07	5E+07	17	5	1439947	0.2758	Non_Signif	TROAP	1209	1.5E-08
ENSG0000(	12	5E+07	5E+07	15	5	1204158	0.43484	Non_Signif	C1QL4	1209	1.7E-08
ENSG0000(	12	5E+07	5E+07	29	3	1400895	0.44589	Non_Signif	DNAJC22	1209	1.7E-08
ENSG0000(	12	5E+07	5E+07	333	21	1387887	2.3373	Non_Signif	SPATS2	1209	1.7E-08
ENSG0000(	12	5E+07	5E+07	129	15	1328757	1.3525	Non_Signif	NCKAP5L	1209	1.7E-08
ENSG0000(	12	5.3E+07	5.3E+07	39	8	1421673	-0.96135	Non_Signif	KRT77	1211	2.2E-13
ENSG0000(	12	5.3E+07	5.3E+07	44	12	1361256	1.1047	Non_Signif	TENC1	1211	1.5E-08
ENSG0000(	12	5.3E+07	5.3E+07	30	9	1379893	2.4424	Non_Signif	SPRYD3	1211	2.3E-14
ENSG0000(	12	5.3E+07	5.3E+07	8	5	1120556	-1.3635	Non_Signif	IGFBP6	1211	2.1E-14
ENSG0000(	12	5.3E+07	5.4E+07	92	9	1444783	-0.59439	Non_Signif	SOAT2	1211	2.1E-14
ENSG0000(	12	5.4E+07	5.4E+07	34	5	1305629	-0.59236	Non_Signif	CSAD	1211	4.6E-12
ENSG0000(	12	5.4E+07	5.4E+07	25	5	1393273	-0.1139	Non_Signif	ZNF740	1211	4.6E-12
ENSG0000(	12	5.4E+07	5.4E+07	38	11	1316271	0.40859	Non_Signif	ITGB7	1211	4.6E-12
ENSG0000(	12	5.4E+07	5.4E+07	34	9	1348827	-0.32066	Non_Signif	RARG	1211	2.9E-15

ENSG0000(	12	5.4E+07	5.4E+07	10	3	1316574	0.76162	Non_Signif	MFSD5	1211	2.3E-15
ENSG0000(	12	5.4E+07	5.4E+07	10	2	1317942	-0.33407	Non_Signif	PFDN5	1211	2.3E-15
ENSG0000(	12	5.4E+07	5.4E+07	7	2	1457899	-0.76331	Non_Signif	C12orf10	1211	4.6E-12
ENSG0000(	12	5.4E+07	5.4E+07	26	4	1373762	-0.8614	Non_Signif	AAAS	1211	2.3E-15
ENSG0000(	12	5.4E+07	5.4E+07	32	7	1410491	1.4812	Non_Signif	SP7	1211	2E-15
ENSG0000(	12	5.4E+07	5.4E+07	69	9	1388637	2.6803	Non_Signif	SP1	1211	2E-15
ENSG0000(	12	5.4E+07	5.4E+07	13	1	1474097	3.2758	Non_Signif	AMHR2	1211	2.3E-15
ENSG0000(	12	5.4E+07	5.4E+07	6	3	1343406	2.5152	Non_Signif	PRR13	1211	2E-15
ENSG0000(	12	5.4E+07	5.4E+07	38	10	1316164	2.2846	Non_Signif	PCBP2	1211	2E-15
ENSG0000(	12	5.4E+07	5.4E+07	16	10	1281068	2.6253	Non_Signif	MAP3K12	1211	2E-15
ENSG0000(	12	5.4E+07	5.4E+07	2	1	1418534	2.3611	Non_Signif	TARBP2	1211	2E-15
ENSG0000(	12	5.4E+07	5.4E+07	207	18	1426413	2.624	Non_Signif	RP11-793H	1211	2.3E-15
ENSG0000(	12	5.4E+07	5.4E+07	1	1	1474097	0.99159	Non_Signif	NPFF	1211	2.3E-15
ENSG0000(	12	5.4E+07	5.4E+07	206	18	1426181	2.6219	Non_Signif	ATF7	1211	2.3E-15
ENSG0000(	12	5.4E+07	5.4E+07	115	9	1435540	2.9658	Non_Signif	ATP5G2	1211	2E-15
ENSG0000(	12	5.4E+07	5.4E+07	46	10	1351137	2.1266	Non_Signif	CALCOCO1	1211	1.5E-13
ENSG0000(	12	5.4E+07	5.4E+07	36	14	1050909	0.71966	Non_Signif	HOXC4	1211	1.5E-13
ENSG0000(	12	5.4E+07	5.4E+07	3	2	910005	-0.83452	Non_Signif	HOXC5	1211	1.5E-08
ENSG0000(	12	5.4E+07	5.4E+07	4	2	1382406	1.5394	Non_Signif	HOXC4	1211	1.5E-13
ENSG0000(	12	5.5E+07	5.5E+07	50	7	1438562	-0.66249	Non_Signif	SMUG1	1211	1.5E-13
ENSG0000(	12	5.5E+07	5.5E+07	42	8	1421550	1.4153	Non_Signif	CBX5	1211	1.3E-11
ENSG0000(	12	5.5E+07	5.5E+07	7	3	1384297	1.0703	Non_Signif	HNRNPA1	1211	1.3E-11
ENSG0000(	12	5.5E+07	5.5E+07	30	7	1386351	2.0003	Non_Signif	ZNF385A	1211	2E-15
ENSG0000(	17	1.5E+07	1.6E+07	80	15	1125842	-0.16122	Non_Signif	CDRT1	1496	6.2E-07
ENSG0000(	17	1.6E+07	1.6E+07	56	12	1036397	-0.86257	Non_Signif	ZNF286A	1496	1.5E-06
ENSG0000(	17	1.6E+07	1.6E+07	73	15	1084315	-0.9095	Non_Signif	ZNF286A	1496	1.5E-06
ENSG0000(	17	1.6E+07	1.6E+07	23	5	1149838	-0.14864	Non_Signif	TBC1D26	1496	6.2E-07
ENSG0000(	17	1.6E+07	1.6E+07	54	11	1160798	2.891	Non_Signif	ADORA2B	1496	8.6E-10
ENSG0000(	17	1.6E+07	1.6E+07	51	5	1392490	4.778	8.9E-07	ZSWIM7	1496	8.6E-10
ENSG0000(	17	1.6E+07	1.6E+07	102	9	1393820	4.3063	8.3E-06	TTC19	1496	8.6E-10
ENSG0000(	17	1.6E+07	1.6E+07	321	14	1336264	5.1432	1.4E-07	NCOR1	1496	8.6E-10
ENSG0000(	17	1.6E+07	1.6E+07	1	1	1474097	5.0673	2E-07	AC002553.	1496	4.1E-09
ENSG0000(	17	1.6E+07	1.6E+07	259	12	1384394	4.6734	1.5E-06	PIGL	1496	2.1E-09
ENSG0000(	17	1.6E+07	1.6E+07	18	5	1357684	4.7695	9.2E-07	CENPV	1496	8.6E-10
ENSG0000(	17	1.6E+07	1.6E+07	64	10	1393404	2.3393	Non_Signif	TRPV2	1496	8.6E-10

ENSG0000(	17	2.8E+07	2.8E+07	292	16	1340725	1.9198	Non_Signif	TAOK1	1500	1.1E-12
ENSG0000(	17	2.8E+07	2.8E+07	11	7	1084273	2.1366	Non_Signif	ABHD15	1500	1.1E-12
ENSG0000(	17	2.8E+07	2.8E+07	32	9	1401933	3.2689	Non_Signif	GIT1	1500	6.3E-12
ENSG0000(	17	2.8E+07	2.8E+07	10	4	1271709	-0.25925	Non_Signif	CORO6	1500	6.3E-12
ENSG0000(	17	4.3E+07	4.3E+07	1	1	1353390	2.1019	Non_Signif	FZD2	1508	2E-33
ENSG0000(	17	4.3E+07	4.3E+07	78	18	1382266	3.8005	7.2E-05	DBF4B	1508	6.2E-35
ENSG0000(	17	4.3E+07	4.3E+07	52	11	1387543	4.5355	2.9E-06	GJC1	1508	4.2E-35
ENSG0000(	17	4.3E+07	4.3E+07	14	6	1405133	5.2409	8E-08	HIGD1B	1508	9E-34
ENSG0000(	17	4.3E+07	4.3E+07	144	17	1452847	3.852	5.9E-05	EFTUD2	1508	9E-34
ENSG0000(	17	4.3E+07	4.3E+07	19	4	1460549	3.4996	0.00023	CCDC103	1508	9E-34
ENSG0000(	17	4.3E+07	4.3E+07	16	3	1465687	3.3883	Non_Signif	FAM187A	1508	9E-34
ENSG0000(	17	4.3E+07	4.3E+07	42	11	1455308	3.6851	0.00011	GFAP	1508	7.9E-35
ENSG0000(	17	4.3E+07	4.3E+07	136	16	1430326	0.92897	Non_Signif	NMT1	1509	2.6E-35
ENSG0000(	17	4.3E+07	4.3E+07	12	5	1302564	3.8316	6.4E-05	HEXIM2	1509	9E-34
ENSG0000(	17	4.3E+07	4.3E+07	50	8	1430881	1.8861	Non_Signif	FMNL1	1509	2.6E-35
ENSG0000(	17	4.3E+07	4.3E+07	24	4	1474097	2.0315	Non_Signif	SPATA32	1509	9E-34
ENSG0000(	17	4.3E+07	4.4E+07	36	7	1287551	4.7201	1.2E-06	ARHGAP27	1509	2.6E-35
ENSG0000(	17	4.4E+07	4.4E+07	37	12	1186894	4.9481	3.8E-07	PLEKHM1	1509	2.6E-35
ENSG0000(	17	4.4E+07	4.4E+07	227	43	1318454	7.795	3.2E-15	CRHR1	1509	1.1E-41
ENSG0000(	17	4.4E+07	4.4E+07	4	2	1444713	3.6945	0.00011	SPPL2C	1509	1.1E-41
ENSG0000(	17	4.4E+07	4.4E+07	148	36	1266167	6.7583	7E-12	MAPT	1509	1.1E-41
ENSG0000(	17	4.4E+07	4.4E+07	173	25	1239074	7.6079	1.4E-14	KANSL1	1509	1.1E-41
ENSG0000(	17	4.4E+07	4.4E+07	8	3	139582	1.6303	Non_Signif	ARL17B	1509	1.1E-41
ENSG0000(	17	4.4E+07	4.4E+07	1	1	27693	0.56658	Non_Signif	LRRC37A	1509	1.1E-41
ENSG0000(	17	4.5E+07	4.5E+07	2	1	26625	2.9655	Non_Signif	LRRC37A2	1509	1.1E-41
ENSG0000(	17	4.5E+07	4.5E+07	2	1	26625	2.9655	Non_Signif	ARL17A	1509	1.1E-41
ENSG0000(	17	4.5E+07	4.5E+07	57	9	1154938	6.1094	5E-10	NSF	1509	1.1E-41
ENSG0000(	17	4.5E+07	4.5E+07	109	33	1256397	8.2924	5.6E-17	WNT3	1509	1.1E-41
ENSG0000(	17	4.5E+07	4.6E+07	314	15	1415727	0.7689	Non_Signif	EFCAB13	1509	1.7E-35
ENSG0000(	19	3538259	3574288	118	31	985534	1.0309	Non_Signif	MFSD12	1581	1.7E-12
ENSG0000(	19	3572775	3579086	8	4	626534	0.56108	Non_Signif	HMG20B	1581	1.7E-12
ENSG0000(	19	3610639	3626813	79	11	1319467	-0.20122	Non_Signif	CACTIN	1581	1.7E-12
ENSG0000(	19	3630181	3700477	192	28	1248718	-0.38492	Non_Signif	PIP5K1C	1581	1.7E-12
ENSG0000(	19	3708107	3750811	164	31	1260382	1.7957	Non_Signif	TJP3	1581	1.7E-12
ENSG0000(	19	3769087	3772233	9	2	1351835	-0.62168	Non_Signif	RAX2	1581	5.6E-10

ENSG0000(	19	3879862	3928077	139	35	1174534	-0.87268	Non_Signif	ATCAY	1581	1.7E-12
ENSG0000(	19	3958451	3971121	24	4	1435742	-0.48036	Non_Signif	DAPK3	1581	1.7E-12
ENSG0000(	19	3976054	3985467	29	9	1261875	0.07699	Non_Signif	EEF2	1581	1.7E-12
ENSG0000(	19	4007644	4039384	109	12	1373327	2.2006	Non_Signif	PIAS4	1581	2.6E-10
ENSG0000(	19	4041127	4043154	10	4	1322514	1.512	Non_Signif	AC016586.	1581	1.7E-12
ENSG0000(	19	4044362	4066943	44	9	1284355	3.4882	0.00024	ZBTB7A	1581	1.7E-12
ENSG0000(	19	4090319	4124126	86	16	1330162	3.9981	3.2E-05	MAP2K2	1581	1.7E-12
ENSG0000(	19	4174106	4182601	21	7	1172204	0.28138	Non_Signif	SIRT6	1581	1.7E-12
ENSG0000(	19	4183351	4224811	147	25	1260302	-0.91726	Non_Signif	ANKRD24	1581	1.7E-12
ENSG0000(	19	4229495	4237528	36	4	1380322	-1.1184	Non_Signif	EBI3	1581	1.7E-12
ENSG0000(	19	4247076	4269087	76	5	1394212	-0.49537	Non_Signif	CCDC94	1581	1.7E-12
ENSG0000(	19	4324040	4342783	68	11	1400289	0.76007	Non_Signif	STAP2	1581	1.7E-12
ENSG0000(	19	4343524	4360083	55	14	1221345	0.05707	Non_Signif	MPND	1581	1.7E-12
ENSG0000(	19	7112266	7294045	748	82	1303986	3.2521	Non_Signif	INSR	1584	1.2E-08
ENSG0000(	19	7459999	7537363	230	26	1288444	-0.4426	Non_Signif	ARHGEF18	1584	9.8E-05
ENSG0000(	19	7793843	7798792	21	6	846092	-0.84362	Non_Signif	CLEC4G	1584	1.2E-08
ENSG0000(	20	4.8E+07	4.8E+07	184	17	1368788	3.0205	Non_Signif	B4GALT5	1646	6.3E-09
ENSG0000(	20	4.9E+07	4.9E+07	70	9	1400143	0.78894	Non_Signif	UBE2V1	1646	4.1E-09
ENSG0000(	20	4.9E+07	4.9E+07	125	15	1401989	0.7672	Non_Signif	TMEM189-	1646	4.1E-09
ENSG0000(	20	4.9E+07	4.9E+07	125	15	1401989	0.7672	Non_Signif	TMEM189	1646	4.1E-09
ENSG0000(	20	4.9E+07	4.9E+07	1	1	1474097	-0.82003	Non_Signif	CEBPB	1646	6.8E-09
ENSG0000(	20	4.9E+07	4.9E+07	183	25	1395819	2.3198	Non_Signif	PTPN1	1646	4.1E-09
ENSG0000(	20	4.9E+07	4.9E+07	337	48	1286977	2.467	Non_Signif	FAM65C	1646	4.1E-09

PD_GWAS_TOP_GENE	SIGNIFICANT_IN_BOTH_AND_IN_SEGMENT_OF_INTEREST
FALSE	FALSE
FALSE	FALSE
FALSE	FALSE
FALSE	FALSE
FALSE	TRUE
FALSE	FALSE
FALSE	FALSE
FALSE	FALSE
FALSE	FALSE
FALSE	FALSE
FALSE	FALSE
FALSE	FALSE
FALSE	FALSE
FALSE	FALSE
FALSE	FALSE
FALSE	FALSE
FALSE	FALSE
FALSE	FALSE
FALSE	FALSE
FALSE	FALSE
FALSE	FALSE
FALSE	TRUE
FALSE	TRUE
FALSE	TRUE
FALSE	FALSE
FALSE	FALSE
FALSE	FALSE
FALSE	FALSE
FALSE	FALSE
FALSE	FALSE
FALSE	FALSE
FALSE	FALSE
FALSE	FALSE

FALSE	FALSE
FALSE	FALSE
FALSE	FALSE
FALSE	FALSE
FALSE	TRUE
FALSE	FALSE
FALSE	FALSE
FALSE	FALSE
FALSE	FALSE
FALSE	FALSE
FALSE	FALSE
FALSE	FALSE
FALSE	FALSE
FALSE	FALSE
FALSE	FALSE
FALSE	FALSE
FALSE	FALSE
FALSE	FALSE
FALSE	FALSE
FALSE	FALSE
FALSE	FALSE
FALSE	FALSE
FALSE	FALSE
FALSE	FALSE
FALSE	FALSE
FALSE	FALSE
FALSE	FALSE
FALSE	FALSE
FALSE	FALSE
FALSE	FALSE
FALSE	FALSE
FALSE	FALSE
FALSE	FALSE
FALSE	FALSE
FALSE	FALSE

FALSE	FALSE
FALSE	FALSE
FALSE	FALSE
FALSE	FALSE
FALSE	FALSE
FALSE	FALSE
FALSE	FALSE
FALSE	FALSE
FALSE	FALSE
FALSE	FALSE
FALSE	FALSE
FALSE	FALSE
FALSE	TRUE
FALSE	FALSE
FALSE	FALSE
FALSE	FALSE
FALSE	FALSE
FALSE	FALSE
FALSE	FALSE
FALSE	FALSE
FALSE	FALSE
FALSE	FALSE
FALSE	FALSE
FALSE	FALSE
FALSE	FALSE
FALSE	FALSE
FALSE	FALSE
FALSE	FALSE
FALSE	FALSE
FALSE	FALSE
FALSE	FALSE
FALSE	FALSE
FALSE	FALSE
FALSE	FALSE
FALSE	FALSE

FALSE	FALSE
FALSE	FALSE
FALSE	FALSE
FALSE	FALSE
FALSE	FALSE
FALSE	FALSE
FALSE	FALSE
FALSE	FALSE
FALSE	FALSE
FALSE	FALSE
FALSE	FALSE
FALSE	FALSE
FALSE	FALSE
FALSE	FALSE
FALSE	FALSE
FALSE	FALSE
FALSE	FALSE
FALSE	FALSE
FALSE	FALSE
FALSE	FALSE
FALSE	FALSE
FALSE	FALSE
FALSE	FALSE
FALSE	FALSE
FALSE	FALSE
FALSE	FALSE
FALSE	FALSE
FALSE	FALSE
FALSE	TRUE
FALSE	TRUE
FALSE	TRUE
FALSE	TRUE
FALSE	TRUE
FALSE	TRUE
FALSE	FALSE

FALSE	FALSE
FALSE	FALSE
FALSE	FALSE
FALSE	FALSE
FALSE	FALSE
FALSE	FALSE
FALSE	FALSE
FALSE	FALSE
FALSE	FALSE
FALSE	FALSE
FALSE	FALSE
FALSE	FALSE
FALSE	FALSE
FALSE	TRUE
FALSE	FALSE
FALSE	FALSE
FALSE	TRUE
FALSE	TRUE
TRUE	TRUE
FALSE	TRUE
FALSE	TRUE
FALSE	TRUE
FALSE	FALSE
FALSE	FALSE
FALSE	FALSE
FALSE	FALSE
FALSE	TRUE
FALSE	TRUE
FALSE	FALSE
FALSE	FALSE
FALSE	FALSE
FALSE	FALSE
FALSE	FALSE
FALSE	FALSE
FALSE	FALSE

FALSE	FALSE
FALSE	FALSE
FALSE	FALSE
FALSE	FALSE
FALSE	FALSE
FALSE	FALSE
FALSE	FALSE
FALSE	FALSE
FALSE	FALSE
FALSE	FALSE
FALSE	FALSE
FALSE	FALSE
FALSE	FALSE
FALSE	FALSE
FALSE	FALSE
FALSE	FALSE
FALSE	FALSE
FALSE	FALSE
FALSE	FALSE
FALSE	FALSE
FALSE	FALSE
FALSE	FALSE
FALSE	FALSE

Supplementary Table 9. Statistically significant MAGMA results for the PD and the volume of th				
GENE	CHR	START	STOP	NSNPS
ENSG0000005428	1	2.43E+08	2.44E+08	429
ENSG0000011702	1	2.44E+08	2.44E+08	524
ENSG0000016295	2	32090129	32236299	300
ENSG0000016296	2	32092878	32264881	382
ENSG0000002157	2	32288680	32382706	223
ENSG0000015268	2	32390933	32449448	142
ENSG0000009110	2	32449522	32490923	84
ENSG0000011982	2	32502979	32541663	78
ENSG0000011576	2	32582096	32843966	594
ENSG0000001869	2	32853099	33046118	720
ENSG0000004932	2	33172039	33624576	1895
ENSG0000015268	2	33661391	33789817	423
ENSG0000011981	2	33808725	33824449	42
ENSG0000015300	5	64013971	64064512	74
ENSG0000015301	5	64064757	64314590	452
ENSG0000004919	5	64444563	64777747	750
ENSG0000012321	5	64813593	64858998	146
ENSG0000011359	5	64859063	64883376	67
ENSG0000011359	5	64885507	64921802	113
ENSG0000011359	5	64920543	64962060	117
ENSG0000025325	5	64920592	64926718	16
ENSG0000019786	5	64961755	65018862	98
ENSG0000012321	5	65018023	65167553	421
ENSG0000011285	5	65222303	65378377	183
ENSG0000012072	5	1.39E+08	1.39E+08	26
ENSG0000022867	5	1.39E+08	1.39E+08	4
ENSG0000017046	5	1.39E+08	1.39E+08	8
ENSG0000017160	5	1.39E+08	1.39E+08	65
ENSG0000015845	5	1.39E+08	1.39E+08	359
ENSG0000016763	8	1.41E+08	1.41E+08	2227
ENSG0000010447	8	1.42E+08	1.42E+08	14
ENSG0000012390	8	1.42E+08	1.42E+08	282
ENSG0000016939	8	1.42E+08	1.42E+08	296
ENSG0000010533	8	1.42E+08	1.42E+08	177
ENSG0000002256	8	1.42E+08	1.42E+08	361
ENSG0000025401	8	1.42E+08	1.42E+08	7
ENSG0000020488	8	1.42E+08	1.42E+08	41
ENSG0000022649	8	1.43E+08	1.43E+08	18
ENSG0000021518	11	1151580	1222364	97
ENSG0000011798	11	1244296	1283406	177
ENSG0000007890	11	1295601	1330884	117
ENSG0000017467	11	1411129	1483919	317
ENSG0000018220	11	1490687	1522477	101
ENSG0000018454	11	1575274	1593150	60
ENSG0000019622	11	1628795	1629693	6
ENSG0000024159	11	1642188	1643368	4
ENSG0000018594	11	1651033	1652160	5
ENSG0000013059	11	1860219	1862910	6
ENSG0000013059	11	1874200	1913497	189
ENSG0000021402	11	1968508	2005752	154

ENSG0000016724	11	2150342	2170833	56
ENSG0000012996	11	2153768	2182439	80
ENSG0000018017	11	2185159	2193107	21
ENSG0000011065	11	2397407	2418649	72
ENSG0000018428	11	2421718	2425106	12
ENSG0000005391	11	2465914	2870339	1295
ENSG0000019671	17	29421945	29709134	354
ENSG0000012686	17	29599031	29624557	28
ENSG0000018586	17	29630784	29641130	14
ENSG0000026511	17	29632231	29645849	18
ENSG0000017299	17	43100708	43138473	81
ENSG0000013644	17	43128978	43186384	136
ENSG0000016171	17	43186335	43210721	75
ENSG0000018151	17	43209967	43221548	19
ENSG0000018683	17	43224684	43229468	7
ENSG0000016851	17	43238067	43247407	12
ENSG0000018492	17	43298811	43324687	50
ENSG0000018436	17	43331760	43339479	24
ENSG0000015931	17	43471275	43511787	36
ENSG0000022519	17	43513266	43568115	37
ENSG0000012008	17	43699267	43913194	227
ENSG0000018529	17	43922256	43924438	4
ENSG0000018686	17	43971748	44105700	148
ENSG0000012007	17	44107282	44302733	173
ENSG0000022869	17	44352150	44439130	8
ENSG0000017668	17	44370099	44415160	1
ENSG0000023808	17	44588877	44633016	2
ENSG0000018582	17	44594068	44657088	2
ENSG0000007396	17	44668035	44834830	57
ENSG0000010837	17	44839872	44910520	109
ENSG0000017885	17	45400656	45518678	314
ENSG0000017110	19	7112266	7294045	748
ENSG0000018256	19	7793843	7798792	21

e ventral diencephalon

NPARAM	N	ZSTAT	P_PD	SYMBOL
36	1396738	2.2611	Non_Significant	SDCCAG8
23	1313904	4.6686	1.52E-06	AKT3
17	1403266	1.5606	Non_Significant	MEMO1
20	1390615	1.6523	Non_Significant	DPY30
21	1374335	3.0298	Non_Significant	SPAST
17	1348274	2.7724	Non_Significant	SLC30A6
11	1389044	2.3152	Non_Significant	NLRC4
16	1394543	2.9663	Non_Significant	YIPF4
23	1344931	2.0583	Non_Significant	BIRC6
47	1410484	2.564	Non_Significant	TTC27
78	1427064	1.1835	Non_Significant	LTBP1
66	1353348	2.3786	Non_Significant	RASGRP3
10	1395668	0.62902	Non_Significant	FAM98A
8	1356729	1.1372	Non_Significant	SREK1IP1
23	1410242	1.1395	Non_Significant	CWC27
42	1421304	1.2298	Non_Significant	ADAMTS6
12	1427851	2.1066	Non_Significant	CENPK
8	1450309	1.9521	Non_Significant	PPWD1
11	1420773	2.205	Non_Significant	TRIM23
10	1447649	1.8195	Non_Significant	TRAPPC13
4	1469855	2.1023	Non_Significant	CTC-534A2.2
16	1375807	1.7663	Non_Significant	SGTB
42	1408936	0.52104	Non_Significant	NLN
22	1349411	1.6989	Non_Significant	ERBB2IP
8	1396456	2.0201	Non_Significant	PAIP2
2	1226255	1.9923	Non_Significant	PROB1
2	1333090	1.1593	Non_Significant	SPATA24
11	1307721	0.9504	Non_Significant	CXXC5
36	1392318	-0.83794	Non_Significant	NRG2
169	1374205	-0.67426	Non_Significant	TRAPPC9
6	994471	-0.67132	Non_Significant	CHRAC1
41	1333690	3.1767	Non_Significant	Aug2
34	1307774	2.7949	Non_Significant	PTK2
35	1255581	-0.13699	Non_Significant	DENND3
42	1417321	1.0059	Non_Significant	SLC45A4
3	1458527	-0.15111	Non_Significant	RP11-10J21.3
8	1438485	-0.12714	Non_Significant	GPR20
6	1291709	1.0965	Non_Significant	AC138647.1
11	1366617	1.9163	Non_Significant	MUC5AC
14	1400444	1.4373	Non_Significant	MUC5B
24	1374890	1.3043	Non_Significant	TOLLIP
31	1304150	2.4386	Non_Significant	BRSK2
10	1399639	3.1667	Non_Significant	MOB2
7	1432984	3.0413	Non_Significant	DUSP8
3	1473741	2.2681	Non_Significant	KRTAP5-3
2	1473563	0.20719	Non_Significant	KRTAP5-4
2	1474097	0.06356	Non_Significant	KRTAP5-5
3	914330	1.5772	Non_Significant	TNNI2
30	1353292	2.8005	Non_Significant	LSP1
22	1332484	-0.73529	Non_Significant	MRPL23

16	1325027	1.5979	Non_Significant	IGF2
20	1318773	2.2291	Non_Significant	INS-IGF2
5	1274161	1.1311	Non_Significant	TH
4	913881	-0.48127	Non_Significant	CD81
7	689219	0.13539	Non_Significant	TSSC4
134	1236330	0.81808	Non_Significant	KCNQ1
10	1362579	1.3468	Non_Significant	NF1
4	1406694	1.851	Non_Significant	OMG
2	1385404	0.52265	Non_Significant	EVI2B
2	1328537	0.39426	Non_Significant	CTD-2370N5.3
14	1441040	1.4974	Non_Significant	DCAKD
16	1430326	0.92897	Non_Significant	NMT1
20	1347976	3.4451	0.000285	PLCD3
7	1338149	3.8163	6.77E-05	ACBD4
3	1200483	2.9026	Non_Significant	HEXIM1
5	1302564	3.8316	6.37E-05	HEXIM2
8	1430881	1.8861	Non_Significant	FMNL1
4	1474097	2.0315	Non_Significant	SPATA32
7	1287551	4.7201	1.18E-06	ARHGAP27
12	1186894	4.9481	3.75E-07	PLEKHM1
43	1318454	7.795	3.22E-15	CRHR1
2	1444713	3.6945	0.00011	SPPL2C
36	1266167	6.7583	6.98E-12	MAPT
25	1239074	7.6079	1.39E-14	KANSL1
3	139582	1.6303	Non_Significant	ARL17B
1	27693	0.56658	Non_Significant	LRRC37A
1	26625	2.9655	Non_Significant	LRRC37A2
1	26625	2.9655	Non_Significant	ARL17A
9	1154938	6.1094	5.00E-10	NSF
33	1256397	8.2924	5.55E-17	WNT3
15	1415727	0.7689	Non_Significant	EFCAB13
82	1303986	3.2521	0.000573	INSR
6	846092	-0.84362	Non_Significant	CLEC4G

MIC_SEGMENT_NU	P_Accumbens	D_GWAS_TOP_GEN	T_IN_BOTH_AND_IN_SEGMENT_O
129	1.21E-09	FALSE	FALSE
129	1.21E-09	FALSE	TRUE
152	1.15E-09	FALSE	FALSE
152	9.07E-09	FALSE	FALSE
152	9.07E-09	FALSE	FALSE
152	6.29E-13	FALSE	FALSE
152	7.45E-09	FALSE	FALSE
152	6.29E-13	FALSE	FALSE
152	1.15E-09	FALSE	FALSE
152	6.42E-14	FALSE	FALSE
152	7.85E-13	FALSE	FALSE
153	6.42E-14	FALSE	FALSE
153	7.85E-13	FALSE	FALSE
560	2.75E-23	FALSE	FALSE
560	2.75E-23	FALSE	FALSE
560	4.14E-22	FALSE	FALSE
560	1.60E-24	FALSE	FALSE
560	1.60E-24	FALSE	FALSE
560	1.60E-24	FALSE	FALSE
560	1.60E-24	FALSE	FALSE
560	1.60E-24	FALSE	FALSE
560	1.60E-24	FALSE	FALSE
560	1.60E-24	FALSE	FALSE
560	3.52E-17	FALSE	FALSE
602	6.68E-06	FALSE	FALSE
602	4.07E-06	FALSE	FALSE
602	4.07E-06	FALSE	FALSE
602	4.07E-06	FALSE	FALSE
602	2.66E-06	FALSE	FALSE
933	1.41E-13	FALSE	FALSE
933	2.73E-13	FALSE	FALSE
933	1.16E-10	FALSE	FALSE
933	8.15E-15	FALSE	FALSE
933	1.82E-13	FALSE	FALSE
933	1.60E-10	FALSE	FALSE
933	9.47E-14	FALSE	FALSE
933	3.11E-13	FALSE	FALSE
933	3.36E-13	FALSE	FALSE
1096	1.34E-10	FALSE	FALSE
1096	1.92E-10	FALSE	FALSE
1096	1.92E-10	FALSE	FALSE
1096	1.34E-10	FALSE	FALSE
1096	5.72E-11	FALSE	FALSE
1096	5.72E-11	FALSE	FALSE
1096	1.24E-09	FALSE	FALSE
1096	5.72E-11	FALSE	FALSE
1096	1.79E-10	FALSE	FALSE
1096	1.15E-06	FALSE	FALSE
1096	1.92E-10	FALSE	FALSE
1096	1.15E-06	FALSE	FALSE

1096	3.73E-07	FALSE	FALSE
1096	4.36E-06	FALSE	FALSE
1096	1.15E-06	FALSE	FALSE
1096	1.79E-10	FALSE	FALSE
1096	2.74E-09	FALSE	FALSE
1096	2.74E-09	FALSE	FALSE
1500	7.92E-09	FALSE	FALSE
1500	7.92E-09	FALSE	FALSE
1500	7.92E-09	FALSE	FALSE
1500	7.92E-09	FALSE	FALSE
1509	1.34E-08	FALSE	FALSE
1509	5.25E-19	FALSE	FALSE
1509	2.30E-10	FALSE	TRUE
1509	2.30E-10	FALSE	TRUE
1509	1.46E-13	FALSE	FALSE
1509	7.29E-19	FALSE	TRUE
1509	5.25E-19	FALSE	FALSE
1509	2.57E-12	FALSE	FALSE
1509	5.25E-19	FALSE	TRUE
1509	5.25E-19	FALSE	TRUE
1509	5.25E-19	TRUE	TRUE
1509	5.25E-19	FALSE	TRUE
1509	5.25E-19	FALSE	TRUE
1509	5.25E-19	FALSE	TRUE
1509	5.25E-19	FALSE	FALSE
1509	5.25E-19	FALSE	FALSE
1509	5.25E-19	FALSE	FALSE
1509	5.25E-19	FALSE	FALSE
1509	5.25E-19	FALSE	TRUE
1509	5.25E-19	FALSE	TRUE
1509	2.76E-17	FALSE	FALSE
1584	1.47E-08	FALSE	TRUE
1584	1.47E-08	FALSE	FALSE

F_INTEREST

Supplementary Table 10. Latent causal variable results forl causal association between PD				
**Phenotype 1**	**Phenotype 2**	**GCP**	**SE**	**P**
PD	ICV	-0.31	0.14	4.06E-02
PD	Pallidum	-0.23	0.21	5.23E-01
PD	Putamen	-0.49	0.15	2.00E-06
PD	Ventral diencephalon	-0.11	0.09	3.07E-01
PD	Accumbens	-0.21	0.44	8.20E-01
PD	Caudate	-0.55	0.27	6.00E-02
PD	Brainstem	-0.04	0.10	7.83E-01
PD	Thalamus	-0.20	0.18	2.96E-01

Cells in green highlight significant p-values after Bonferroni multiple testing corretion.

Supplementary Table 11. GSMR results for bi-directional causal association between PD			
**Exposure**	**Outcome**	**Beta**	**SE**
Accumbens	PD	0.05	0.06
PD	Accumbens	0.00	0.01
Brainstem	PD	0.12	0.04
PD	Brainstem	0.05	0.01
Caudate	PD	0.18	0.05
PD	Caudate	0.04	0.01
ICV	PD	0.54	0.05
PD	ICV	0.02	0.01
Pallidum	PD	0.35	0.08
PD	Pallidum	0.02	0.01
Putamen	PD	0.32	0.05
PD	Putamen	0.03	0.01
Thalamus	PD	0.13	0.06
PD	Thalamus	0.01	0.01
Ventral diencephalon	PD	0.23	0.05
PD	Ventral diencephalon	0.01	0.01

Cells in green highlight significant p-values after Bonferroni multiple testing corretion.

D and intracranial and subcortical brain volumes		
P	Number of SNPs	Global HEIDI outlier
3.87E-01	19	0.12
7.13E-01	44	0.14
1.34E-03	36	0.06
1.34E-05	34	0.07
5.73E-05	35	0.08
3.48E-05	42	0.24
2.33E-32	37	0.10
1.07E-01	40	0.08
7.81E-06	15	0.09
6.27E-02	39	0.07
2.51E-11	30	0.11
5.26E-03	40	0.08
3.78E-02	18	0.07
1.42E-01	44	0.06
1.68E-06	19	0.11
5.66E-01	41	0.06

Supplementary Table 12. Multivariate Mendelian randomisation results for causal associati			
**Exposure**	**Outcome**	**Beta**	**SE**
Pallidum + controlling for ICV	PD	0.205	0.08
ICV + controlling for pallidum	PD	0.41	0.059
Putamen + controlling for ICV	PD	0.159	0.057
ICV + controlling for putamen	PD	0.423	0.059
Ventral diencephalon + controlling for	PD	0.29	0.084
ICV + controlling for ventral	PD	-0.098	0.07
Brainstem + controlling for ICV	PD	0.101	0.043
ICV + controlling for brainstem	PD	0.391	0.053
Caudate + controlling for ICV	PD	0.161	0.058
ICV + controlling for caudate	PD	0.409	0.056

ions between PD and intracranial and	
**P**	**Number of SNPs**
1.00E-02	100
4.59E-12	100
5.40E-03	133
1.10E-12	133
5.70E-04	89
1.62E-01	89
1.80E-02	127
3.60E-13	127
2.80E-13	132
3.60E-03	132

Supplementary Table 13. Genetic correlation results between Alzheimer's disease and				
**Phenotype**	**rG**	**SE**	**p-value**	**CI**
Thalamus	-0.10	0.05	0.03	0.09
Hippocampus	-0.09	0.05	0.08	0.10
Amygdala	-0.08	0.05	0.12	0.11
Putamen	-0.06	0.04	0.16	0.09
Accumbens	-0.05	0.05	0.31	0.09
ICV	-0.03	0.04	0.43	0.08
Ventral diencephal	0.03	0.05	0.57	0.09
Caudate	-0.02	0.04	0.59	0.09
Pallidum	-0.02	0.05	0.77	0.10
Brainstem	-0.01	0.05	0.79	0.09

Supplementary Table 14. Full GWAS-pairwise results showing that no genomic segments				
phenotype	chr	start_basepair	stop_basepair	PPA model 3
Hippocampus	2	14004	776007	0.001441
Hippocampus	5	21529	26849	0.000117
Hippocampus	6	202452	904662	0.000924
Hippocampus	7	40483	203917	0.000198
Hippocampus	9	202004	1077149	0.001203
Hippocampus	9	1080616	1911291	0.000251
Hippocampus	9	1919632	3189696	0.000267
Hippocampus	9	3190407	4495254	0.00938
Hippocampus	9	4495328	4884849	0.000236
Hippocampus	9	4885082	6557414	0.000503
Hippocampus	9	6557589	7154294	0.000228
Hippocampus	9	7154923	8456135	0.000908
Hippocampus	9	8457691	9165730	0.000803
Hippocampus	9	9166788	10879071	0.000291
Hippocampus	9	10880887	12274774	0.000253
Hippocampus	9	12280057	12712441	0.000554
Hippocampus	11	196944	1076149	0.000235
Hippocampus	18	127917	764152	0.000323
Hippocampus	18	2156130	3890554	0.000203
Hippocampus	18	3894971	4738152	0.000332
Hippocampus	19	255541	607108	0.000749
Hippocampus	19	612940	992903	0.000231
Putamen	2	18674	776007	0.001006
Putamen	5	21529	26849	7.92E-05
Putamen	6	202452	904662	0.000385
Putamen	7	40483	203326	9.01E-05
Putamen	9	202004	1077149	0.000864
Putamen	9	1080616	1911291	0.000447
Putamen	9	1919632	3189696	0.000312
Putamen	9	3190407	4495254	0.000856
Putamen	9	4495328	4884849	0.000212
Putamen	9	4885082	6557414	0.000227
Putamen	9	6557589	7154294	0.001659
Putamen	9	7154923	8456135	0.001255
Putamen	9	8457691	9165730	0.000384
Putamen	9	9166788	10879071	0.000364
Putamen	9	10880887	12274774	0.000249
Putamen	9	12280057	12712441	0.00046
Putamen	11	196944	1076149	0.000236
Putamen	18	128929	764152	0.000439
Putamen	18	2156130	3890554	0.000603
Putamen	18	3895121	4738152	0.000198
Putamen	19	256859	607108	0.000142
Putamen	19	612940	992903	0.000269
VentralDC	2	11357	776071	0.002455
VentralDC	5	13018	26849	0.000142
VentralDC	6	202452	904662	0.00101
VentralDC	7	34713	232411	0.000419
VentralDC	9	58427	1079680	0.000777
VentralDC	9	1080616	1916209	0.000615

VentralDC	9	1919555	3189806	0.00116
VentralDC	9	3190407	4495254	0.011345
VentralDC	9	4495328	4884849	0.006323
VentralDC	9	4884965	6557414	0.00163
VentralDC	9	6557589	7154294	0.000748
VentralDC	9	7154923	8456135	0.0013
VentralDC	9	8456589	9165998	0.000878
VentralDC	9	9166788	10879071	0.00065
VentralDC	9	10880378	12275851	0.000555
VentralDC	9	12276932	12712441	0.000645
VentralDC	11	196944	1076149	0.000788
VentralDC	18	48582	764152	0.000509
VentralDC	18	2156130	3891039	0.0012
VentralDC	18	3891682	4738152	0.000887
VentralDC	19	244421	609941	0.001792
VentralDC	19	612234	993313	0.003181
Caudate	2	18674	776007	0.001417
Caudate	5	21529	26849	0.000153
Caudate	6	202452	904662	0.000484
Caudate	7	40483	203326	0.000126
Caudate	9	202004	1077149	0.000197
Caudate	9	1080616	1911291	0.001264
Caudate	9	1919632	3189696	0.000343
Caudate	9	3190407	4495254	0.000357
Caudate	9	4495328	4884849	0.000192
Caudate	9	4885082	6557414	0.000206
Caudate	9	6557589	7154294	0.000156
Caudate	9	7154923	8456135	0.000833
Caudate	9	8457691	9165730	0.000259
Caudate	9	9166788	10879071	0.000237
Caudate	9	10880887	12274774	0.000231
Caudate	9	12280057	12712441	0.000758
Caudate	11	196944	1076149	0.000149
Caudate	18	128929	764152	0.000127
Caudate	18	2156130	3890554	0.000405
Caudate	18	3895121	4738152	0.000147
Caudate	19	256859	607108	0.000193
Caudate	19	612940	992903	0.000566
Amygdala	2	14004	776007	0.001276
Amygdala	5	21529	26849	0.000666
Amygdala	6	202452	904662	0.000723
Amygdala	7	40483	203917	0.000158
Amygdala	9	202004	1077149	0.000147
Amygdala	9	1080616	1911291	0.000399
Amygdala	9	1919632	3189696	0.000426
Amygdala	9	3190407	4495254	0.002013
Amygdala	9	4495328	4884849	0.000169
Amygdala	9	4885082	6557414	0.000234
Amygdala	9	6557589	7154294	0.000245
Amygdala	9	7154923	8456135	0.001066
Amygdala	9	8457691	9165730	0.000376
Amygdala	9	9166788	10879071	0.000431

Amygdala	9	10880887	12274774	0.000209
Amygdala	9	12280057	12712441	0.000402
Amygdala	11	196944	1076149	0.000936
Amygdala	18	128929	764152	0.000583
Amygdala	18	2156130	3890554	0.000253
Amygdala	18	3894971	4738152	0.000136
Amygdala	19	255499	607108	0.000368
Amygdala	19	612940	992903	0.000345
Accumbens	2	14004	776007	0.001202
Accumbens	5	21529	26849	9.15E-05
Accumbens	6	202452	904662	0.000704
Accumbens	7	40483	203917	0.000121
Accumbens	9	202004	1077149	0.000216
Accumbens	9	1080616	1911291	0.000592
Accumbens	9	1919632	3189696	0.000546
Accumbens	9	3190407	4495254	0.013473
Accumbens	9	4495328	4884849	0.000242
Accumbens	9	4885082	6557414	0.000276
Accumbens	9	6557589	7154294	0.000228
Accumbens	9	7154923	8456135	0.020897
Accumbens	9	8457691	9165730	0.000298
Accumbens	9	9166788	10879071	0.000422
Accumbens	9	10880887	12274774	0.000301
Accumbens	9	12280057	12712441	0.0006
Accumbens	11	196944	1076149	0.000662
Accumbens	18	128929	764152	0.000466
Accumbens	18	2156130	3890554	0.002456
Accumbens	18	3894971	4738152	0.000313
Accumbens	19	254927	607108	0.000243
Accumbens	19	612940	992903	0.000224
Thalamus	2	14004	776007	0.00113
Thalamus	5	21529	26849	8.50E-05
Thalamus	6	202452	904662	0.000428
Thalamus	7	40483	203917	0.000504
Thalamus	9	202004	1077149	0.000426
Thalamus	9	1080616	1911291	0.000319
Thalamus	9	1919632	3189696	0.000279
Thalamus	9	3190407	4495254	0.000333
Thalamus	9	4495328	4884849	0.000229
Thalamus	9	4885082	6557414	0.00031
Thalamus	9	6557589	7154294	0.000181
Thalamus	9	7154923	8456135	0.009043
Thalamus	9	8457691	9165730	0.000229
Thalamus	9	9166788	10879071	0.00138
Thalamus	9	10880887	12274774	0.004558
Thalamus	9	12280057	12712441	0.000438
Thalamus	11	196944	1076149	0.006712
Thalamus	18	128929	764152	0.000471
Thalamus	18	2156130	3890554	0.000424
Thalamus	18	3894971	4738152	0.000182
Thalamus	19	255499	607108	0.000569
Thalamus	19	612940	992903	0.001516

Brainstem	2	14004	776007	0.002679
Brainstem	5	21529	26849	0.000182
Brainstem	6	202452	904662	0.000534
Brainstem	7	40483	203326	0.000146
Brainstem	9	202004	1077149	0.001402
Brainstem	9	1080616	1911291	0.000459
Brainstem	9	1919632	3189696	0.000291
Brainstem	9	3190407	4495254	0.0028
Brainstem	9	4495328	4884849	0.003567
Brainstem	9	4885082	6557414	0.000309
Brainstem	9	6557589	7154294	0.000208
Brainstem	9	7154923	8456135	0.000844
Brainstem	9	8457691	9165730	0.000271
Brainstem	9	9166788	10879071	0.000646
Brainstem	9	10880887	12274774	0.000273
Brainstem	9	12280057	12712441	0.001503
Brainstem	11	196944	1076149	0.001588
Brainstem	18	128929	764152	0.006826
Brainstem	18	2156130	3890554	0.000828
Brainstem	18	3894971	4738152	0.000659
Brainstem	19	253938	607108	0.000218
Brainstem	19	612940	992903	0.001002
ICV	2	18856	776007	0.001303
ICV	5	21529	26849	0.000681
ICV	6	202452	904662	0.003729
ICV	7	40483	203917	0.000125
ICV	9	202004	1077149	0.000438
ICV	9	1080616	1911291	0.000471
ICV	9	1919632	3189696	0.000536
ICV	9	3190407	4495254	0.006598
ICV	9	4495328	4884849	0.000381
ICV	9	4885082	6557414	0.000298
ICV	9	6557589	7154294	0.000292
ICV	9	7154923	8456135	0.001
ICV	9	8457691	9165730	0.00093
ICV	9	9166788	10879071	0.000405
ICV	9	10880887	12274774	0.000416
ICV	9	12280057	12712441	0.00067
ICV	11	196944	1076149	0.000374
ICV	18	127917	764152	0.000225
ICV	18	2156130	3890554	0.000189
ICV	18	3894971	4738152	0.000171
ICV	19	255541	607108	0.000432
ICV	19	612940	992903	0.005265
Pallidum	2	14004	776007	0.002094
Pallidum	5	21529	26849	7.91E-05
Pallidum	6	202452	904662	0.000495
Pallidum	7	40483	203917	0.000164
Pallidum	9	202004	1077149	0.000378
Pallidum	9	1080616	1911291	0.003514
Pallidum	9	1919632	3189696	0.000253
Pallidum	9	3190407	4495254	0.000219
